# Statistical analysis plan for the multicenter, open, randomized controlled clinical trial to assess the efficacy and safety of intravenous tirofiban vs aspirin in acute ischemic stroke due to tandem lesion, undergoing recanalization therapy by endovascular treatment (ATILA trial)

**DOI:** 10.1186/s13063-023-07817-9

**Published:** 2024-01-09

**Authors:** Elena Zapata-Arriaza, Manuel Medina-Rodríguez, Francisco Moniche Álvarez, Asier de Albóniga-Chindurza, Marta Aguilar-Pérez, Leire Ainz-Gómez, Pablo Baena-Palomino, Aynara Zamora, Blanca Pardo-Galiana, Fernando Delgado-Acosta, Roberto Valverde Moyano, Elvira Jiménez-Gómez, Isabel Bravo Rey, Rafael Oteros Fernández, Irene Escudero-Martínez, Isabel Vielba-Gomez, Lluis Morales Caba, Jose Díaz Pérez, Estefania García Molina, Sonia Mosteiro, María del Mar Castellanos Rodrigo, Laura Amaya Pascasio, Carlos Hidalgo, María del Mar Freijo Guerrero, Eva González Díaz, Jose María Ramírez Moreno, Luis Fernández Prudencio, Mikel Terceño Izaga, Saima Bashir Viturro, Miguel Ángel Gamero-García, Silvia Jiménez Jorge, Clara Rosso Fernández, Joan Montaner, Alejandro González García

**Affiliations:** 1 Stroke Unit, Neurovascular Research Program, Seville Biomedical Research Institute, Seville, Spain; 2 Interventional Neuroradiology Department, Virgen del Rocío University Hospital, Neurovascular Research Laboratory, Institute of Biomedicine of Seville (IBIS), Av Manuel Siurot sn, Seville, 41013 Spain; 3 Neurology Department, Virgen del Rocío University Hospital, Sevilla, Spain; 4 Interventional Neuroradiology Department, Reina Sofía University Hospital, Córdoba, Spain; 5 Neurology Department, Reina Sofía University Hospital, Córdoba, Spain; 6 Neurology Department, La Fe University and Polytechnic Hospital, Valencia, Spain; 7 Interventional Neuroradiology Department, La Fe University and Polytechnic Hospital, Valencia, Spain; 8 Interventional Neuroradiology Department, Virgen de la Arrixaca University Clinical Hospital, Murcia, Spain; 9 Neurology Department, Virgen de la Arrixaca University Clinical Hospital, Murcia, Spain; 10 Interventional Neuroradiology Department, A Coruña University Hospital Complex, Coruña, Spain; 11 Neurology Department, A Coruña University Hospital Complex, A Coruña, Spain; 12 Neurology Department, Torrecardenas University Hospital, Almería, Spain; 13 Interventional Neuroradiology Department, Torrecardenas University Hospital, Almería, Spain; 14 Neurology Department, Cruces University Hospital, Vizcaya, Spain; 15 Interventional Neuroradiology Department, Cruces University Hospital, Vizcaya, Spain; 16 Neurology Department, Badajoz University Hospital, Badajoz, Spain; 17 Interventional Neuroradiology, Badajoz University Hospital, Badajoz, Spain; 18 Department of Neurology, Doctor Josep Trueta Hospital, Girona, Spain; 19 Interventional Neuroradiology Unit, Doctor Josep Trueta Hospital, Girona, Spain; 20 Neurology Department, Virgen Macarena University Hospital, Seville, Spain; 21 Clinical Research and Clinical Trials Unit (CTU), Virgen del Rocío Hospital, Seville, Spain

**Keywords:** Protocol, Stroke, Tandem occlusion, Statistics, Antiplatelet therapy

## Abstract

**Rationale:**

In-stent reocclusion after endovascular therapy has a negative impact on outcomes in acute ischemic stroke (AIS) due to tandem lesions (TL). Optimal antiplatelet therapy approach in these patients to avoid in-stent reocclusion is yet to be elucidated.

**Aims:**

To assess efficacy and safety of intravenous tirofiban versus intravenous aspirin in patients undergoing MT plus carotid stenting in the setting of AIS due to TL.

**Sample size estimates:**

Two hundred forty patients will be enrolled, 120 in every treatment arm.

**Methods and design:**

A multicenter, prospective, randomized, controlled (aspirin group), assessor-blinded clinical trial will be conducted. Patients fulfilling the inclusion criteria will be randomized at MT onset to the experimental or control group (1:1). Intravenous aspirin will be administered at a 500-mg single dose and tirofiban at a 500-mcg bolus followed by a 200-mcg/h infusion during the first 24 h. All patients will be followed for up to 3 months.

**Study outcomes:**

Primary efficacy outcome will be the proportion of patients with carotid in-stent thrombosis within the first 24 h after MT. Primary safety outcome will be the rate of symptomatic intracranial hemorrhage.

**Discussion:**

This will be the first clinical trial to assess the best antiplatelet therapy to avoid in-stent thrombosis after MT in patients with TL.

**Trial registration:**

The trial is registered as NCT05225961. February, 7th, 2022.

**Supplementary Information:**

The online version contains supplementary material available at 10.1186/s13063-023-07817-9.

## Introduction and rationale

Acute ischemic stroke (AIS) due to tandem lesions (TL) represents approximately 20% of AIS involving anterior circulation undergoing mechanical thrombectomy (MT) [1]. Recent studies regarding TL showed similar functional outcomes and rates of symptomatic intracranial hemorrhage (SICH) compared to isolated intracranial occlusions [2–4]. The need for intracranial recanalization and its positive impact on the functional outcome of the patient has been widely demonstrated [5]. However, large clinical trials [6–10] hardly included patients with tandem occlusion, and extracranial management is uncertain, requiring randomized trials to clarify the best management. The main dilemma posed by extracranial lesions in the acute phase is the placement of carotid stents, in which case the use of antiplatelet agents is necessary. All the same, such antiplatelet therapy increases the risk of sICH, and its use in combination with IVF is currently contraindicated until 90 min after fibrinolytic administration [11]. Nevertheless, failure to place the carotid stent can lead to extracranial artery reocclusion, lengthening the procedure and exposing the patient to the risk of a new intracranial embolism [12]. There is current and growing evidence which demonstrates the positive impact of stent use. Among the benefits described in the TL stenting group, we found a lower rate of reocclusion and better rates of good functional prognosis at 90 days without a significant increase in the rate of sICH [1, 13, 14]. Thus, the use of stents appears to be effective and yields encouraging results. In any case, the use of stents in the acute phase, despite showing encouraging results, associates significant issues regarding the best antiplatelet treatment.

Up to now, there is no consensus regarding the best therapy of the extracranial lesion. The antithrombotic management used in the main published studies [1, 15–17] does not show a homogeneous standardization, and the safety results described show variable but encouraging data on mortality and sICH. The possibilities include from not administering any antiplatelet agent, mono-antiplatelet with aspirin or acetylsalicylic acid (ASA), clopidogrel or glycoprotein IIb-IIa iv inhibitors, or the combination of some of them. The main concern of the use of antithrombotics is their combination with IV fibrinolytic, although based on the studies described previously, the use of IVF is widespread and does not associate higher rates of sICH.

Intravenous (IV) ASA irreversibly acetylates the enzyme cyclooxygenase-1 preventing the conversion of arachidonic acid to thromboxane A2 (potent promoter of platelet aggregation) in a dose-dependent manner [18, 19]. It starts its action in 15–30 min, and its half-life in plasma is approximately 15–20 min [18, 19]. One of the main limitations in aspirin use is the high rate of resistance to the drug (up to 30% in some studies) [18, 19]. This resistance is due to multiple interindividual factors (use of NSAIDs, diabetes, hyperthyroidism, etc.) difficult to control in the acute phase of endovascular treatment. Aspirin in doses of 500 mg/iv is probably the most widely used [11], given the widespread experience and its availability via intravenous route that facilitates its administration.

As an glycoprotein IIb/IIIa inhibitor, tirofiban is a non-peptide platelet GP IIb/IIIa receptor antagonist (which is the most abundant integrin on the surface of platelets), effectively blocking GP IIb/IIIa receptors with high selectivity by preventing fibrinogen binding to platelets and subsequent platelet aggregation at the site of atherosclerosis [18, 19]. Intravenous bolus tirofiban of 10 μg/kg followed by 0.10 to 0.15 μg/kg/min has demonstrated an inhibition of platelet aggregation ex vivo by > 90% in 5 min. Platelet aggregation is restored to approximately 50% at 4 h after cessation of continuous infusion and reaches levels close to baseline after 8 h. This dose-dependent blocking effect is rapidly metabolized following cessation of intravenous infusion and involves a short plasma half-life (approximately 2 h). The effect of tirofiban is not affected by genetic polymorphisms, and so far, no significant rates of drug resistance have been published. The 2019 AHA guidelines [11] specify that the efficacy of tirofiban co-administered with rtPA iv is not well established (with recommendation IIb) and should be administered in the context of clinical trials. It has the added advantage of being administered intravenously and has been used in some centers to prevent local platelet aggregation and early reocclusion [20–22]. Likewise, there is already evidence of clinical benefit from the use of tirofiban, even when combined with rtPA without increasing the risk of sICH or mortality [21]. The use of low-dose tirofiban (500 µg bolus plus infusion of 200 µg per hour, for 18 h) [20] has demonstrated its safety and efficacy in the endovascular treatment of patients with acute ischemic stroke. Simple antiplatelet therapy with tirofiban in patients with AIS and TL may be a reasonable alternative. Tirofiban might be an option when aspirin is not available or ineffective (level of evidence IIb). Nevertheless, the effect of tirofiban has not been well studied in the setting of AIS.

In summary, there is currently no standard criterion for the antiplatelet therapy of choice in acute stroke patients with tandem lesions. Current guidelines contraindicate the use of intravenous aspirin in the first 90 min after administration of intravenous fibrinolysis, which limits the antiplatelet therapy essential to address TL. The use of antithrombotics such as glycoprotein IIb/IIIa inhibitors is recommended in clinical trials. Our hypothesis postulates that in patients with acute stroke and TL in the first 24 h who require the placement of an extracranial stent, treatment with tirofiban is safe and decreases the rate of acute occlusions of the stent in the first 24 h compared to standard antiplatelet therapy with ASA.

## Study purpose

The study purpose is to test the hypothesis that low-dose protocol of IV-tirofiban is superior to IV-aspirin in avoiding in-stent thrombosis at 24 h in patients undergoing MT plus carotid stenting in the setting of AIS due to TL.

## Methods

### Design

The design is a multicenter, prospective, randomized, controlled (ASA group as control), assessor-blinded clinical trial of IV-tirofiban *superiority* vs IV-aspirin in AIS due to TL undergoing MT. The protocol is registered in http://clinicaltrials.gov (NCT05225961 on February 7, 2022) and authorized by the Spanish Regulatory Agency (AEMPS) (Eudra-CT: 2021-003874-30) and the central ethic committee (EC) for all the participating sites (act number 03/2022).

### Patient population–inclusion and exclusion criteria

Patients with AIS of < 24 h from stroke onset, involving the anterior circulation secondary to a TL and candidates for MT according to clinical practice guidelines (CPG) that require the placement of a carotid stent during the procedure, will be included. Detailed inclusion and exclusion criteria are shown in the Supplementary Materials. All patients must sign informed consent before inclusion.

### Randomization

**Fig. 1:**
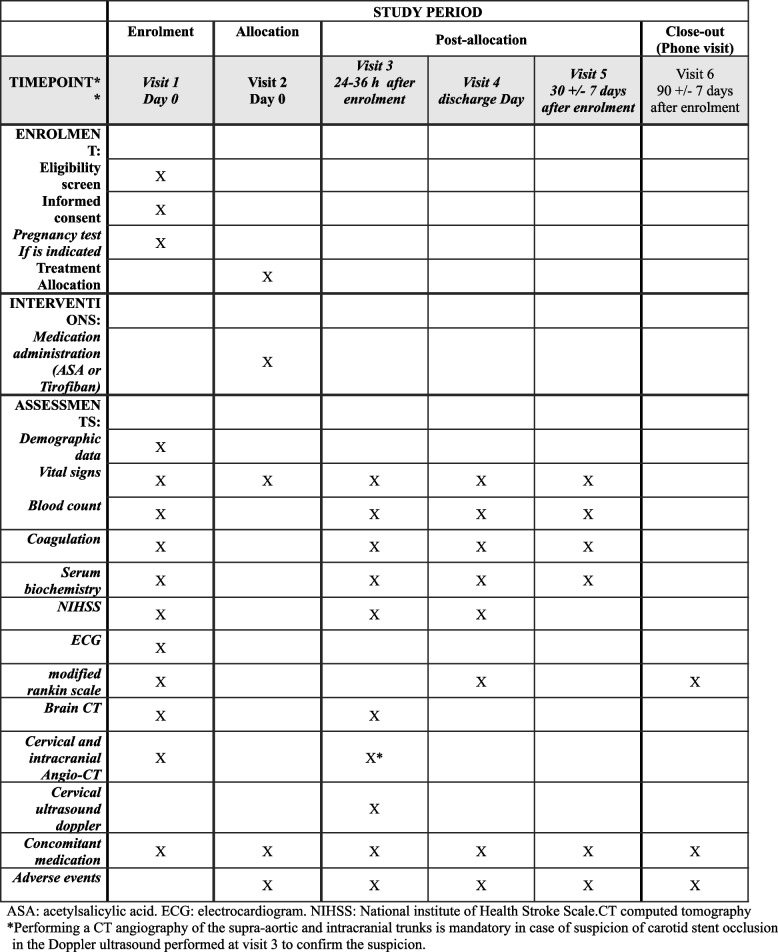
SPIRIT schematic diagram. ASA, acetylsalicylic acid; ECG, electrocardiogram; NIHSS, National institute of Health Stroke Scale; CT, computed tomography. Asterisk (*) symbol indicates the following: performing a CT angiography of the supra-aortic and intracranial trunks is mandatory in case of suspicion of carotid stent occlusion in the Doppler ultrasound performed at visit 3 to confirm the suspicion

After informed consent by the patient or relatives is obtained, patients will be randomized to either the control group (IV-aspirin) or experimental group (IV-tirofiban) (experimental or control in ratio 1:1). Patients can be randomized at any time within baseline neuroimage and before groin puncture. A blinded clinician, certified in carotid ultrasound and in assessment of the modified Rankin Scale (mRS), will undertake assessment of the primary outcomes by clinical visit. Figure 1 shows the SPIRIT schematic diagram about scheduled visits including the enrolment, interventions, and necessary assessments within the clinical trial.

Randomization will take place by a remote, web-based, computer-generated randomization procedure. All online submissions are secured by the use of password site entry and data encryption procedures via the REDCap online webpage. Investigators record patient details via a secure web interface before randomization takes place. The randomization procedure includes a standard minimization algorithm which ensures that the treatment groups are balanced across all centers in all regions. Patients are allocated to treatment groups in order to minimize the difference between the groups with regard to the key prognostic factors. Patients will be randomly allocated to open-label IV tirofiban or to a control group (aspirin).

### Sample size considerations

To calculate the sample size, we must state that there is no explicit literature that has made a previous comparison between the use of aspirin or tirofiban with other comparators, against placebo or with each other to prevent in-stent reocclusion in the study population group. Recent studies [14] show carotid reocclusion rates of 22% within the first 24 h of stent placement when using a loading dose of 900 mg of Inyesprin (intravenous ASA) and adding at the end of the endovascular procedure the administration of the loading dose of oral clopidogrel (300 mg). In our case, we intend to confirm our hypothesis of a lower rate of reocclusion when using tirofiban in mono antiplatelet therapy, compared to the extensive and habitual use of aspirin as the generalized drug of choice in the first 24 h after the onset of ischemic stroke symptoms. Bearing in mind that the aforementioned bibliography uses intravenous aspirin as the antiplatelet agent of choice during thrombectomy, we intend to use their data as a starting point for calculating the sample size. For this reason, we took a 22% reocclusion rate as a reference using Inyesprin as the first choice compared to an expected reduction of at least 12% with tirofiban (10% of reocclusion) to obtain a significant reduction with clinical impact in the carotid reocclusion rate.

To calculate the sample size, we employed the free online application Granmo sample size calculator (https://www.imim.cat/ofertadeserveis/software-public/granmo/) which uses the approximation of the ARCOSENE for paired measurements (repeated in one group). The following parameters were displayed: power 80%, alpha error 5%, estimated difference between two proportions of 22% (for the control group, ASA) and 10% (for the experimental group, tirofiban). With these considerations, and including a 5% loss, the sample size would be equal to 240 patients (120 in each group).

### Statistical analysis plan

#### Analysis principles and general considerations

In the first phase, an exhaustive exploratory analysis will be carried out, which will be accompanied by the corresponding tables and graphical outputs, with the main aim to verify that there is no erroneous value and to characterize the sample object of study. An analysis of the missing data will be carried out to assess the percentage of losses in the different moments of the study and to identify if mentioned loss has randomly produced. The results will be analyzed once the monitoring of the data of all included patients is completed and always after at least 4 months from the inclusion of the last patient. With this time frame, we intend for the last follow-up visit of the last recruited patient to have been completed. The measurement of the results will be carried out by the Spanish Clinical Research Network (SCReN) platform of the Carlos III Health Institute of Spain.

All analyses will be conducted on data from all randomly assigned patients according to the intention-to-treat (ITT) principle, i.e., patients will be analyzed in the group they were randomized to, no matter what treatment they received, and regardless of whether they deviated from the protocol in any way. In addition, a comparison of treatment groups that includes only those patients who completed the treatment originally allocated, following per-protocol analysis, will be conducted too. All outcomes and analyses are prospectively categorized as primary, secondary, or exploratory.

A descriptive analysis of all the demographic variables collected as well as clinical data prior to the start of treatment will be carried out. The quantitative variables will be presented in absolute and relative values and the quantitative variables through measures of centrality, position, or variability. The verification of the assumptions of normality of the quantitative variables will be carried out with the Shapiro-Wilk or Kolmogorov test. The comparison of quantitative variables was done with the *T* test or the Mann-Whitney test for two groups and the relationship of qualitative variables with the chi-square test, or, if the expected frequencies in 20% of the cells are < 5, the Fisher test was used. The significance level of the test was set at 0.005.

Ordinal logistic regression is pre-specified as the method for the analysis of the primary outcome using the common odds ratio as one of the effect size measures, restricting adjustment to age, symptom severity (baseline NIHSS score), and intravenous thrombolysis. Covariate adjustment variables are continuous and categorical variables, with assumed linearity and nonlinearity being allowed. Non-linearity will only be included if the addition of the squared variable significantly improves the model fit (judged by an improvement in the likelihood ratio test). In addition, treatment effects will be calculated as absolute increase in the probability for carotid reocclusion outcome/absolute risk reduction (ARR) in percentage points and with number needed to treat (NTT) defined as 1/ARR.

Differences in all outcomes between the two treatment groups will be tested independently at the two-tailed 0.05 level of significance. All estimates of treatment effects will be presented with 95% confidence intervals (CIs). No formal adjustments will be undertaken to reduce the overall type I error associated with both secondary and exploratory analyses including the subgroup analyses. Their purpose is to supplement evidence from the primary analysis to better characterize the treatment effect.

Results from the secondary and exploratory analyses will be interpreted in this context. Pre-specified subgroup analyses (Supplementary Materials) will be carried out irrespective of whether there is a significant treatment effect on the primary outcome.

Sensitivity analyses for the primary outcome will be undertaken to test the robustness of the primary analysis with regard to protocol violations, baseline imbalance, clustering effects, and missing data. Unadjusted analysis will be undertaken as sensitivity analysis and will be presented for all primary and secondary outcomes.

Analyses will be conducted primarily using the SPSS 26 and R-4.3.1 statistical computing and graphics software.

#### Hypothesis testing framework

Research hypothesis for logistic regression with additional adjustments:

– H0 (null hypothesis): There is no significant difference in the risk of stent thrombosis between the tirofiban-treated group and the control group, after adjusting for age, baseline NIHSS score, and intravenous fibrinolysis. OR = 1

– H1 (alternative hypothesis): There is a significant difference in the risk of stent thrombosis between the tirofiban-treated group and the control group, after adjusting for age, baseline NIHSS score, and intravenous fibrinolysis. OR different from 1

Statistical significance will be evaluated using a significance level (*α*) set at *p* < 0.05.

#### Treatment of missing values

Rigorous efforts are made to minimize the amount of missing outcome data. Hospitals with committed researchers and experience in patient inclusion and follow-up have been chosen. Once included in the study, the researchers explain to the patient and their family what the visit schedule will be, so that they become aware of it, emphasizing the importance for their evolution of maintaining said clinical follow-up. Those patients who are passing through our country/region for vacations, work, etc., raise questions about possible loss of follow-up. All candidate patients and their family (regardless of their origin) are explained the possibility of participating in the trial before their inclusion. In this case, they will be required to remain in the region of the hospital where they are admitted during the first month after inclusion. Visit 5 (30 days post-randomization) must be carried out in person, and they can return to their country of origin later, since visit 6 is carried out by telephone. All of this is explained to the family and/or to the patient (if is conscious). If the family/patient refuses to participate due to the need to return to their country or region, the patient is not included, thus reducing loss to follow-up. In this way, both the patient and the family are given the possibility of participating, explaining to them in advance the organization chart of the trial so that they can decide autonomously. All this will allow us to reduce follow-up losses to a minimum. Minimal loss to follow-up for the 3 months assessment of the primary outcome is anticipated. If functional status at 3 months is unknown for any patient, we will apply the following algorithm: if the patient was alive at 3 months and measurements are available after baseline, we will use the level of function recorded on the day corresponding to the last trial visit performed; this could be the discharge visit or the month after the mechanical thrombectomy (i.e., measured at discharge from hospital or one month after mechanical thrombectomy) to impute functional status at 3 months. Hence, 3 months mRS will be imputed for patients with status at discharge day or 1 month after mechanical thrombectomy, always choosing the most recent one for which we have functional data. We have chosen this simple form of single imputation, as it usually classifies patients appropriately, where discharge day and 3 months data are known, and any additional gain from more complex multiple imputation methods is likely to be small [23]. The mRS outcome is assumed to be missing-at-random. Important explanatory and auxiliary variables such as age, baseline NIHSS score, geographical region, time of randomization after symptoms onset, and treatment group are being collected and will be examined to assess the plausibility of the missing at random assumption. No missing data are expected in adjustment covariates as completeness is assured by the web-based randomization procedure. Any missing components of the primary efficacy (stent acute thrombosis within the first 24 h) and safety (symptomatic intracranial hemorrhage or sICH within the first 36 h after randomization) endpoints will be imputed by sensitivity analysis of the multiple imputation type → iteration model of missing data.

Sensitivity analyses based on different hypotheses about the missingness pattern of the primary outcome will be conducted to test for the robustness of the primary outcome, including analysis of the “complete case population,” i.e., based on the data of completers, using only observed without accommodating missing data.

#### Trial profile

**Fig. 2:**
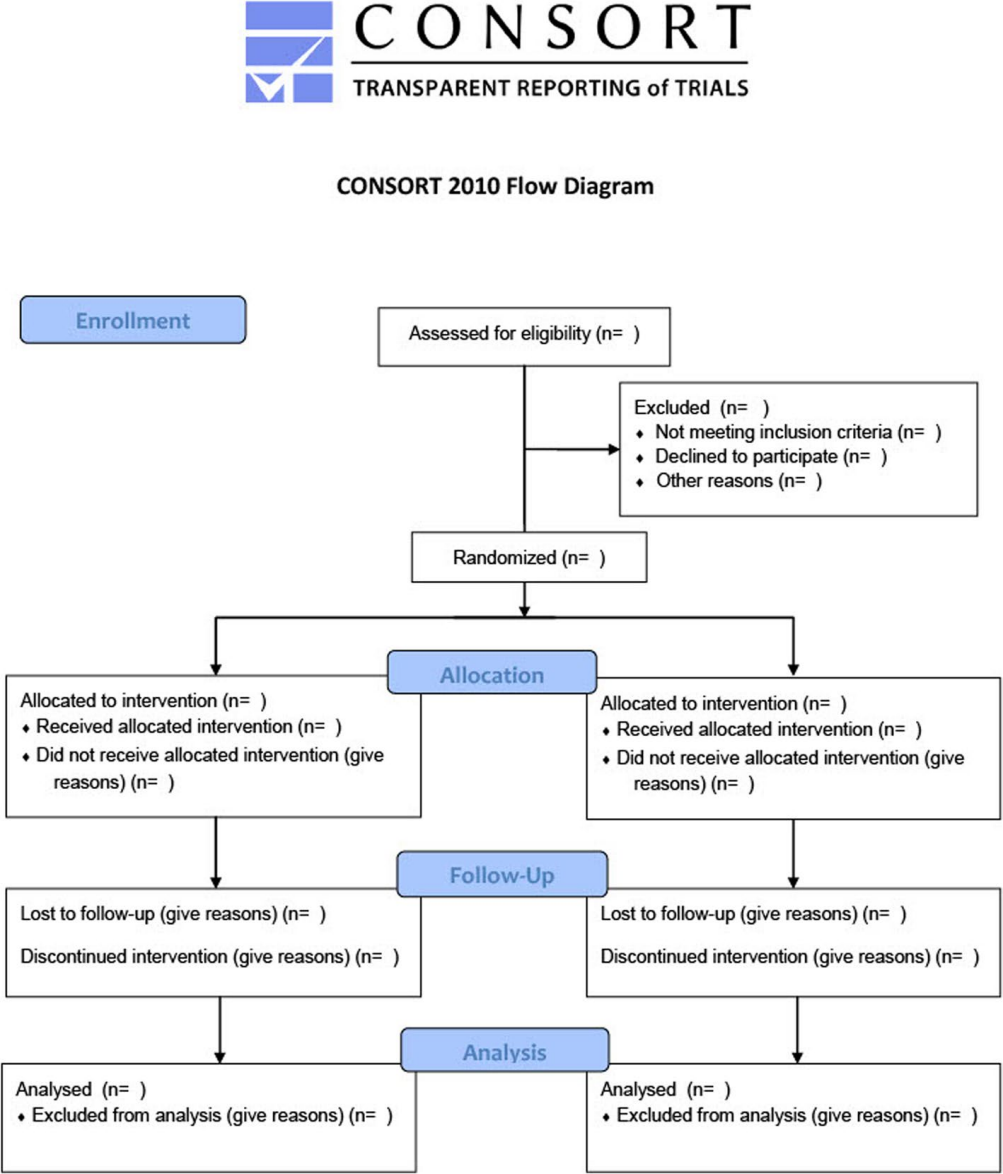
Consolidated Standards of Reporting Trials diagram

Flow of patients through the study will be displayed in a standard Consolidated Standards of Reporting Trials diagram (Fig. 2). The report will include the number of patients included, withdrawn, lost to follow-up, the number who received the allocated treatment, and the number of patients analyzed.

#### Patient characteristics and baseline comparisons

To assess balance, description of collected baseline characteristics (Supplementary Material 1) will be presented for the experimental (tirofiban) and control (aspirin) groups. Discrete variables will be summarized as frequencies and percentages. Unless otherwise indicated, percentages will be calculated according to the number of patients for whom data are available. If there are more than 5% missing values for a variable, the denominator will be added as a footnote in the corresponding summary table. Continuous variables will be summarized using either mean and standard deviation or median and interquartile range (IQR). Time intervals will be summarized by medians and IQRs. Adjudication and definitions of clinical outcomes will be described in the Supplementary Materials.

Primary outcomes:

An outcome definition that includes five elements have been employed: (1) the *domain* or outcome title, (2) the *specific measurement* or technique/instrument used to make the measurement, (3) the *specific metric* or format of the outcome data from each participant that will be used for analysis, (4) the *method of aggregation* or how data from each group will be summarized, and (5) the *time-points* that will be used for analysis [24]:

• Efficacy outcome:

1. (Domain) Primary efficacy outcome: Radiological outcome
2. (Specific measurement) Acute internal carotid artery stent thrombosis: The outcome is measured using doppler ultrasonography. In-stent reocclusion or thrombosis: which will be defined by the presence at the level of the occlusion point, by a characteristic biphasic, brief, and low-velocity pattern both in Doppler spectrum and in color mode (color image with both orthodromic and antidromic flow, red-blue just proximal to the occlusion). In addition, the image detected in B mode will show an anechoic appearance with a false appearance of permeable light, detecting the absence of flow in color and Doppler mode. In the presence of severe stenosis or occlusion on Doppler, angioCT must be performed to confirm the Doppler findings
3. (Specific metric) Stent thrombosis will be assessed by measuring:
  Differences in proportions of stent thrombosis between treatment groups. This outcome will be measured on a binary scale, defined as positive in the presence of events and negative otherwise
  Comparison of the difference in risk of thrombosis between groups: Differences in proportions of stent thrombosis between treatment groups. This outcome will be measured on a binary scale, defined as positive in the presence of events and negative otherwise
  Comparison of the difference in risk of thrombosis between groups
    Statistical hypotheses: For each separate outcome, the set of statistical hypotheses are *p* (tirofiban) = *p* (control) versus *p* (IV tirofiban) ≠ *p* (control), where *p* (tirofiban) is the proportion of subjects with the specific clinical outcome in the tirofiban group and *p* (control) is the proportion with the specific clinical outcome in the control group
    Analysis method:
      ❖ An unconditional logistic regression model will be fitted for each outcome separately to estimate the OR associated with treatment effect, restricting adjustment to age, baseline NIHSS score, and intravenous fibrinolysis. Corresponding 95% CIs will be provided. Unadjusted analyses will also be presented for all secondary efficacy and safety outcomes. The differences in the key prognostic factors will be analyzed continuously in the numerical variables (age, NIHSS) and by percentages in the case of the use of intravenous fibrinolysis. Likewise, differences in the randomization groups will be assessed according to the following coding. Age will be coded as < 80 years and ≥ 80 years. The baseline NIHSS will be classified into two subgroups: low NIHSS (≤ 6) and NIHSS > 6. Regarding IV fibrinolysis, it will be classified based on whether or not it was administered. A separate set of analyses will be performed stratified by patients who received tirofiban or aspirin treatment. If OR = 1, the treatment effect is equal in both groups
      ❖ In addition, treatment effects will be calculated as absolute increase in probability for carotid reocclusion outcome/absolute risk reduction (ARR) in percentage points and with number needed to treat (NTT) defined as 1/ARR. ARR = the absolute risk (AR) of events in the control group (ARc) − the AR of events in the treatment group (ARt).
      The method used to estimate and report the risk difference will be through the odds ratio (OR) in the context of a logistic regression model. The formula for the OR is = \frac {*p* (tirofiban)}{1 − *p* (tirofiban)}}{*p* (control)}{1 − *p* (control)}}, where *p* (tirofiban) and *p* (control) are the estimated proportions of stent thrombosis in the respective groups. After adjusting by key prognostic factors in the logistic regression model, the adjusted risk difference will be calculated
4. (Method of aggregation): Proportion (%) of events and difference in log odds among treatment groups (OR)
5. (Time point): This outcome measure will occur during the endovascular treatment (ultra-early occurrence, T0, visit 2) and within the first 24 h after stent placement (acute occurrence, T24, visit 3).

• Safety outcome:

1. (Domain) Primary safety outcome: Radiological outcome
2. (Specific measurement): Symptomatic intracranial hemorrhage on brain-CT or brain-MRI. The outcome is measured using non-contrast computed tomography (NCCT) or magnetic resonance image (MRI). Definition employed for sICH is the one described by the European-Australian Cooperative Acute Stroke Study 3 (ECASS-III) [25]. Such definition describes any intracranial hemorrhage associated with clinical deterioration defined by an increase of ≥ 4 points in NIHSS score or that led to death assessed on CT or MRI within 36 h after stroke onset
3. (Specific metric)
  Differences in proportions of symptomatic intracranial hemorrhage between treatment groups. This outcome will be measured on a binary scale, defined as positive in the presence of events and negative otherwise
  Comparison of the difference in risk of thrombosis between groups:
    - Main analysis: Common OR from an ordinal logistic regression model adjusted for age, stroke severity (baseline NIHSS score), and intravenous fibrinolysis will be used if the proportional odds assumptions are satisfied (approximate likelihood-ratio test of proportionality of odds are not significant). However, if the proportional odds assumptions are not satisfied, the assumption-free Wilcoxon–Mann–Whitney generalized odds ratios (WMW GenOR) will be used [26]
    - Statistical hypotheses: The null hypothesis which is to be refuted in the ordinal logistic regression is that the common OR is equal to 1, i.e., there is no difference in treatment effect between the intervention and control groups. The null hypothesis to be refuted in WMW GenOR test is equality of ranks when ties are split evenly. The null hypothesis for the WMW GenOR test states that the probability that the treatment observation is better than the control observation is the same as the probability that the treatment observation is worse than the control observation (splitting the ties equally), i.e., the WMW GenOR is equal to 1
    - Analysis of the primary outcome. If the analyses of the baseline characteristics of the trial patients show clear differences in key prognostic factors (age, stroke severity, and intravenous fibrinolysis) between treatment groups, this may complicate the estimation of the treatment effect. The primary analysis of the effect of treatment on the primary outcome will therefore be adjusted for the following covariates: age, symptom severity (baseline NIHSS score), and intravenous fibrinolysis. An unadjusted analysis will also be presented. The differences in the key prognostic factors will be analyzed continuously in the numerical variables (age, NIHSS) and by percentages in the case of the use of intravenous fibrinolysis. Likewise, differences in the randomization groups will be assessed according to the following coding. Age will be coded as < 80 years and ≥ 80 years. The baseline NIHSS will be classified into two subgroups: low NIHSS (≤ 6) and NIHSS > 6. Regarding IV fibrinolysis, it will be classified based on whether or not it was administered. A separate set of analyses will be performed stratified for patients who received experimental and control treatment
    - In addition, treatment effects will be calculated as absolute increase in probability for symptomatic intracranial hemorrhage outcome/absolute risk reduction (ARR) in percentage points and with number needed to harm (NNH) defined as 1/(ARt − ARc). ARt = absolute risk treatment group. ARc = Absolute risk in control group
    Secondary efficacy and safety outcomes and additional outcomes will be displayed in Supplementary Material 1.
4. (Method of aggregation): Proportion (%) of events and difference in log odds among treatment groups (OR)
5. (Time point): This outcome measure will occur within the first 36 h after randomization between treatment groups (T36, visit 3).

#### Sensitivity analysis

Sensitivity analyses for the primary outcome will be performed to test for the robustness of the primary analysis with regard to protocol violations, baseline imbalance, clustering effects, and missing data as outlined in the following. All individuals are included in sensitivity analyses.

Adherence to the intervention will be reported. To account for effects of any off-protocol interventions, the results will also be reported for the “per protocol population” who received tirofiban compared with the control group (aspirin) “per protocol.” For safety outcomes, sensitivity analysis will be performed and reported for the “safety population” in which a patient is included if, and only if, they actually received a study treatment. Due to the possibility of crossovers between groups, its rate (%) will be established, including its impact on the primary safety and efficacy endpoints, through per-protocol analysis.

If analyses of the baseline characteristics of the patients in the trial show clear differences in key prognostic factors (age, stroke severity, and intravenous fibrinolysis) between treatment groups, this may complicate the estimation of the effect of treatment. Both baseline covariate-adjusted and unadjusted results will be reported; however, adjusted analysis is pre-specified as the primary analysis for this RCT.

Sensitivity analyses with regard to clustering effects will be conducted by calculating *p*-values and CIs for the treatment effect on the primary outcome after adjustment for (i) center (taken as a random effect) and (ii) region. These analyses will be performed using mixed effect ordered logistic regression models, and treatment effects will be expressed as adjusted common ORs. In the case that centers with significant data quality issues are identified, further sensitivity analyses will be run to assess whether adjustment for these factors affects the primary outcome.

#### Protocol deviations

The nature and reasons for the protocol violation shall be recorded in the eCRF, in the source documents, and in the monitoring visit report. All such serious non-compliance will be followed up and reported per local regulations. In parallel, corrective and/or preventive actions will be undertaken and documented, including any retraining of the investigator and site staff. All patients who have been included in the trial will be followed up, irrespective of whether treatment was discontinued prematurely or whether the protocol was violated.

*Protocol deviations in consent procedure.

These will be tabulated and accompanied by a brief textual description.

*Protocol deviations at infusion.

It is possible that some patients allocated to tirofiban will not receive their allocated treatment or receive incorrect dose, and some of those allocated control will receive tirofiban. These deviations will be tabulated. Patients will remain in their allocated treatment group for analysis, irrespective of treatment received. These protocol deviations will be analyzed in the per-protocol analysis.

Security assessment:

- Adverse events (AA) of interest for follow-up

Clinical follow-up and detection of adverse events will be carried out systematically at 24–36 h, at discharge, and 3 months after follow-up. The low-dose regimen of tirofiban has been previously used in several clinical studies of patients with ischemic stroke secondary to tandem lesion, showing sufficient safety data to consider its use in a clinical trial. However, the major complication to be taken into account in the experimental group is the increase in the rate of intracranial hemorrhage, whether symptomatic or not. Therefore, a CT scan of the skull will be performed on all patients 24 h after the start of the procedure, before there is clinical neurological worsening of the patient.

Adverse effects arising from the catheterization approach are possible: inguinal hematoma requiring transfusion. These effects derived from the approach route represent a rare complication whose incidence is less than 1%. The evolution of arterial access will be monitored for the diagnosis and early treatment of a femoral pseudoaneurysm as well as perforation or laceration of any arterial branch related to arterial access.

The following are reasons for subjects to discontinue to study: any adverse event that, at the discretion of the clinician, requires the withdrawal of the study medication; when, for any reason, the treatment is no longer safe for the patient; or for any other reason that may endanger the life of the patient or have serious consequences for the same.

Subjects who have discontinued the study as a result of adverse events will receive appropriate alternative treatment, and the investigator should record the reason for discontinuation of the study, facilitate or schedule appropriate follow-up (if necessary) of these subjects, and document the evolution of the subject’s condition. All medications administered up to the time of discontinuation must also be recorded in the concomitant medication section of the data collection booklet.

##### Reporting and collection of serious adverse events

The principal investigator or a collaborator shall report to the pharmacovigilance department (PD) PD-UICEC-HUVR all serious adverse events (as defined in the following), whether or not considered treatment-related, expected or not, within 24 h (one working day), of his knowledge. Serious adverse events occurring at any time after the patient’s inclusion in the study (defined as the time when the subject’s participation in the study is consented to) and up to 30 days after the subject concludes or leaves the study. A subject is considered complete EITHER after the conclusion of the last visit or contact (e.g., telephone contact with the investigator or a collaborator), as indicated in the protocol evaluation schedule, OR after the last dose of study medication, whichever is later. Withdrawal is defined as the date on which a subject and/or the investigator determines that the subject can no longer meet the study requirements at any subsequent visits and evaluations.

The investigator shall complete and sign the AAG notification form which it will be sent to the pharmacovigilance office of the Clinical Research and Clinical Trials Unit in the Virgen del Rocío University Hospital.

PD staff will review the form received and, if appropriate, request additional information from the investigator. The investigator shall provide information to the sponsor or to whoever assumes the tasks delegated by the sponsor (PD-UICEC-HUVR Unit) whenever requested and in any case when his initial assessment changes as to severity or causality.

##### Exceptions to the collection of standard AA

When there is a deterioration of the disease under study, uncertainty may arise as to whether it is lack of efficacy of the test medication or progression of the disease or constitutes an AA. In these cases, unless the sponsor or the notifying physician considers that the study treatment contributed to the deterioration of the disease or local regulations state otherwise, such deterioration shall not be considered as AA but as loss of efficacy or progression of the disease if they meet the following definitions:

- Loss of efficacy: Insufficient therapeutic effect collected as a result of efficacy. Discontinuation due to insufficient therapeutic effect (i.e., lack of efficacy) should not be listed as AA. A clinical failure should not be recorded as AA
- Disease progression: The progression of the disease can be considered as a worsening of the subject’s condition attributable to the disease for which the different treatments of disease are being studied

This worsening may consist of an increase in the severity of the disease under study and/or an increase in the symptoms of the disease. If it is an expected progression, unless it is more severe in intensity or more frequent than expected for the study condition treated in the trial, it should not be recorded as AA.

Any event or hospitalization that is prolonged due to disease progression should not be recorded as a SAA, unless it is believed that the study drug has actively contributed to the progression of the disease (insufficient therapeutic effect is not considered here).

In this sense, AA/AAG derived from or related to the reocclusion or restenosis of the stent will not be notified (these will be collected for evaluation as study variables in the data collection notebook) and with the progression of the study disease, events that due to their expected within the evolution of the pathology under study will not require specific notification to the pharmacovigilance department (it will not be necessary to complete the PD form). SAES). The most common ones are the following: symptomatic or asymptomatic cerebral hemorrhage; respiratory infection, urinary tract infection, phlebitis; seizure; pulmonary thromboembolism; hyperperfusion syndrome; acute myocardial infarction.

Events that are unequivocally associated with disease progression should not be reported as AA/AAG during the active study period, unless the outcome is fatal. It will be the cause that causes the death of the patient that is registered as AAG in the data collection notebook and notified by means of AAG form to PD-UICEC-HUVR within 24 h of its knowledge.

##### Security analysis

The number and percentage of patients dropping out of the study due to adverse events, patients who have experienced at least one adverse event, the most common adverse events, and patients who have experienced at least one serious adverse event shall be calculated. The 95% confidence interval will also be calculated. In addition, an independent data safety and monitoring board (IDSMB) will oversee the conduct of the trial. In the randomized, comparative phase, an interim safety evaluation will be conducted by the IDSMB at the time of enrollment of 120 patients. If there are concerns about participant safety, the IDSMB will make a recommendation to the steering committee about continuing, stopping, or modifying the trial. The IDSMB description is expanded in Supplementary Material 2.

## Trial status

Recruitment for the ATILA clinical trial started in March 2022 under protocol version 2 of April 2022. There are currently 80 patients recruited, which represents 33% of the total expected. To date, there have been no losses to follow-up of patients included in the clinical trial. The recruitment process is expected to end in December 2024.

### Supplementary Information

**Additional file 1: Supplementary Material 1.** Minor Revision. **Supplementary Material 2.** DSMB. **Supplementary Material 3.** Full protocol.

**Additional file 2.** Study Protocol.

**Additional file 3.** Reporting checklist for protocol of a clinical trial.

## Data Availability

The study data will be deposited in the Open Access journal in which the results are published, following the requirements of the International Committee of Medical Journal Editors (ICMJE). The individual data collected in the CRDe will be shared after its anonymization. In addition, the clinical trial protocol, statistical analysis plan, information sheet, and informed consent will be published. These data will be published immediately after the publication of the results. They will be accessible to anyone who wishes to access them and will be available indefinitely.
