# Supplementary material for: Statistical analysis plan for the multicenter, open, randomized controlled clinical trial to assess the efficacy and safety of intravenous tirofiban vs aspirin in acute ischemic stroke due to tandem lesion, undergoing recanalization therapy by endovascular treatment (ATILA trial)
Source: Trials. 2024 Jan 9;25:35. doi: 10.1186/s13063-023-07817-9 (PMC10775524; doi:10.1186/s13063-023-07817-9)
Supplement: Supplementary file 1 — Additional file 1: Supplementary Material 1. Minor Revision. Supplementary Material 2. DSMB. Supplementary Material 3. Full protocol. [file 13063_2023_7817_MOESM1_ESM.zip › Minor Revision Suplementary material 1 R2.docx]

**Statistical analysis plan for the multicenter, open, randomized controlled clinical trial to assess the efficacy and safety of intravenous tirofiban Vs Aspirin in acute ischemic stroke due to tandem lesion, undergoing mechanical thrombectomy(ATILA trial).**

Trial Registration EudraCT Number, 2021-003874-30.

ClinicalTrials.gov, NCT05225961 registered on Feb 7^th^, 2022.

**ATILA TRIAL INCLUSION AND EXCLUSION CRITERIA**

**Inclusion criteria**

- Patients with acute ischemic stroke (AIS) due to tandem lesion (TL) undergoing mechanical thrombectomy (MT) and carotid stent placement during the procedure.
- The locations of intracranial occlusion that may be included are: terminal intracranial carotid artery ("carotid T"), segments M1 and M2 of the middle cerebral artery, segment A1 of the anterior cerebral artery; and posterior cerebral artery with fetal origin.
- ASPECTS (Alberta Stroke Program Early CT Score) ≥6
- Age ≥18 years
- Written informed consent

**Exclusion criteria**

- Age <18 years
- ASPECTS <6
- Radiological findings of bilateral stroke or stroke involving both anterior and posterior circulation.
- Carotid stenosis due to restenosis or carotid stent occlusion
- Patients eligible for carotid puncture.
- Patients under dual antiplatelet therapy at time of randomization.
- Modified Rankin Scale >2
- History of allergy to iodinated contrast media.
- Pregnancy
- Patients with atherosclerotic intracranial occlusion or arterial dissection.
- History of allergy to aspirin or tirofiban
- History of platelet count <100.000 mm^3^ or history of induced thrombocytopenia by glycoprotein IIb/IIIa inhibitors or iv aspirin.
- Concomitant therapy with direct oral anticoagulants 48 hours prior to randomization OR with vitamin-K-antagonist with INR>1.7.
- History of peptic ulcer in the last three months prior to randomization.
- Any medical condition who may lead to high bleeding risk in the opinion of the investigator.

**BASELINE PATIENT CHARACTERISTICS**

Patient baseline characteristics that will be collected in visit 0. All data will be collected in the patient's clinical history for subsequent transfer to the electronic data collection registry (in which the patient affiliation will remain anonymized)

- Demographics : Age , Sex
- Admission vital signs: Heart rate (beats per minute), Respiratory rate (breaths per minute), Blood pressure (mmHg): Systolic and diastolic blood pressure.
- Stroke admission data: Stroke date onset, time of onset of stroke, last time seen well time, cerebral hemisphere affected, time of arrival at the primary center, time of arrival at the comprehensive stroke center.
- Prior medical records: Hipertension, diabetes, dislipidemia, ischemic heart disease, chronic renal failure.
- Prior medication and start date of this medication.
- Laboratory test upon admission: Hemoglobin (g/dL), platelets (10^3^/µL), normalized prothrombin time, international normalized ratio (INR), blood glucose (mg/dl) and pregnancy test if applicable.
- Basal modified rankin scale
- Basal National Institutes of Health Stroke Scale score.
- Description of the most important findings in the electrocardiogram: sinus rhythm, atrial fibrillation, Q waves, ST alterations, left bundle branch block.
- Basal computed tomography (CT) and angio-CT: ASPECTS score and occlusion site.

**ADJUDICATION AND DEFINITIONS OF CLINICAL OUTCOMES**

Primary efficacy outcome will be adjudicated by an independent endpoint adjudication committee, blinded to treatment allocation. For each patient who is reported to have suspected carotid reocclusion on ultrasound doppler, angio-CT must be performed to confirm doppler results. Angio-CT findings will be blindly assessed by the adjudication committee who will review selected data from the event´s day form and other appropriate source data. The adjudication committee will be provided data on the date and time of randomization, the patient's neurological symptoms (NIHSS score) at entry, the date and time of any event and the suspected type of event, and a detailed report of the expert’s opinion of any post-randomization images.

In addition, an independent data safety and monitoring board (IDSMB) will oversee the conduct of the trial. In the randomized, comparative phase, an interim safety evaluation will be conducted by the IDSMB at the time of enrollment of 120 patients. If there are concerns about participant safety, the IDSMB will make a recommendation to the steering committee about continuing, stopping, or modifying the trial. The IDSMB description is expanded in the New Supplementary material 2: ATILA DSMB.

**Death:** If the patient has died during trial participation, death will be classified according to cause:

- Initial stroke
- Recurrent stroke
- Myocardial infarction
- Infection
- Other vascular cause (e.g. systemic emboli)
- Other non-vascular cause (e.g. malignancy, trauma)
- Unknown cause

**Recurrent stroke:**

Neurological deterioration (increase of ≥4 on NIHSS, after exclusion of other causes for neurological deterioration or intracranial hemorrhage) occurring after mechanical thrombectomy of index stroke will be considered as a recurrent stroke. Recurrent stroke will be classified as **ischemic or unknown** (if not documented on imaging).

**Neurological deterioration due to index stroke:**

The definition is an increase of ≥ 4 points in one or more of the NIHSS sub-scores (irrespective of improvement in any of the other NIHSS). When diagnosing neurological deterioration due to index stroke, systemic reasons for deterioration, such as drug-induced hypotension, drug-induced drowsiness, and intercurrent disease should be excluded. No significant hemorrhage should be found on post randomization CT or MR scan.

**Acute myocardial infarction:**

Either one of the following criteria satisfies the diagnosis of myocardial infarction:

Typical rise and gradual fall (troponin) or more rapid rise and fall (CK-MB) of biochemical markers of myocardial necrosis with at least one of the following:

- Ischemic coronary symptoms;
- Development of pathologic Q waves on the ECG;
- ECG changes indicative of ischemia (ST segment elevation or depression);
- or coronary artery intervention (e. g. coronary angioplasty)
- Autopsy pathologic findings of an acute myocardial infarction.

**Major extracranial bleeding:**

Definition of major extracranial bleeding:

Clinically overt bleeding associated with one or more of:

- Transfusion of >2 red cell units of blood
- A decrease in hemoglobin of 20 g/l (=2 g/dl, = 1.24 mmol/l)
- Bleeding into retroperitoneum not related to groin puncture, intraocular space or major joint
- Bleeding leading to permanent treatment cessation

**SECONDARY EFFICACY AND SAFETY OUTCOMES**

An outcome definition that includes five elements have been employed: (1) the *domain* or outcome title; (2) the *specific measurement* or technique/instrument used to make the measurement; (3) the *specific metric* or format of the outcome data from each participant that will be used for analysis; (4) the *method of aggregation* or how data from each group will be summarized; and (5) the *time-points* that will be used for analysis ^(1)^

1. Secondary efficacy and safety outcomes:

- **Rescue therapy:** endovascular rescue therapy in the presence of platelet aggregation phenomena/plaque instability between treatment groups. Aggregation phenomena or plaque instability was defined as an intrastent filling defect due to the formation of a de novo thrombus or the presence of a residual atherosclerotic plaque with a protrusion greater than 2 mm over the lumen of a vessel that slows intracranial circulation ^(2)^. Rescue therapy involves the use of any endovascular mechanical procedure (angioplasty, new stent, etc.) or the employment of intravenous or intra-arterial medications that are used after the detection of platelet aggregation for its resolution. The specific metric and aggregation method of all secondary outcomes will be described generically at the end of this section. The time point of the outcome: This outcome measure will occur during endovascular procedure (T0, visit 2)
- **Functional outcome:** Good functional outcome after endovascular therapy between treatment groups. Outcome is measured using the modified rankin scale (mRS) dichotomized by good (mRS 0-2) versus unfavorable (mRS 3–6) outcome. Good functional outcome is defined by the presence of a mRS between 0-2 (both included). The assessment of this outcome will be carried out by an evaluator blind to the randomization group. Good functional outcome will be appraised at ninety days (+/- seven days) after enrolment. (T90, Visit 6)
- **Functional outcome**: Assessment across the full mRS scale between treatment groups. The outcome is measured using the mRS ordinal scale (range 0–6). Common OR from an ordinal logistic regression model adjusted for age, stroke severity (baseline NIHSS score) and time since last-known normal will be used if the proportional odds assumptions are satisfied (approximate likelihood-ratio test of proportionality of odds are not significant). However, if the proportional odds assumptions are not satisfied, the assumption-free Wilcoxon–Mann–Whitney Generalized Odds Ratios (WMW GenOR) will be used. This functional outcome will be appraised at ninety days (+/- seven days) after enrolment. (T90, Visit 6)
- **Functional outcome**: Excellent functional outcome (mRS 0–1) at 3 months poststroke between treatment groups. Outcome is measured using mRS dichotomized by excellent functional outcome (mRS 0-1) versus unfavorable (mRS 2–6) outcome. This functional outcome will be appraised at ninety days (+/- seven days) after enrolment. (T90, Visit 6)
- **Functional outcome**. Poor outcome defined as mRS score dichotomized by poor outcome (mRS 4-6) versus mRS 0–3 at 3 months between treatment groups. This functional outcome will be appraised at ninety days (+/- seven days) after enrolment. (T90, Visit 6)
- **Brain Computed tomography (CT) assessment**: Assessed between treatment groups
- Parenchymal hemorrhage type 2 ^(3)^ according to Heidelberg classification scheme.
- Any intracranial hemorrhage detected on follow-up imaging at 36 h.
- Parenchymal hemorrhage type 2 ^(4)^ according to ECASS-II classification on follow-up imaging at 36 h.

All of these brain CT assessments will occur within the first 36 hours after randomization between treatment groups (T36, Visit 3).

- **Systemic bleeding**: Incidence of major bleeding defined by ISTH ^(5)^ for tirofiban vs aspirin group. The measurement of this outcome will occur during the duration of the entire trial.
- **Radiological assessment**: Stent reocclusion or significant carotid restenosis (≥ 70%) at 30 days after stent placement. This outcome will be evaluated using ultrasound. Significant carotid restenosis (≥ 70%) is defined as a peak systolic velocity (PSV) ≥ 300 cm/s. Stent reocclusion definition employed is the presence at the level of the occlusion point, by a characteristic biphasic, brief, and low-velocity pattern both in Doppler spectrum and in color mode (color image with both orthodromic and antidromic flow, red-blue just proximal to the occlusion). In addition, the image detected in B mode will show an anechoic appearance with a false appearance of permeable light, detecting the absence of flow in color and Doppler mode. In the presence of severe stenosis or occlusion on Doppler, angio-CT will be performed to confirm the Doppler findings. This outcome will be assessed at day 30+/-7 after patient enrollment (T30, V5).

** Generic Specific metric for previous outcomes: These outcomes are each measured on a binary scale and are defined as positive in the presence of events and negative otherwise. Every outcome will be assessed by measuring differences in proportions between treatment groups.

- Statistical hypotheses: For each separate outcome, the set of statistical hypotheses are p (Tirofiban) = p (control) versus p (IV Tirofiban) ≠ p (control), where p (Tirofiban) is the proportion of subjects with the specific clinical outcome in the Tirofiban group and p (control) is the proportion with the specific clinical outcome in the control group. If OR=1, the treatment effect is equal in both groups.
- Analysis method: An unconditional logistic regression model will be fitted for each outcome separately to estimate the OR associated with treatment effect, restricting adjustment to age, baseline NIHSS score, and intravenous fibrinolysis. Corresponding 95% CIs will be provided. Unadjusted analyses will also be presented for all secondary efficacy and safety outcomes. A separate set of analyses will be performed stratified by patients who received tirofiban or aspirin treatment.
- **Mortality:** Differences in 3 months mortality between treatment groups. Event is defined as death of any cause within 3 months post-stroke. In addition to determining mortality as differences in proportions between treatment groups, this secondary safety outcome will be measured as time-to-event data.
- Statistical hypothesis: The set of statistical hypotheses is hazard (IV Tirofiban) = hazard (control) versus hazard (IV Tirofiban) ≠ hazard (control). If HR=1, the hazard of death is equal in both groups.
- Analysis method: The effect of treatment allocation on survival will be assessed using rate of mortality and time-to-event analysis (defined by days) by Cox proportional hazards models adjusted for age, baseline NIHSS score and intravenous fibrinolysis. The risk related to treatment will be presented as HRs with the corresponding 95% CI and corresponding survival plots will be presented. Proportional hazard assumption will be tested on the basis of Schoenfeld residuals. If the proportional hazard assumption is not met, survival time will be split in three intervals of 30 days and a hazard ratio will be estimated for each interval using time-dependent Cox model. Unadjusted Kaplan-Meier survival curves with the log-rank test will also be presented. The aggregation method will be proportion of evet among treatment groups and risk difference between groups (Hazard ratio)

A separate set of analyses will be performed stratified for patients who received Tirofiban or Aspirin.

Exploratory analyses for additional outcomes, planned subgroups and meta-analyses are included in corresponding epigraphs.

An interim analysis will be carried out, when the inclusion has reached 120 patients (60 from each group) to assess the safety of both therapies under study.

1. Additional Outcomes:

- **Clinical outcomes:**
- NIHSS score at 24 (± 6) hours and discharge day.
- Change in NIHSS score from baseline to 24 (± 6) hours and from baseline to discharge day.
- Proportion of patients with NIHSS score reduction > 8 points or reaching 0 to 1 at 24 (± 6) hours and at discharge day.
- Proportion of patients with
- **Stroke progression,** defined as an increase of ≥2 points according to the National Institutes of Health Stroke Scale (NIHSS) within 48 or 72 h of stroke onset (6,7)
- Recurrent ischemic stroke
- Major extracranial bleeding defined by ISTH
- Myocardial infarction
- **Radiological outcomes:**
- Infarct volume measured on CT at 24 (± 6) hours
- Alberta Stroke Program Early Computed Tomography (ASPECT) score at 24 (± 6) hours
- Change in ASPECT score at 24 (± 6) hours (absolute and relative to baseline)
- Recanalization according to mTICI 2b/3 differences among treatment groups (Tirofiban and Aspirin).
- **Use of health care system resources:**
- Length of hospital stay
- Re-hospitalization during first 3 months

1. Additional outcomes analysis:

- Clinical Outcomes

Planned exploratory analyses

- NIHSS score at 24 (± 6) hours and discharge day (Absolute and change from baseline to follow-up and NIHSS score reduction > 8 points or reaching 0 to 1). As appropriate, analysis of covariance and unconditional logistic regression models will be fitted to estimate the beta coefficient and OR, respectively, associated with treatment effect on NIHSS score. Death will be awarded the worst score of 42. Treatment group is an independent variable and NIHSS score reduction of >8 points or reaching 0 to 1 at 24 (± 6) hours or discharge day (dichotomized) or change in NIHSS score (range from 0 (normal) to 42 (most severe)) is the dependent variable, including age, fibrinolysis use and side of infarct as a covariate for adjustment purposes. In analysis of covariance, NIHSS score at follow-up is the dependent variable and baseline NIHSS is included as a covariate for adjustment purposes.
- ASPECT score at 24 (± 6) hours will be examined using multivariable ordinal logistic regression. Image modality (CT) and baseline ASPECT score will be included as a covariate for adjustment purposes as appropriate.
- Radiological Outcomes

All patients will undergo NCCT and CT angiography (CTA) before randomization, and NCCT again at 24±6 hours post randomization. CTA or ultrasound doppler at 24±6 hours will be performed to assess stent thrombosis. CT perfusion (CTP) is sometimes performed prior to randomization at centers where these imaging modalities are available and as indicated by local investigators. In addition, a repeat NCCT scan is required if the patient deteriorates neurologically or intracranial hemorrhage is suspected for any reason. Although NCCT scanning is the preferred examination for 24±6 hours follow-up, MRI brain imaging is allowed. All scans are transferred to an electronic format (CD) and sent to the Trial Coordinating Centre at the Virgen del Rocio University Hospital. Images will be assessed with all original identifiers stripped from the record, and then viewed via a secure web-based image viewing system by the panel of expert radiologists at the Virgen del Rocio university Hospital under the leadership of the neuroradiologist Dr. Rafael Ocete. All assessments are made blind to all patient details and treatment allocation. Analyses of the imaging data aim to evaluate the influence on risks and benefits of tirofiban related to acute ischemic lesion extent (NCCT and ASPECT score, CTP penumbra and infarct core) and background brain features (leukoaraiosis, prior lacunes and cortical infarcts) in the context of multivariable prediction models incorporating key clinical variables. Particular interest will focus on the effect of baseline findings suggestive of penumbra on CTP or presence of DWI/FLAIR mismatch on baseline MRI, to predict benefit of tirofiban in patients in the subgroup where MRI or CTP assessments at admission are available.

- Use of health care system resources
- Length of hospital stay
- Re-hospitalization during first 3 months

1. Pre-specified subgroups

Planned subgroup analyses are listed below. These subgroups were selected based on prior local experience ^(2,8)^ and after review of factors that are predictors of prognosis, and for which there is prior evidence that they are potentially important effect modifiers ^(9–17)^. All groups will be studied for interaction with the effect of Tirofiban on the primary outcome and each of the prespecified secondary outcomes, controlling for imbalances in baseline characteristics. The 2-way interactions between treatment groups (Tirofiban or control) and the predefined demographic, clinical and imaging variables on the primary outcome will be explored through multivariable ordinal logistic regression for the primary outcome. For each treatment-by-subgroup interaction a likelihood ratio test will be used with appropriate degrees of freedom.

If appropriate, additional analyses of subgroups will be performed. This will generate a number of exploratory analyses. The interpretation will depend on the p-value for interaction, and the size and confidence limits for the effects in the subgroups being compared. Forest plots will be constructed to illustrate subgroup analyses. Similar analyses will be performed for secondary outcomes with the corresponding analytical approach outlined above.

- Planned subgroup analyzes : Pre-specified subgroups
- Age (< 60, 60-79, ≥80 years)
- Sex (male vs women)
- Fibrinolysis use [yes(y)/no(n)]
- Fibrinolysis agent employed (rtPA vs Tenecteplase)
- Baseline stroke severity (NIHSS <8, 8-14 and ≥15)
- Baseline systolic blood pressure (< 140, 140-179 and ≥180),
- Prior smoking
- Prior diabetes mellitus
- Prior hypertension
- Prior aspirin treatment
- Oxfordshire Community Stroke Project classification; total anterior circulation infarct (TACI), partial anterior circulation infarct (PACI), posterior circulation infarct (POCI), or lacunar infarct (LACI) based on their maximum neurological defects)
- Imaging findings
- ASPECTS score (≤7 versus > 7)
- Collateral **Score** ^(18,19)^ **(CT angiography Collateral Score ≤1 versus >1)**
- ASITN/SIR collateral grading scale (Higashida RT) (≤2 versus > 2)
- Infarct hemisphere (right/left)
- Stent cell type (open vs closed cell)
- background brain features (leukoaraiosis evaluated by Fazekas scale according to 0-1 grades vs 2-3 grades and prior vascular lesions)^(20)^.

**PLANS FOR COMMUNICATING IMPORTANT PROTOCOL MODIFICATIONS**

Once the need to modify the protocol has been identified, this change will first be notified to the person responsible for the study at the local clinical trials office. Next, this local office will contact the office of the central clinical trials unit (located at the Virgen del Rocío Hospital) to inform the principal investigator. Once the need for the change has been assessed, the modification or amendment of the protocol will be written by the person responsible for the clinical trials unit of the Virgen del Rocio hospital, to send a copy to the competent ethics committee (in case of a major modification of the protocol). . The Spanish Medicines Agency and the Carlos III Health Institute, which is responsible for financing, will also be informed for review and approval if appropriate. Once the amendment or modification of the protocol is approved, said change will be ratified and signed by the competent ethics committee, the clinical trials unit of the Virgen del Rocio hospital that acts as the central office and finally by the principal investigator.

**REFERENCES**

1. Saldanha IJ, Dickersin K, Wang X, Li T. Outcomes in Cochrane systematic reviews addressing four common eye conditions: an evaluation of completeness and comparability. PLoS One. 2014;9:e109400.

2. Zapata-Arriaza E, de Albóniga-Chindurza A, Ortega-Quintanilla J, Escudero-Martínez I, Moniche F, Medina-Rodríguez M, et al. Clinical Outcomes of Mechanical Thrombectomy in Stroke Tandem Lesions According to Intracranial Occlusion Location. J Stroke. 2021;23:124-7.

3. von Kummer R, Broderick JP, Campbell BCV, Demchuk A, Goyal M, Hill MD, et al. The Heidelberg Bleeding Classification: Classification of Bleeding Events After Ischemic Stroke and Reperfusion Therapy. Stroke. 2015;46:2981-6.

4. Larrue V, von Kummer R R, Müller A, Bluhmki E. Risk factors for severe hemorrhagic transformation in ischemic stroke patients treated with recombinant tissue plasminogen activator: a secondary analysis of the European-Australasian Acute Stroke Study (ECASS II). Stroke. 2001;32:438-41.

5. Rodeghiero F, Tosetto A, Abshire T, Arnold DM, Coller B, James P, et al. ISTH/SSC bleeding assessment tool: a standardized questionnaire and a proposal for a new bleeding score for inherited bleeding disorders. J Thromb Haemost. 2010;8:2063-5.

6. Weimar C, Mieck T, Buchthal J, Ehrenfeld CE, Schmid E, Diener HC, et al. Neurologic worsening during the acute phase of ischemic stroke. Arch Neurol. 2005;62:393-7.

7. Kwan J, Hand P. Early neurological deterioration in acute stroke: clinical characteristics and impact on outcome. QJM. 2006;99:625-33.

8. Zapata-Arriaza E, Medina-Rodriguez M, Ortega-Quintanilla J, De Albóniga-Chindurza A, Ainz-Gómez L, Pardo-Galiana B, et al. Relevance of Carotid Reocclusion in Tandem Lesions. J Atheroscler Thromb. 2023;30:636-48.

9. Goyal M, Menon BK, van Zwam WH, Dippel DWJ, Mitchell PJ, Demchuk AM, et al. Endovascular thrombectomy after large-vessel ischaemic stroke: a meta-analysis of individual patient data from five randomised trials. Lancet. 2016;387:1723-31.

10. Almekhlafi MA, Hill MD, Roos YM, Campbell BCV, Muir KW, Demchuk AM, et al. Stroke Laterality Did Not Modify Outcomes in the HERMES Meta-Analysis of Individual Patient Data of 7 Trials. Stroke. 2019;50:2118-24.

11. Li Q, Abdalkader M, Siegler JE, Yaghi S, Sarraj A, Campbell BCV, et al. Mechanical Thrombectomy for Large Ischemic Stroke: A Systematic Review and Meta-analysis. Neurology. 2023;101:e922-32.

12. Kim BJ, Singh N, Kim H, Menon BK, Almekhlafi M, Ryu WS, et al. Association between blood pressure and endovascular treatment outcomes differs by baseline perfusion and reperfusion status. Sci Rep. 2023;13:13776.

13. Ls R, Bk M, J B, M HP, A D, Cblm M, et al. Imaging features and safety and efficacy of endovascular stroke treatment: a meta-analysis of individual patient-level data. Lancet Neurol. 2018;17:895-904.

14. Wang Y, Wu X, Zhu C, Mossa-Basha M, Malhotra A. Bridging Thrombolysis Achieved Better Outcomes Than Direct Thrombectomy After Large Vessel Occlusion: An Updated Meta-Analysis. Stroke. 2021;52:356-65.

15. Perez-Vega C, Domingo RA, Tripathi S, Ramos-Fresnedo A, Kashyap S, Quinones-Hinojosa A, et al. Influence of glucose levels on clinical outcome after mechanical thrombectomy for large-vessel occlusion: a systematic review and meta-analysis. J Neurointerv Surg. 2022;14(1):neurintsurg-2021-017771.

16. Vagal A, Aviv R, Sucharew H, Reddy M, Hou Q, Michel P, et al. Collateral Clock Is More Important Than Time Clock for Tissue Fate. Stroke. 2018;49:2102-7.

17. Katsanos AH, Safouris A, Sarraj A, Magoufis G, Leker RR, Khatri P, et al. Intravenous Thrombolysis With Tenecteplase in Patients With Large Vessel Occlusions: Systematic Review and Meta-Analysis. Stroke. 2021;52:308-12.

18. Tan JC, Dillon WP, Liu S, Adler F, Smith WS, Wintermark M. Systematic comparison of perfusion-CT and CT-angiography in acute stroke patients. Ann Neurol. j 2007;61:533-43.

19. Tan IYL, Demchuk AM, Hopyan J, Zhang L, Gladstone D, Wong K, et al. CT angiography clot burden score and collateral score: correlation with clinical and radiologic outcomes in acute middle cerebral artery infarct. AJNR Am J Neuroradiol. 2009;30:525-31.

20. Scheltens P, Erkinjunti T, Leys D, Wahlund LO, Inzitari D, del Ser T, et al. White matter changes on CT and MRI: an overview of visual rating scales. European Task Force on Age-Related White Matter Changes. Eur Neurol. 1998;39:80-9.
