# Supplementary material for: Statistical analysis plan for the multicenter, open, randomized controlled clinical trial to assess the efficacy and safety of intravenous tirofiban vs aspirin in acute ischemic stroke due to tandem lesion, undergoing recanalization therapy by endovascular treatment (ATILA trial)
Source: Trials. 2024 Jan 9;25:35. doi: 10.1186/s13063-023-07817-9 (PMC10775524; doi:10.1186/s13063-023-07817-9)
Supplement: Supplementary file 1 — Additional file 1: Supplementary Material 1. Minor Revision. Supplementary Material 2. DSMB. Supplementary Material 3. Full protocol. [file 13063_2023_7817_MOESM1_ESM.zip › New Supplementary material 2 ATILA DSMBR2.docx]

**Data and Safety Monitoring Board (DSMB)**

**Multicenter randomized clinical trial to assess the efficacy and safety of Tirofiban versus intravenous aspirin in patients with acute ischemic stroke secondary to tandem injury, undergoing recanalization therapy through endovascular treatment (ATILA)**

| **Name of Sponsor:** | Fundación Pública Andaluza para la Gestión de la Investigación en Salud de Sevilla (FISEVI).  Clinical and safety responsibilities delegated to CTU-HUVR |
| --- | --- |
| **EudraCT Number:**  **Clinicaltrials.gov ID:** | 2021-003874-30  NCT05225961 |
| **Principal Investigator (PI):** | Dra. Elena Zapata Arriaza  University Hospital Virgen del Rocío  Interventional Neuroradiology Unit / Diagnostic imaging  Neurology Service  Av Manuel Siurot, sn  41013 – Seville |
| **Date of Charter:** | 5 July 2023 |
|  |  |
|  |  |

1. **Table of contents**

[Data and Safety Monitoring Board (DSMB) 1](#_heading=h.30j0zll)

[2](#_heading=h.3znysh7) Introduction 3

[3](#_heading=h.tyjcwt) Independence of the DSMB 4

[4](#_heading=h.3dy6vkm) Responsibilities of the DSMB 4

[4.1](#_heading=h.1t3h5sf) Objectives 4

[4.2](#_heading=h.4d34og8) General Responsibilities 4

[5](#_heading=h.2s8eyo1) Composition of the DSMB 5

[6](#_heading=h.17dp8vu) Composition of Coordination Team of ATILA study (CT) 6

[7](#_heading=h.3rdcrjn) ATILA coordination Team Responsibilities 6

[8](#_heading=h.26in1rg) Meetings of the DSMB 7

[9](#_heading=h.35nkun2) Trial Termination 7

[10](#_heading=h.1ksv4uv) Communication 8

[10.1](#_heading=h.44sinio) DSMB Minutes 8

[10.2](#_heading=h.2jxsxqh) Recommendations 8

[10.3](#_heading=h.3j2qqm3) Completion of DSMB Activities 9

[10.4](#_heading=h.1y810tw) Document Retention 9

[11](#_heading=h.4i7ojhp) References 10

1. **Introduction**

ATILA is a multicenter, prospective, controlled, randomized, open clinical trial with blind evaluator of patients with acute ischemic stroke secondary to tandem lesion, undergoing recanalization therapy by endovascular treatment in Spain.

The aim of this clinical trial is to assess the efficacy and safety of the use of Tirofiban versus aspirin in patients with ischemic stroke secondary to a tandem lesion, by determining the rate of intra-stent re-occlusion in the first 24 hours after endovascular reperfusion treatment, the presence of intra-stent aggregation during the endovascular procedure that requires rescue therapy or leads to stent occlusion in the acute phase of treatment as well as the rate of symptomatic intracranial bleeding during hospitalization. A total of 240 patients will be included during 3 years. They will be randomized in a 1:1 ratio in an intervention group (120 patients receiving a low dose regimen of intravenous Tirofiban) and a control group (120 patients receiving 500 mg of acetylsalicylic acid intravenously). Patients will be followed for 3 months (six visits).

All analyses will be conducted on data from all randomly assigned patients according to the intention to treat (ITT) principle, i.e. patients will be analyzed in the group they were randomized to, no matter what treatment they received, and regardless of whether they deviated from the protocol in any way. In addition, a comparison of treatment groups that includes only those patients who completed the treatment originally allocated, following per-protocol analysis will be conducted too. All outcomes and analyses are prospectively categorized as primary, secondary, or exploratory. An analysis of the missing data will be carried out to assess the percentage of losses in the different moments of the study, and to identify if mentioned loss has randomly produced.

Differences in all outcomes between the two treatment groups will be tested independently at the two-tailed 0.05 level of significance. All estimates of treatment effects will be presented with 95% confidence intervals (CIs). No formal adjustments will be undertaken to reduce the overall type I error associated with both secondary and exploratory analyses including the subgroup analyses. Their purpose is to supplement evidence from the primary analysis to better characterize the treatment effect.

- Primary efficacy outcome refers to the differences in acute stent thrombosis within the first 24 hours after stent placement between treatment groups. Primary safety outcome will be the differences in symptomatic intracranial hemorrhage within the first 36 hours after randomization between treatment groups.
- Secondary efficacy and safety outcomes:
- Differences in proportions of endovascular rescue therapy in the presence of platelet aggregation phenomena/plaque instability or ultra-acute intra-stent thrombosis between treatment groups. Aggregation phenomena or plaque instability was defined as an intra-stent filling defect due to the formation of a de novo thrombus or the presence of a residual atherosclerotic plaque with a protrusion greater than 2 mm over the lumen of a vessel that slows intracranial circulation3.
- Good functional outcome (mRS 0–2) at 3 months post-stroke: Outcome is measured using the mRS dichotomized by good (mRS 0-2) versus unfavorable (mRS 3–6) outcome.
- Differences in 3 months functional outcome across the full mRS scale between treatment groups Outcome.
- Excellent functional outcome (mRS 0–1) at 3 months post-stroke: Outcome is measured using mRS dichotomized by excellent functional outcome (mRS 0-1) versus unfavorable (mRS 2–6) outcome.
- Parenchymal hemorrhage type 24 according to Heidelberg classification scheme on follow-up imaging at 36 h
- Any intracranial hemorrhage detected on follow-up imaging at 36 h.
- Parenchymal hemorrhage type 25 according to ECASS-II classification on follow-up imaging at 36 h.
- Poor outcome defined as mRS score dichotomized by poor outcome (mRS 4-6) versus mRS 0–3 at 3 months.
- Stent re-occlusion or significant carotid restenosis (≥ 70%) at 30 days after stent placement using ultrasound.
- Differences in 3 months mortality between treatment groups

To calculate the sample size, the approximation of the ARCOSENE was used. The following parameters were used: power 80%, alpha error 5%, estimated difference between two proportions of 22% (for the control group, ASA) and 10% (for the experimental group, tirofiban). With these considerations, and including a 5% loss, the sample size would be equal to 240 patients (120 in each group).

Adverse event (AE) reporting will be in accordance to HMC Human Subjects Research Program (HRP) policies. AE will be defined and graded according to the United States Department of Health and Human Service’s Common Terminology Criteria for Adverse Events (CTCAE v5.0). Participation will be subject to informed consent.

An independent Data and Safety Monitoring Board (DSMB) has been convened to assess the progress of a clinical study, the safety data, and critical efficacy endpoints and provide recommendations to the Coordination **Team of ATILA study (CT)**. The members of the DSMB serve in an individual capacity and provide their expertise and recommendations. The DSMB will review cumulative study data to evaluate safety, study conduct, and scientific validity and data integrity of the study. This Charter will outline the roles and responsibilities and serve as the Standard Operating Procedure (SOP) for the DSMB.

1. **Independence of the DSMB**

Members of the DSMB will not participate as investigators in this study and will not be supervised by study investigators. Members of the DSMB do not have a direct interest in knowing or influencing trial outcome or have a financial or intellectual interest in the outcome of the study under review.

Members of the DSMB will be responsible for notifying the CT of any potential conflict of interests that may arise during the conduct of the trial under review.

1. **Responsibilities of the DSMB**
   1. ***Objectives***

The primary objective of the DSMB is to monitor the safety of the interventions and the validity and integrity of the data from the clinical study. Additionally, the DSMB will evaluate the pace of recruitment and will make recommendations to CT regarding the continuation, modification, or termination of the study.

- 1. ***General Responsibilities***

The general responsibilities of the DSMB are:

- To evaluate, on an ongoing basis, the accumulating safety assessments to ensure the ongoing safety of study subjects.
- To consider factors external to the study when relevant information becomes available, such as scientific or therapeutic developments that may have an impact on the safety of the participants or the ethics of the study.
- To review all documents provided in the DSMB data review packets upon receipt.
- To review the conduct of the study, including protocol violations.
- To review data on participant recruitment, accrual and retention.
- Protect the confidentiality of the study data and the DSMB discussions.
- To make recommendations to continue, modify, or terminate the study.
- Communicate DSMB recommendations with CT and appropriate members of the project team via teleconference.
- Providing a written detailed report with conclusions of each meeting in the deadline proposed.

1. **Composition of the DSMB**

The Committee will be composed of 3 members, who are experts in epidemiology, clinical trial methodology and biomedical statistics.

Members of the DSMB:

- Maria Usero Ruiz. MD. Neurology Department. Hospital General de Ciudad Real. [mariuser@gmail.com](mailto:mariuser@gmail.com)
- Luis San Román Manzanera. MD, PHD, Associate radiologist professor. Interventional Neuroradiology Department. Hospital Clínico deBarcelona. [LROMAN1@clinic.cat](mailto:LROMAN1@clinic.cat)
- Jorge Rodríguez Pardo de Donlebún. MD, PhD. Vascular neurologist, Neurology Department. Hospital Universitario de la Paz, Madrid. [jorge.rpdd@gmail.com](mailto:jorge.rpdd@gmail.com)

A quorum will occur when ALL members are present. Meetings will be organized through virtual connections. All members will serve to the end of the clinical study, projected to be until July 2024 (Last Patient Last Visit).

1. **Composition of Coordination Team of ATILA study (CT)**

The coordination team is composed by the group of decisions in the development of the study:

1. Dra. Elena Zapata Arriaza, MD-PhD, Interventional Neuroradiology Unit / Diagnostic imaging/Neurology Department; Principal Investigator and coordinator for ATILA; University Hospital Virgen del Rocío, Seville
2. Dr Alejandro González MD-PhD, Interventional Neuroradiology Unit / Diagnostic imaging/Neurology Department; Sub-Investigator for ATILA; University Hospital Virgen del Rocío, Seville
3. Dr Francisco Moniche Álvarez MD-PhD, Stroke Neurologist/Neurology Department; Sub-Investigator for ATILA; University Hospital Virgen del Rocío, Seville
4. Dra. Clara M Rosso Fernández, MD-PhD, Clinical Pharmacologist, responsable of CTU-HUVR, delegated from sponsor to perform clinical and safety sponsor tasks for ATILA, University Hospital Virgen del Rocío, Seville.
5. Dra. María de los Ángeles Lobo Acosta, MD, Clinical Pharmacologist, Responsible of Pharmacovigilance at UICEC-HUVR; University Hospital Virgen del Rocío, Seville.
6. Dra. Silvia Jiménez Jorge, PhD, Biologist/Epidemiologist, Project Manager at UICEC-HUVR; University Hospital Virgen del Rocío, Seville.
7. **ATILA coordination Team Responsibilities**

The following activities are the responsibility of CT:

- Approve selection of DSMB members.
- Review and approve DSMB Charter.
- Review and implement the DSMB recommendation(s), as appropriate.
- Advise appropriate individuals of DSMB recommendations, and notify CT for information to regulatory authorities and investigators when required or necessary.
- Review conflict of interest information and take actions, if necessary, based on findings of conflicts.

Aditionally, as data coordinating center responsible for analyzing the study data, the following activities:

- Facilitates regular teleconferences for DSMB.
- Provides DSMB regularly scheduled reports 2 weeks prior to scheduled meetings.
- Provides ad hoc reports requested by the DSMB in a timely manner.
- Provides trial biostatistician to explain reports, if required.

1. **Meetings of the DSMB**

The DSMB meetings are foreseen for the time on the study achieves the patient n=120 included. Data included for DSMB evaluation should have been previously monitored by the assigned CTA and considered clean and closed in the CRD of the study.

The DSMB will conduct the scheduled meetings via teleconference to review recruitment data, adverse events, primary and secondary outcomes and protocol violations.

The attendees for these meetings will include DSMB members, the Principal Investigator and CT members.

At the time points scheduled the CT will provide the DSMB members with the monitored data to be reviewed, this data will be sent at least 15 days before the data meeting is agreed. The data report will be composed with:

- Report elaborated from CT with data on recruitment, protocol deviations and adverse events notified to the pharmacovigilance center for the study summarized and classified in a DSUR (Data safety update report) draft.
- Protocol version approved
- DSMB chart with details of procedures

A brief teleconference will be held between the DSMB Chair and CT representative(s) to discuss the recommendations of the DSMB.

Unscheduled meetings can be requested by any party with the responsibility of overseeing the study. Requests can be made to the DSMB members, PI, or CT.

The DSMB may request special reports on an as needed basis. These requests will be made to the CT, who will ensure that the required data are provided in a timely manner.

1. **Trial Termination**

The ability to recommend study termination is solely within the DSMB’s discretion and judgment. Neither the trial investigators nor any other party will interfere with the DSMB’s decisions, beyond providing any data or clarifications requested by the DSMB.

The DSMB may make a binding recommendation to terminate the study if there is substantial ethical concern as a result of any of the following:

1. Excessive AE in the intervention arm (in terms of frequency and/or severity).

The DSMB will make a judgment based on the frequency and severity of AE observed in the control and the intervention arms of the study. Trial termination can be recommended if it is deemed that continuation may expose participants to unacceptable risk of harm.

1. Unsatisfactory trial conduct or excessive protocol violations.
2. Inefficient enrollment to the point where the usefulness of continuing the trial becomes seriously questionable.
3. Interim analysis providing statistical evidence of futility

Cleaning data for the interim analysis will be provided for consideration to the DSMB members.

1. **Communication**

This DSMB will meet via teleconference at the times scheduled. An agenda will be provided. It is estimated that each teleconference will last for no longer than 60 minutes.

In addition, the CT will report to the DSMB associated serious and unexpected adverse events at the time of scheduled points for the analysis. Additional reports will be provided if requested by the Board. Study status reports will be provided to the DSMB at least two weeks prior to each scheduled meeting.

- 1. **DSMB Minutes**

The DSMB will be requested to send a report of the conclusions of the data revision with the specific recommendations, in case of termination or major modifications, a detailed justification will be asked. In case or major modification and, above all in case of recommendation of termination, an urgent communication to the CT will be asked, by phone contact or email in order to proceed accordingly. Written reports can be sent afterwards.

- 1. **Recommendations**

Following the closed session, a brief teleconference will be held between the DSMB and CTT representative(s) to discuss the recommendations of the DSMB.

A brief summary that describes the individual findings, overall safety assessment, and DSMB recommendations will be agreed and forwarded to CT and PI within 2-4 weeks of the meeting.

The DSMB can recommend that the current study continue without modification, continue with specified modifications, discontinue the study or halt or modify the study until more information is available.

- 1. **Completion of DSMB Activities**

The DSMB will remain active until the end of the study or on receipt of written notification from CT.

- 1. **Document Retention**

The DSMB members will maintain a copy of any relevant correspondence, meeting packets, DSMB reports, and meeting minutes in a secure area prior to the meeting occurrence.

1. **References**
2. Guidelines on Data Monitoring Committees. European Medicines Agency, 2005. (Accessed May 13, 2019, at <https://www.ema.europa.eu/en/documents/scientific-guideline/guideline-data-monitoring-committees_en.pdf>)
3. Common Terminology Criteria for Adverse Events (CTCAE). Version 5.0. U.S. Department of Health and Human Services, 2017. (Accessed May 13, 2019, at <https://ctep.cancer.gov/protocoldevelopment/electronic_applications/docs/CTCAE_v5_Quick_Reference_5x7.pdf>)
4. The following regulatory guidelines make reference to independent Data Monitoring Committees. This guideline should be read in conjunction with:

- ICH Note for Guidance E3 (Structure and Content of Clinical Study Reports)
- ICH Note for Guidance E6 (Good Clinical Practice)
- ICH Note for Guidance E9 (Statistical Principles for Clinical Trials)
- Directive 2001/20/EC relating to the implementation of good clinical practice in the conduct of clinical trials on medicinal products for human use
