# Supplementary material for: Statistical analysis plan for the multicenter, open, randomized controlled clinical trial to assess the efficacy and safety of intravenous tirofiban vs aspirin in acute ischemic stroke due to tandem lesion, undergoing recanalization therapy by endovascular treatment (ATILA trial)
Source: Trials. 2024 Jan 9;25:35. doi: 10.1186/s13063-023-07817-9 (PMC10775524; doi:10.1186/s13063-023-07817-9)
Supplement: Supplementary file 1 — Additional file 1: Supplementary Material 1. Minor Revision. Supplementary Material 2. DSMB. Supplementary Material 3. Full protocol. [file 13063_2023_7817_MOESM1_ESM.zip › Supplementary Material 3_ ATILA CLINICAL TRIAL PROTOCOL R2.docx]

CLINICAL TRIAL PROTOCOL

MULTICENTER RANDOMIZED CLINICAL TRIAL TO ASSESS EFFICACY AND SAFETY OF TIROFIBAN VERSUS INTRAVENOUS ASPIRIN IN PATIENTS WITH ACUTE ISCHAEMIC STROKE SECONDARY TO TANDEM LESION, UNDERGOING RECANALIZATION THERAPY BY ENDOVASCULAR TREATMENT.

**CODE:** ATILA-ictus-2021

**EUDRACT No.:** 2021-003874-30

**VERSION:** 2.0 dated April 2022

**PROMOTER:** Andalusian Public Foundation for the Management of Health Research in Seville (FISEVI).



1/50

# 1.- SUMMARY

# Type of request

Clinical trial with an active ingredient of a pharmaceutical specialty marketed in Spain under conditions of use other than that authorized and defined in the summary of product characteristics to establish the best protocol for mono-antiplatelet therapy in the acute phase of TL (tandem lesions).

# Identification of the promoter

Andalusian Public Foundation for the Management of Health Research in Seville (FISEVI).

CIF: G 41918830

Virgen del Rocío University Hospital. Laboratory building, 6th floor.

Manuel Siurot S/N 41013/Sevilla

# Clinical trial title

Multicenter randomized clinical trial to assess efficacy and safety of tirofiban versus intravenous aspirin in patients with acute ischaemic stroke secondary to tandem lesion, undergoing recanalization therapy by endovascular therapy.

# Protocol code

ATTILA-stroke-2021

# Coordinating researcher

Dr. Elena Zapata Arriaza

Virgen del Rocío University Hospital Interventional Neuroradiology Unit UGC Diagnostic imaging

Servicio Neurología Av Manuel Siurot, sn 41013 - Seville

# Centres where the trial is planned

Multicenter study involving nine Spanish hospitals (see Annex I).

# Clinical Research Ethics Committee(s)

Reference Committee: CEIm provincial of Seville.

# Responsible for monitoring

Clinical Research and Clinical Trials Support Unit, Virgen del Rocío University Hospital

# Experimental treatment and control

Experimental Treatment**:** Low-Dose Regimen of Tirofiban Agrastat ®

Dosage: 50 micrograms/ml solution for infusion EFG; A 250 ml bag contains

12.5 mg tirofiban (Agrastat ®). 1 ml solution for infusion contains 50 micrograms tirofiban.

Tirofiban low-dose regimen: 500 microgram bolus iv of tirofiban to be pumped within 5 minutes. The pump will be programmed at 120 ml / h to pass in 5 minutes, which is equivalent to 10 ml (500 micrograms) of tirofiban.

After 5 minutes the dose is reduced to 200 micrograms / hour. The infusion pump should be programmed at 4 ml/hour for up to 24 hours (total infused dose 96 ml).

Pharmaceutical Form: Solution for infusion. Clear, colourless solution, pH 5,5-6,5 and osmolarity 270-330 mOsmol/kg.

Route of administration: Intravenous

Therapeutic Group: Blood and hematopoietic organs – antithrombotic agents – antithrombotic agents – Platelet aggregation inhibitors excluding heparin

ATC code: B01A C17

Control treatment: Intravenous Acetylsalicylic Acid Dosage: 500 mg single dose

Pharmaceutical Form: solution for injection Each bottle of SOLUTION FOR INJECTION contains 1 gram of D,L-lysine-glycine acetylsalicylate (equivalent to 500 mg acetylsalicylic acid). This solution for injection is accompanied by 5 ml of water for injection.

Route of administration: slow intravenous; infusion (1 vial of IV ASPIRIN® in not more than 250 ml in 0.9% sodium chloride solution, 5% and 10% glucose solution, Ringer's solution or lactated Ringer), or slowly administered through a 3-way key together with infusion of the aforementioned solutions.

Therapeutic Group: N02BA

# Phase of the clinical trial

Phase IV

# Objectives

Main objective: To assess the efficacy and safety of the use of tirofiban versus aspirin in patients with ischemic stroke secondary to tandem lesion, by determining the rate of reocclusion in the first 24 hours as well as determining the rate of symptomatic bleeding defined as any hemorrhagic transformation associated with a worsening of 4 or more points on the NIHSS scale within the first 36 hours post-randomization

Secondary objectives:

- - - Determine the rate of salvage therapy in the presence of intra-stent aggregation phenomena during the procedure
    - Detection of good functional prognosis rates at 90 days in the different subgroups (defined as a modified Rankin scale (mRS) score between 0-2).
    - To improve pathophysiological knowledge of the complications associated with endovascular treatment of tandem lesions (re-occlusion) through the identification of related plasma biomarkers.
    - Assessment of any of the biomarkers identified as a therapeutic target of reocclusion.
    - Reocclusion or significant restenosis at 30 days.

# Design

Phase IV, randomized, controlled, multicenter clinical trial to assess the efficacy and safety of the use of tirofiban against acetylsalicylic acid in patients with ischemic stroke secondary to tandem lesion, undergoing recanalization therapy by endovascular treatment. The evaluator of the clinical and radiological variables during the follow-up will be blind to the randomization group of each patient.

# Study disease or disorder

Acute ischemic stroke of anterior circulation secondary to tandem lesion. Tandem lesion is defined as one in which there is severe stenosis or occlusion of the internal carotid artery associated with intracranial occlusion of a large vessel ("carotid T", anterior cerebral artery, middle cerebral artery).

# Primary endpoint

The primary endpoint of the study will be the rate of stent reocclusion in the acute phase of treatment at 24 hours (efficacy), as well as the proportion of patients with symptomatic intracranial hemorrhage during hospital admission within the first 36 hours (safety).

# Study population and total number of patients

The study population will consist of adult patients (18 years or older) with acute ischemic stroke of the anterior territory secondary to tandem lesion, with indication for thrombectomy treatment and need for cervical stenting. The sample size necessary to achieve the objectives set corresponds to 240 patients (120 per arm).

# Duration of treatment

Acute mono antiplatelet therapy, maximum duration of the experimental arm, 24 hours.

# Timetable and expected completion date

A total duration of the trial of 3 years is expected from the start of the recruitment period. A period of 3 months is included for the presentation of the documentation to the Spanish Agency of Medicines and Health Products (AEMPS) and to the Ethics Committees, as well as the initial training in the participating centers. The patient recruitment period will be 29 months and another 9 months are calculated for the analysis and subsequent dissemination of results.

# 2.- INDEX

[**1.- SUMMARY 2**](#_heading=h.gjdgxs)

- 1. [Type of request 2](#_heading=h.30j0zll)
  2. [Identification of the promoter 2](#_heading=h.1fob9te)
  3. [Clinical trial title 2](#_heading=h.3znysh7)
  4. [Protocol code 2](#_heading=h.2et92p0)
  5. [Coordinating researcher 2](#_heading=h.tyjcwt)
  6. [Centres where the trial is planned 3](#_heading=h.3dy6vkm)
  7. [Clinical Research Ethics Committee(s) 3](#_heading=h.1t3h5sf)
  8. [Responsible for monitoring 3](#_heading=h.4d34og8)
  9. [Experimental treatment and control 3](#_heading=h.2s8eyo1)
  10. [Phase of the clinical trial 4](#_heading=h.17dp8vu)
  11. [Objectives 4](#_heading=h.3rdcrjn)
  12. [Design 4](#_heading=h.26in1rg)
  13. [Study disease or disorder 4](#_heading=h.lnxbz9)
  14. [Primary endpoint 5](#_heading=h.35nkun2)
  15. [Study population and total number of patients 5](#_heading=h.1ksv4uv)
  16. [Duration of treatment 5](#_heading=h.44sinio)
  17. [Timetable and expected completion date 5](#_heading=h.2jxsxqh)

[**2.- INDEX 6**](#_heading=h.z337ya)

[**3.- GENERAL INFORMATION 10**](#_heading=h.3j2qqm3)

- 1. [Identification of the trial 10](#_heading=h.1y810tw)
  2. [Type of clinical trial 10](#_heading=h.4i7ojhp)
  3. [Data relating to the sponsor 10](#_heading=h.2xcytpi)
     1. [Person authorized by the sponsor, medical monitor 10](#_heading=h.1ci93xb)
     2. [Responsible for monitoring 10](#_heading=h.3whwml4)
  4. [Description of the products under study 10](#_heading=h.2bn6wsx)
  5. [Data from the study researchers 12](#_heading=h.qsh70q)
  6. [Laboratories, Medical Department or Related Institutions 12](#_heading=h.3as4poj)

[**4.- JUSTIFICATION 13**](#_heading=h.1pxezwc)

[**5.- HYPOTHESIS AND OBJECTIVES OF THE TRIAL 21**](#_heading=h.49x2ik5)

- 1. [Hypothesis 21](#_heading=h.2p2csry)
  2. [Objectives 21](#_heading=h.147n2zr)

[**6.- TEST DESIGN 22**](#_heading=h.3o7alnk)

- 1. [Trial variables 22](#_heading=h.23ckvvd)
     1. [Primary endpoint 22](#_heading=h.ihv636)
     2. [Secondary variables 22](#_heading=h.32hioqz)
  2. [Design 22](#_heading=h.1hmsyys)
  3. [Randomization procedure 22](#_heading=h.41mghml)
  4. [Masking 23](#_heading=h.2grqrue)
  5. [Trial treatments 23](#_heading=h.vx1227)
  6. [Patient follow-up 23](#_heading=h.3fwokq0)
  7. [Study completion or discontinuation criteria 24](#_heading=h.1v1yuxt)
  8. [Study medication 24](#_heading=h.4f1mdlm)
  9. [Storage and dispensing of medication 24](#_heading=h.2u6wntf)
  10. [End of the trial 25](#_heading=h.19c6y18)

[**7.- SELECTION CRITERIA 25**](#_heading=h.3tbugp1)

- 1. [Inclusion criteria 25](#_heading=h.28h4qwu)
  2. [Exclusion criteria 25](#_heading=h.nmf14n)
  3. [Withdrawal criteria 26](#_heading=h.37m2jsg)
     1. [By security criteria 26](#_heading=h.1mrcu09)
     2. [For non-compliance or violation of the rules contained in the protocol 27](#_heading=h.46r0co2)
     3. [Follow-up of patients withdrawn prematurely 27](#_heading=h.2lwamvv)

[**8.- TREATMENT OF SUBJECTS 27**](#_heading=h.111kx3o)

- 1. [Duration and adjustments of treatment 30](#_heading=h.3l18frh)
  2. [Contraindicated medication 30](#_heading=h.206ipza)
  3. [Concomitant medication 31](#_heading=h.4k668n3)
  4. [Rescue medication 31](#_heading=h.2zbgiuw)
  5. [Calendar of visits and evaluations 31](#_heading=h.1egqt2p)
  6. [Procedures per visit 32](#_heading=h.3ygebqi)
     1. [Inclusion Visit (Visit 1) 32](#_heading=h.2dlolyb)
     2. [Visit 2 (Day 0, Start of treatment) 33](#_heading=h.sqyw64)
     3. [Visit 3 (24-36 hours after administration of antiplatelet drug) 34](#_heading=h.3cqmetx)
     4. [Visit 4 (Follow-up at hospital discharge) 35](#_heading=h.1rvwp1q)
     5. [Visit 5 (Follow-up 30 days post-treatment, ± 7 days) 36](#_heading=h.4bvk7pj)
     6. [Visit 6 (follow-up 90 days post-inclusion ± 7 days). Telephone 36](#_heading=h.2r0uhxc)
     7. [Unscheduled visit 36](#_heading=h.1664s55)

[**9.- ASSESSMENT OF EFFECTIVENESS 36**](#_heading=h.3q5sasy)

- 1. [Primary and secondary efficacy endpoints 36](#_heading=h.25b2l0r)
  2. [Other studies 37](#_heading=h.kgcv8k)

[**10.- SECURITY ASSESSMENT 37**](#_heading=h.34g0dwd)

- 1. [Security primary variable 37](#_heading=h.1jlao46)
  2. [Security Assessments 37](#_heading=h.43ky6rz)
     1. [Physical examination, vital signs 37](#_heading=h.2iq8gzs)
     2. [Laboratory tests 37](#_heading=h.xvir7l)
  3. [Adverse events of interest for follow-up 38](#_heading=h.3hv69ve)
  4. [Definitions of pharmacovigilance 38](#_heading=h.1x0gk37)
  5. [Reference Safety Information 40](#_heading=h.4h042r0)
  6. [Reporting and collection of serious adverse events 40](#_heading=h.2w5ecyt)
     1. [Exceptions to the collection of standard AA 41](#_heading=h.1baon6m)
  7. [RAGI Expedited Notification 42](#_heading=h.3vac5uf)
  8. [Expeditious notification of other relevant safety information 43](#_heading=h.2afmg28)
  9. [Notification to investigators 43](#_heading=h.pkwqa1)
  10. [Medication errors 43](#_heading=h.39kk8xu)

[**11.- STATISTICS 44**](#_heading=h.1opuj5n)

- 1. [Sample size calculation 44](#_heading=h.48pi1tg)
  2. [Statistical analysis 44](#_heading=h.2nusc19)
  3. [Definitions of the study analysis populations 45](#_heading=h.1302m92)

[**12.- ETHICAL ASPECTS 45**](#_heading=h.3mzq4wv)

- 1. [Informed consent 45](#_heading=h.2250f4o)
  2. [Data protection 46](#_heading=h.haapch)
  3. [Responsibilities of study participants 47](#_heading=h.319y80a)
  4. [Monitoring and auditing 47](#_heading=h.1gf8i83)
  5. [Premature termination or suspension of the study 48](#_heading=h.40ew0vw)
  6. [Study documentation 48](#_heading=h.2fk6b3p)

[**13.- FINANCING AND INSURANCE 48**](#_heading=h.upglbi)

- 1. [Financing 48](#_heading=h.3ep43zb)
  2. Insurance [48](#_heading=h.1tuee74)

[**14.- PUBLICATION POLICY 48**](#_heading=h.4du1wux)

**LIST OF ANNEXES**

ANNEX I. List of participating centres

ANNEX II. DATA SHEET Tirofiban (AGRASTAT*0.05 mg/ml solution for infusion) ANNEX III. DATA SHEET Acetylsalicylic Acid Injection (INYESPRIN®)

ANNEX IV. Bibliography

ANNEX V. Declaration of Helsinki of the World Medical Association ANNEX VI. Concomitant medication

ANNEX VII. Patient information sheet and informed consent. ANNEX VIII. Description of the *National Institutes of Health Stroke Scale* (NIHSS) ANNEX IX. Technical description of peripheral venous catheter placement

ANNEX X. Description scale Modified treatment in cerebral ischemia (mTICI) score for intracranial recanalization post-thrombectomy

ANNEX XI. Description of the ASPECTS scale (Alberta Stroke Protocol Programme Early CT Score)

ANNEX XII. Nomenclature of the hemorrhagic transformation of ischemic stroke according to ECASS criteria

ANNEX XIII. Sub-study of Blood Biomarkers

ANNEX XIV. World Health Organization table for toxicity gradation ANNEX XV. Serious Adverse Event Reporting Form

# 3.- GENERAL INFORMATION

# Identification of the trial

Study code: ATILA-ictus-2021 EudraCT: 2021-003874-30

# Type of clinical trial

Multicentre randomised clinical trial to assess efficacy and safety of tirofiban versus intravenous aspirin in patients with acute ischaemic stroke secondary to tandem lesion undergoing recanalisation therapy with endovascular therapy

# Data relating to the sponsor

Andalusian Public Foundation for the Management of Health Research in Seville (FISEVI).

CIF: G 41918830

Virgen del Rocío University Hospital. Laboratory building, 6th floor.

Manuel Siurot S/N 41013 Seville

# Person authorized by the sponsor, medical monitor

Dr. Clara M. Rosso Fernández

Clinical Research and Clinical Trials Unit Virgen del Rocío University Hospital

Manuel Siurot s/n 41013 Sevilla Phone:955313414

# Responsible for monitoring

Clinical Research and Clinical Trials Unit, Virgen del Rocío University Hospital

Hospital Universitario Virgen del Rocío Avda. Manuel Siurot S/N

41013 Seville Phone:955313414

# Description of the products under study

Experimental treatment**:** tirofiban Agrastat ®

Dosage: 50 micrograms/ml solution for infusion EFG; A 250 ml bag contains

12.5 mg tirofiban (Agrastat ®). 1 ml solution for infusion contains 50 micrograms tirofiban.

Tirofiban low-dose regimen: 500 microgram bolus iv of tirofiban to be pumped within 5 minutes. The pump will be programmed at 120 ml / h to pass in 5 minutes, which is equivalent to 10 ml (500 micrograms) of tirofiban.

After 5 minutes the dose is reduced to 200 micrograms / hour. The infusion pump should be programmed at 4 ml/hour for up to 24 hours (total infused dose 96 ml).

Pharmaceutical Form: Solution for infusion. Clear, colourless solution, pH 5,5-6,5 and osmolarity 270-330 mOsmol/kg.

Route of administration: Intravenous

Therapeutic Group: Blood and hematopoietic organs – antithrombotic agents – antithrombotic agents – Platelet aggregation inhibitors excluding heparin

ATC code: B01A C17

Tirofiban is an inhibitor of glycoprotein IIb/IIIa (Tirofiban ®) with a short half-life (1-2 hours), takes effect in 30 minutes and its action lasts between 4-8 hours, without influence of genetic polymorphisms. It acts as an antiplatelet agent to prevent re-thrombosis of the internal carotid artery after the placement of a stent in the acute phase. It is indicated for the prevention of early myocardial infarction in adult patients with acute non-ST-segment elevation coronary syndromes (STS-ACS) whose last episode of chest pain occurred within the last 12 hours and who have changes in electrocardiogram and/or increased cardiac enzymes. Tirofiban should be administered with unfractionated heparin and oral antiplatelet therapy, including acetylsalicylic acid.

More information in Technical sheet (Annex II).

Control treatment: Acetylsalicylic Salicylic Acid, INYESPRIN® Dosage: 500 mg single dose

Pharmaceutical Form: solution for injection Each bottle of SOLUTION FOR INJECTION contains 1 gram of D,L-lysine-glycine acetylsalicylate (equivalent to 500 mg acetylsalicylic acid). This solution for injection is accompanied by 5 ml of water for injection.

Route of administration: slow intravenous; infusion (1 vial of IV ASPIRIN® in not more than 250 ml in 0.9% sodium chloride solution, 5% and 10% glucose solution, Ringer's solution or lactated Ringer), or slowly administered through a 3-way key together with infusion of the aforementioned solutions.

Therapeutic Group: N02BA

ATC codes: A01AD05, B01AC06, N02BA01, N02BA0.

Acetylsalicylic acid belongs to the group of antipyretic and nonsteroidal anti-inflammatory drugs (NSAISDs). The analgesic effect of acetylsalicylic acid

It is performed peripherally because of inhibition of prostaglandin synthesis, which prevents stimulation of pain receptors by bradykinin and other substances. Also, in the relief of pain are possible central effects on the hypothalamus. The antipyretic effect appears to be due to inhibition of prostaglandin synthesis, although the nuclei of the hypothalamus have a significant role in controlling these peripheral mechanisms. Acetylsalicylic acid inhibits the formation of thromboxane A2, by acetylation of platelet cyclooxygenase. This antiplatelet effect is irreversible during the life of platelets. It is indicated for: rheumatic, neuralgic, post-traumatic, post-operative, postpartum, and neoplastic pain, prophylaxis and treatment of thromboembolic disease, and hyperthermia of any etiology.

More information in Technical sheet (Annex III).

# Data from the study researchers

See Annex I

# Laboratories, Medical Department or Related Institutions

Interventional Neuroradiology Unit. UGC Diagnostic imaging Hospital Universitario Virgen del Rocío

Av Manuel Siurot, sn 41013 - Seville

Pharmacy Clinical Management Unit Virgen del Rocío University Hospital Manuel Siurot S/N

41013 Seville

Clinical Research and Clinical Trials Unit, Virgen del Rocío University Hospital

Manuel Siurot S/N 41013 Sevilla Phone:955313414

Fax: 955095338

Institute of Biomedicine of Seville (IBIS) Calle Antonio Maura Montaner, S/N 41013 Seville

Phone: 955 92 30 00

# 4.- JUSTIFICATION

Approximately 20% of patients undergoing endovascular recanalization treatment (EVT) in acute ischemic stroke in the anterior circulation is due to tandem lesions (TL), defined as severe stenosis or ICA occlusion associated with ipsilateral intracranial occlusion (1)bibliography in Annex IV). In general, patients with TL have been associated with a worse functional prognosis compared to isolated intracranial lesion, associating higher rates of disability and death (2). Intravenous fibrinolysis (IVF) alone is less effective in TL, possibly due to the higher thrombus burden and the lower anterograde flow that prevents access to fibrinolytic medication to intracranial occlusion (2). However, we have growing evidence that shows rates of good functional outcome in TL similar to isolated intracranial occlusions, presenting values of symptomatic hemorrhagic transformation comparable between groups (3,4). Currently, IVF-associated EVT is the standard management of recanalization in patients with ischemic stroke and large intracranial vessel occlusion, over and above IVF used alone(5). This indication is based on the results of five clinical trials (6–10). However, the representation of TLs in these trials is poor, possibly due to the greater technical complexity of the procedure, having been excluded from SWIFT PRIME(6) and EXTEND-IA (7). In the rest, the inclusion of this type of patients was 32.3% in the MR-CLEAN(8), 18.6% in the REVASCAT (9), and 17% in the ESCAPE(10). Therefore, we do not have specific clinical trials in this type of lesion and high-quality evidence for the treatment of people with TL undergoing EVT remains limited.

Even so, the meta-analysis HERMES (Highly Effective Reperfusion using Multiple Endovascular devices) (11), showed that the effect of EVT in patients with TL was comparable to that of patients with isolated intracranial occlusions. However, this patient-level meta-analysis included only 122 patients with TL, and although a clear superiority of EVT over standard management was demonstrated, the approach to extracranial lesion was hardly described in the individual trials on which the meta-analysis was based.

Therefore, although standard practice involves revascularization of intracranial occlusion, there is a lack of consensus around the best acute management of extracranial lesion, as well as optimal treatment strategies to maximize the success of recanalization while minimizing treatment-related complications, such as distal cerebral thromboembolic events, stent occlusion and symptomatic intracranial hemorrhage (sICH).

The main dilemma posed by extracranial lesion in the acute phase is the placement of carotid stents, for which the administration of antiplatelet medication is necessary. However, such antiplatelet therapy increases the risk of hemorrhagic transformation and its use in combination with IVF is currently contraindicated until 90 minutes after fibrinolytic administration (Powers WJ 2019). However, failure to place the carotid stent can lead to extracranial artery reocclusion, lengthening the procedure and exposing the patient to the risk of a new intracranial embolism (12).

a. Therapeutic strategy in tandem lesions.

In the case of a patient with an acute ischemic stroke secondary to a tandem lesion, the therapeutic approach involves two possibilities:

● Retrograde option: Cross carotid stenosis whenever possible and perform mechanical thrombectomy for removal of the intracranial thrombus to subsequently place the extracranial stent.

● Anterograde option: First solve carotid stenosis, by performing angioplasty +/- stent and then perform mechanical thrombectomy at the intracranial level.

Both options are commonly used, and appear to have similar rates of good functional prognosis despite differences in intracranial recanalization times (13–15). The use of one or the other depends on each hospital. The limiting point in both options is the use of the stent in the acute phase. Simple angioplasty (not stenting) is associated with higher rates of restenosis in patients with programmatically treated carotid stenosis with scheduled administration of double antiplatelet therapy(15). In the case of patients with TL, in whom angioplasty is performed to cross the stenotic lesion, the use of at least mono-antiplatelet therapy is essential to try to stabilize the atheromatous plaque and avoid early carotid artery reocclusion (15). However, to protect plaque from atheromatous permanently, carotid stent placement is the most effective solution. The advantages of using or not using the acute phase stent are summarized in Table 1(12).

| Advantages Use Stent | Advantages NO use of Stent |
| --- | --- |
| Treatment and resolution of the athero-embolic cause of stroke.  Lower risk of stroke recurrence, by stabilizing the atheromatous plaque with the stent.  Improvement of anterograde cerebral perfusion in case of stent patency, with potential attenuation of infarct progression  Contribution to spontaneous intracranial lysis of the thrombus, due to anterograde restoration of cerebral perfusion. | Lower risk of hemorrhagic transformation, by avoiding the use of double antiplatelet therapy.  Lower risk of iatrogenic reembolism during stent release |

Regarding the clinical advantages of stent use, two of the most extensive meta-analyses recently published (16–18)showed better recanalization rates in the stent group. In the study conducted by the TITAN collaborative group (3), patients in whom stents were used, associated with the use of IVF and antiplatelet therapy, showed better rates of good functional prognosis compared to the simple angioplasty group. In more recent observational studies, the benefit of stent use maintains encouraging data; STRATIS registry where the modified Rankin scale, 0-2 at 90 days, was higher in the stenting group (68.5% versus 42.2%; P = 0.003) (1); TITAN Registration; in which similar results were found in terms of functional prognosis at 90 days with a higher rate of good outcomes in the context of stenting associated with the use of antithrombotic agents compared with thrombectomy alone (57.9% vs. 41.0%; Unadjusted OR = 1.98; CI 95%

= 1.24–3.15; P = 0.004) (19). These results reflect the growing and current evidence of the clinical benefit of the use of acute phase stents in TL. This benefit is maintained in observational studies with heterogeneous application of antithrombotic treatment, but applying real clinical situations, since in most studies patients received fibrinolytic treatment associated with the antiplatelet agent. The advantages of stent placement focus on intracranial improvement of cerebral perfusion, associated with less recurrence of events due to reocclusion of an artery treated with simple angioplasty, which can occur in up to 22% of cases (20).

Thus, the use of stents appears to be effective and yields encouraging results. However, the main concerns associated with such a device are early reocclusion and symptomatic intracranial hemorrhage (sICH), both related to the use of antiplatelet therapy.

Carotid intra-stent reocclusion is related to the antiplatelet protocol used in the acute phase. Rates of up to 22% have been published(20). In considerable percentages of published studies (16–18), the use of fibrinolytic therapy is widespread in patients with TL, which associated with the use of antiplatelet therapy has shown variable values (14-43%) of hemorrhagic transformation (1,20–22). This causes variability in antithrombotic management to stabilize the atheromatous plaque on which we place the stent, triggering an insufficient antiplatelet protocol that causes acute re-occlusion, increasing the risk of recurrence of ischemic events. The rates of re-occlusion described in recent studies reveal two situations to be taken into account;

1. Acute reocclusion can reach up to 22% according to recent studies(20)associated with the presence of residual intra-stent stenosis or the lower use of post-stenting angioplasty. It is also more frequent in case of not using stent vs stent [42% vs 5.4% (22)]; [ 33.3% vs 10.3% (p = 0.127)](18)

2. Such reocclusion is associated with worse functional prognosis at 90 days, higher rates of symptomatic intracranial hemorrhage (20) and worse percentages of neurological recovery (22).

In general, the publication of intra-stent reocclusion rates in the context of acute rescue endovascular therapy is recent and does not show homogeneous values, possibly due to the variability in both the antithrombotic therapy used and the different technical and device approaches used.

To summarize, the following table describes the main recent studies describing data on acute stent reocclusion.

| Author | Year of publication | Antithrombotic tto | Reocclusion rate | sICH Rate |
| --- | --- | --- | --- | --- |
| Mpotsaris A1 | 2017 | Bolus abciximab (0.125mg/kg) + 300 mg ASA iv after procedure | 6% | 5% |
| Bricout N2 | 2018 | 250 mg ASA iv | 6.5% (Stent)  19.6% (Simple angioplasty) | 10.9% |
| Pop R3 | 2019 | Variable  300 mg clopidogrel per SNG+250 mg ASA iv+  heparin iv) or 250 mg ASA iv +/- 300 mg clopidogrel per SNG | 19.1 % | 6.1% |
| Da Ros V4 | 2020 | Heparin iv + 500 mg ASA iv if not previously taking ASA or 300 mg ASA iv or clopidogrel 75 mg if previously taking antiplatelet drugs | 80% double-layer stent  16.9% single layer stent | 27.7% |
| Oysters D5 | 2020 | Bolus eptifibatide followed by infusion | 7% | 3.4% |
| Neuberger U6 | 2020 | ASA 500 mg iv + clopidogrel periprocedure or tirofiban (bolus 0.4 mg/kg/min in 30 min iv + infusion 0.1 mg/kg/min for 12h). Some were receiving IV teparin | Tirofiban 12.2%  DAT* 6.28% | Tirofiban 6.1%  DAT 10.2% |
| Renu A7 | 2020 | Bolus heparin iv + 900 mg ASA pre-stent and 300 mg clopidogrel after procedure | 22% | 14% Reocclusion vs 1% non-reocclusion stent |

*DAT: double anti-aggregation

(1) Mpotsaris A et al. Interv Neuroradiol. 2017 ;23:159-165. (2) Bricout N, et al. Stroke. 2018 ;49:2520-2522. (3) Pop R et al. AJNR Am J Neuroradiol. 2019 ;40:533-539. (4) Da Ros V et al. AJNR Am J Neuroradiol. 2020;41:2088-2093. (5) Osteraas ND et al. J Stroke Cerebrovasc Dis. 2020;29:105021. (6) Neuberger U et al. J Neurointerv Surg. 2020;12:1088-1093. (7) Renú A et al. Atherosclerosis. 2020 ;313:8-13.

Regarding the rates of hemorrhagic transformation and sICH, this complication also seems to be related to the state of carotid stenosis and its possible reocclusion. The TITAN registry described carotid occlusion as an independent predictor of hemorrhagic transformation(17), and the use of one or two antiplatelet agents was associated with lower rates of sICH. It seems that complete recanalization, including carotid permeability, protects against hemorrhagic transformation, without influence of the association FibIV and antiplatelet (16–18).

In light of these data, the use of the stent protects against carotid reocclusion to a greater extent than simple angioplasty, thanks mainly to the use of antiplatelet therapy, which in turn associates a lower risk of sICH. However, this statement is based on retrospective studies that require validation in randomized studies, but represent the starting point for our project.

However, a fundamental question remains to be answered: What is the best antiplatelet protocol in the acute phase of endovascular treatment of TL?

a. Antithrombotic treatment in the acute phase of TL.

The antithrombotic management used in the main published studies (1,16–18) does not show a homogeneous standardization and the safety results described show variable but encouraging data on mortality and sICH. The possibilities include from not administering any antiplatelet agent, mono-antiplatelet with ASA, clopidogrel or glycoprotein IIb-IIa iv inhibitors, or the combination of some of them. The main concern of the use of antithrombotics is their combination with IV fibrinolytic, although based on the studies described above, the use of IVF is widespread and does not associate higher rates of sICH.

● Clopidogrel , which is a thienopyridine, is not the most recommended antiplatelet agent in the context of acute stroke, given the need for oral administration in patients with dysphagia and/or low level of consciousness. In addition, it takes time to take effect (2-8 hours), and associates multiple genetic polymorphisms and considerable percentages of resistance and hyperresponse that make its use inadvisable in AIS with TL (23,24)

● Acetylsalicylic acid (ASA) irreversibly acetylates the enzyme cyclooxygenase-1 preventing the conversion of arachidonic acid to thromboxane A2 (potent promoter of platelet aggregation) in a dose-dependent manner (23,24). It starts its action in 15-30 minutes and its half-life in plasma is approximately 15-20 minutes (23,24). One of the main limitations in aspirin use is the high rate of resistance to the drug (up to 30% in some studies)(23,24). This resistance is due to multiple interindividual factors (use of NSAISDs, diabetes, hyperthyroidism, etc.) difficult to control in the acute phase of endovascular treatment. Aspirin in doses of 500 mg/iv is probably the most widely used. But in the 2019 American Heart Association (AHA) guidelines (5), the use of ASA within 90 minutes of fibrinolytic administration is contraindicated based on the results of the ARTIS study (25). In this study, the effect of 300 mg of IV + fibrinolytic ASA (rtPA) vs rtPA alone without ASA was compared. An increased risk of symptomatic bleeding was observed in the AAS+rtPA group (4.3% vs 1.6%; P=0.04) but no differences in good functional prognosis at 3 months (54% vs 57.2%; P=0.42)). Extrapolating the results of the 2012 ARTIS (patients where the rate of AIS with TL of the study is unknown) to the present may not be accurate. The primary endpoint of the study (mRs at 90 days) showed statistically significant differences and although the rate of symptomatic bleeding was higher, it is in the range of what has been published in studies with rtPA (22).

● Glycoprotein IIb/IIIa inhibitors (tirofiban): Tirofiban is a non-peptide platelet GP IIb/IIIa receptor antagonist (which is the most abundant integrin on the surface of platelets), effectively blocking GP IIb/IIIa receptors with high selectivity by preventing fibrinogen binding to platelets and subsequent platelet aggregation at the site of atherosclerosis (23,24). Intravenous bolus tirofiban of 10 μg/kg followed by 0.10 to 0.15 μg/kg/min has demonstrated an inhibition of platelet aggregation ex vivo by> 90% in 5 min. Platelet aggregation is restored to approximately 50% at 4 h after cessation of continuous infusion and reaches levels close to baseline after 8 h. This dose-dependent blocking effect is rapidly metabolised following cessation of intravenous infusion and involves a short plasma half-life (approximately 2 h) (23,24). The effect of tirofiban is not affected by genetic polymorphisms and so far no significant rates of drug resistance have been published. The 2019 AHA guidelines (5) specify that the efficacy of tirofiban co-administered with rtPA iv is not well established (with recommendation IIb) and should be administered in the context of clinical trials. It has the added advantage of being administered intravenously, and has been used in some centers to prevent local platelet aggregation and early reocclusion (26–28). Likewise, there is already evidence of clinical benefit from the use of tirofiban, even when combined with rtPA without increasing the risk of sICH or mortality (27). The use of low-dose tirofiban (500 micrograms bolus plus infusion of 200 micrograms per hour, for 18 hours) (26) has demonstrated its safety and efficacy in the endovascular treatment of patients with acute ischaemic stroke. Simple antiplatelet therapy with tirofiban in patients with AIS and TL may be a reasonable alternative.

Abciximab is excluded, as it is contraindicated by the latest AHA guidelines(5).

As an initial pilot study (4) at the Virgen del Rocío University Hospital in Seville, patients with ischemic stroke secondary to TL undergoing thrombectomy have been evaluated. Intravenous (iv) fibrinolysis (rtPA) was administered to elective patients according to the local protocol. All patients were given IV ASA during the procedure and always before carotid angioplasty. Dual antiplatelet therapy with oral clopidogrel or intra-arterial abciximab during the procedure was performed according to the decision of the responsible neurologist and interventional neuroradiologist. Among 784 patients with anterior circulating ischemic stroke included (from January 2017 to January 2019), 22.1% (N = 174) had TL. Fibrinolytic therapy was used in 50.5% of TLs. The rate of successful recanalization (TICI2b-3), good functional prognosis at 3 months (modified Rankin scale 0-2), symptomatic intracranial hemorrhage and death were 89.5% (p=0.32), 57.9% (p=0.80), 5.7% (p=0.20= and 10.3% (p=0.55) respectively. The use of cervical stents was performed in 75 patients (43.1%). All patients in whom stents were used received IV aspirin, in 2 of them the taking of oral clopidogrel (loading dose) was also associated, in two others in addition to aspirin abciximab was used, and in 11 patients aspirin was associated with tirofiban infusion. The rate of intra-stent aggregation during the procedure was 34.6% (N=26). Only one of the patients with intra-stent aggregation did not receive rescue therapy of intra-procedural aggregation. This patient did not present stent occlusion at 24 h. Of the remaining 25 patients who underwent some salvage therapy; In 11 cases, intra-stent aspiration was used, associating 2 occlusions of the stent at 24h. In another 2 cases abciximab was used, 1 case with intra-stent angioplasty, in 7 cases tirofiban was used as rescue techniques and in the 4 rescue cases different techniques were combined, none of these cases presenting occlusion of the endoprosthesis at 24 h. The 24-hour follow-up of the endoprosthesis was performed by Doppler ultrasonography, and its use was not possible in 7 cases due to death or instability of the patient that prevented its performance. Among the remaining 68 cases, the stent reocclusion rate was 8.82% (N=6). Of the 6 patients who presented reocclusion, only 2 of them had presented intra-stent aggregation during the procedure, performing rescue techniques (aspiration). In the remaining 4 no platelet aggregation phenomenon appeared during the procedure. Among the cases that presented aggregation during the procedure, only two were occluded at 24 h (7.69%). Regarding antiplatelet therapy, 81.8% (N=9) of those who were administered aspirin monotherapy compared to 0% of those who were administered tirofiban had carotid reocclusion.

It is known that the process of thrombus formation involves a mutual interaction between the coagulation system and the platelet activation system. If there is damage to the vascular endothelium (e.g. after angioplasty and/or stenting), glycoprotein (GP) receptors Ia/IIa and Ib/V/IX in platelets, react with von Willebrand factor and collagen in subendothelial tissue and initiate a platelet adhesion reaction. Platelet adhesion stimulates platelet activation, which leads to the release of adenosine diphosphate (ADP) and other intragranular substances, the generation of thromboxane A2 from arachidonic acid, etc., and culminates in the activation of the GPIIb / IIIa receptor, which binds to fibrinogen. Simultaneously, in the coagulation system, there is activation of various tissue factor clotting factors, etc. that are released at the site of endothelial damage and fibrinogen is generated. Finally, the generated fibrinogen binds to activated GPIIb/IIIa on the surface of platelets, these platelets adhere to each other and a fibrin clot forms (23,24,29).

GPIIb/IIIa platelet receptor antagonists such as tirofiban prevent fibrinogen binding, as well as activation of von Willebrand factor (vWF), fibronectin, and vitronectin in platelets. As a result, they suppress both platelet thrombus formation and fibrin deposition on established thrombi (23,24).

In cardiovascular studies from which most of the evidence on arterial reocclusion comes, GPIIb/IIIa inhibitors have been administered in patients undergoing procedures, such as percutaneous coronary intervention (PCI), such as an intravenous bolus (IV) followed by a continuous maintenance infusion. The purpose of this protocol is to use the initial bolus to reduce acute platelet activation, aggregation, and thrombus formation. Maintenance infusion eliminates the possibility of new thrombi forming until the patient is switched to an oral antiplatelet regimen. Intraoperatively, this approach prevents thrombogenic property of stents and coils, without significantly increasing the rate of hemorrhagic complications (23,24,30).

In summary, it seems that the use of antiplatelet therapy during the endovascular procedure does not increase the hemorrhagic risk, helping to reduce the risk of reocclusion, which clearly worsens the patient's prognosis (16–18). The limitation remains the heterogeneity of the action protocols, which prevents the generalization of effective, safe and standardized management of antithrombotic therapy in these patients. Given the pharmacokinetic characteristics, tirofiban has an advantage over aspirin due to its rapid action and the absence of pharmacological resistance. However, solving this therapeutic doubt is the starting point of our project.

# 5.- HYPOTHESIS AND OBJECTIVES OF THE TRIAL

- 1. **Hypothesis**

There is currently no standard criterion for the antiplatelet therapy of choice in acute stroke patients with tandem lesion. Current guidelines contraindicate the use of intravenous aspirin in the first 90 minutes after administration of intravenous fibrinolysis, which limits the antiplatelet therapy essential to address TL. The use of antithrombotics such as glycoprotein IIb/IIIa inhibitors is recommended in clinical trials.

The hypothesis of this study postulates that in patients with acute stroke and TL in the first 24 hours who require the placement of an extracranial stent, treatment with tirofiban is safe and decreases the rate of acute occlusions of the stent in the first 24 hours compared to standard antiplatelet therapy with ASA.

# Objectives

**Main objective:** To assess the efficacy and safety of the use of tirofiban versus aspirin in patients with ischemic stroke secondary to tandem lesion, by determining the rate of reocclusion of the stent in the acute phase of treatment at 24h as well as the determination of the rate of symptomatic bleeding defined as any hemorrhagic transformation associated with a worsening of 4 or more points on the NIHSS scale within the First 36 hours post-randomization.

# Secondary objectives:

- - - Determine the rate of salvage therapy in the presence of intra-stent aggregation phenomena during the procedure
    - Detection of good functional prognosis rates at 90 days in the different subgroups (defined as a modified Rankin scale (mRS) score between 0-2).
    - Reocclusion or significant restenosis at 30 days.
    - To improve pathophysiological knowledge of the complications associated with endovascular treatment of tandem lesions (re-occlusion) through the identification of related plasma biomarkers (biomarker sub-study).
    - Assessment of any of the biomarkers identified as a therapeutic target of reocclusion (biomarkers sub-study).

# 6.- TEST DESIGN

# Trial variables

# Primary endpoint

Main outcome variable: Rate of stent reocclusion in the acute phase of treatment at 24h.

Security primary variable:

Determination of symptomatic bleeding rate, defined as any hemorrhagic transformation associated with worsening of 4 or more points on the NIHSS scale within the first 36 hours post-randomization.

# Secondary variables

- - - - Rate of rescue therapy in the presence of intra-stent aggregation phenomena during the procedure
      - Rate of good functional prognosis at 90 days (defined as a modified rankin scale (mRS) score between 0-2).
      - Reocclusion or significant restenosis at 30 days.
      - To improve pathophysiological knowledge of the complications associated with endovascular treatment of tandem lesions (re-occlusion) through the identification of related plasma biomarkers (biomarker sub-study).
      - Assessment of any of the biomarkers identified as a therapeutic target of reocclusion (biomarkers sub-study).

# Design

Phase IV, randomized, controlled, multicenter clinical trial to assess the efficacy and safety of the use of tirofiban against acetylsalicylic acid in patients with ischemic stroke secondary to tandem lesion, undergoing recanalization therapy by endovascular treatment. The evaluator of clinical and radiological variables during follow-up will be blind to the randomization group of each patient.

# Randomization procedure

Once the patient's eligibility has been confirmed and informed consent for participation has been received (section 12.1)), randomization will proceed, which

It will be done *online*, centralized, for a ratio 1:1 (Tirofiban vs Control). The administration of the trial medication will be carried out as early as possible after the femoral puncture and always prior to the placement of the cervical stent. A maximum delay of 10 minutes after stent placement shall be allowed for the initiation of trial antiplatelet medication.

The system for randomization will be available online in the data collection notebook designed for this purpose. The randomization list will be kept by the Clinical Research and Clinical Trials Support Unit of the Virgen del Rocío University Hospital (UICEC-HUVR). Once he has entered that he meets all the inclusion criteria and none of the exclusion criteria, demographic data and previous fibrinolytic treatment, the researcher will obtain the assigned treatment and the patient code, which will be composed of a numerical code that identifies center and patient (eg XX-XX).

# Masking

Since the clinical trial is open, there will be no masking in this regard, and the patient may know the group to which he belongs. Both the control and experimental groups will receive endovascular treatment, and the type of antiplatelet they receive will be assigned by randomization.

The investigator evaluating the primary and secondary objectives will be blinded to the group assigned to the patient (experimental vs. control). This evaluation will be carried out by anonymizing the data and de-assigning to the treatment group. The investigator performing this evaluation cannot assist in the recruitment or follow-up of study patients and will be defined prior to initiating the study.

# Trial treatments

Experimental treatment and control**:** described in section 3.4

Auxiliary medication: as part of routine clinical practice, both groups should receive double antiplatelet therapy with oral ASA and clopidogrel before 24 hours of stroke evolution. The use of other auxiliary medication necessary for conscious or deep sedation is at the discretion of the center and will be collected as concomitant medication.



# Patient follow-up

Clinical follow-up will be carried out on all patients included in the visits defined in this protocol with anamnesis, examination, clinical scales, detection of adverse events, CT analysis and angio-CT of the skull at the time of

inclusion. Clinical follow-up and detection of adverse events will also be carried out at 24-36 hours, at discharge and 3 months after follow-up. The vascular study will be repeated by Doppler ultrasonography and/or angio-CT 24-36 hours after the endovascular procedure and one month after follow-up.

The schedule of visits is specified in section 8.4.

# Study completion or discontinuation criteria

Premature interruption of the clinical trial may occur due to a decision of the regulatory authorities, a change in the opinion of the Clinical Research Etich Committees, safety and/or drug problems or indications of ineffectiveness.

The investigator, as well as the sponsor, reserve the right to discontinue the study at any time for reasonable medical and/or administrative reasons.

# Study medication

The use of experimental drugs (low dose regimen of tirofiban) and control (ASA iv) will be managed and requested through the usual channels of the National Health System, specific labeling for clinical trial will not be carried out.

The centers will receive the study medication in their corresponding Pharmacy Services by their usual means of pharmaceutical management and its dispensing will be made to the teams according to the internal rules established in each center that will be collected in the study documents and in its corresponding pharmacy file. Only the maintenance of a registration system will be specifically requested to maintain the traceability of study drugs, both experimental and control.

The rest of the drugs used in the treatment of patients are approved for the treatment of the indication in which they are used in this study.

# Storage and dispensing of medication

The medication will be stored in the Pharmacy Service of each center under the storage conditions specified by the manufacturer for such products.

The dispensing will be carried out according to the standard procedures of each participating center, always keeping an administration control. A record of the dispensing shall be made by means of the sheets intended for this purpose, noting the batch, the expiry date and the number of units supplied.

# End of the trial

The day of the final visit of the last patient included in the study will be considered the end of the trial.

# 7.- SELECTION CRITERIA

The study population will consist of adult patients (18 years or older) with acute ischemic stroke of anterior territory secondary to tandem lesion, with indication for thrombectomy treatment according to the current recommendations of the Clinical Practice Guidelines and need for cervical stent placement.

# Inclusion criteria

- - 1. Patients with acute ischemic stroke of the anterior territory secondary to tandem lesion, with indication for thrombectomy treatment, and need for cervical stent placement.
    2. The locations of intracranial occlusion that may be included are: carotid "T", segments M1 and M2 of the middle cerebral artery, segment A1 of the anterior cerebral artery; and in the event that the posterior cerebral artery has fetal origin and is the site of intracranial occlusion.
    3. ASPECTS (Alberta Stroke Program Early CT Score) ≥6.
    4. Greater than or equal to 18 years of age.
    5. Informed consent.

# Exclusion criteria

- - 1. Under 18 years of age.
    2. Patients with ASPECTS < 6.
    3. Bilateral stroke or that occur due to simultaneous occlusion of any artery of anterior and posterior territory.
    4. Patients whose carotid stenosis is due to re-stenosis or reocclusion of a pre-existing cervical stent.
    5. Patients who are candidates for carotid puncture.
    6. Previous treatment with double antiplatelet therapy for another cause prior to inclusion.
    7. Severe comorbidity and/or reduced life expectancy.
    8. Modified Rankin scale (mRS) >2.
    9. Severe allergy to contrast dye.
    10. Pregnant.
    11. Patients with intracranial occlusive arterioclerotic disease or extra or intracranial dissection.
    12. Documented allergy to ASA or tirofiban.
    13. Personal history of thrombocytopenia (Platelets <100,000), or of having developed thrombocytopenia during previous administration of GP IIb/IIIa receptor antagonists or ASA iv.
    14. Concomitant anticoagulant therapy (with presence of INR >1.7 in case of treatment with anti-vitamin K or taking direct oral anticoagulant makes

<48 hours).

- - 1. Active or recent bleeding (within 30 days prior to antiplatelet therapy) clinically relevant (e.g., gastrointestinal bleeding).
    2. Active peptic ulcer in the last 3 months.
    3. Medical history of the patient that involves high bleeding risk at the discretion of the investigator.

# Withdrawal criteria

According to the Declaration of Helsinki (Annex V), patients have the right to withdraw from the study at any time and for any reason (Annex XIV), being able to express this personally or through their representative.

The administration of the trial medication will be carried out as early as possible after the femoral puncture and always prior to the placement of the cervical stent. A maximum delay of 10 minutes after stent placement shall be allowed for the initiation of trial antiplatelet medication. In case of exceeding this time the patient will be removed from the trial.

# By security criteria

Any adverse event that, at the discretion of the clinician, requires the withdrawal of the study medication.

When, for any reason, the treatment is no longer safe for the patient. Or for any other reason that may endanger the life of the patient or have serious consequences for the same.

Subjects who have discontinued the study as a result of adverse events will receive appropriate alternative treatment and the investigator should record the reason for discontinuation of the study, facilitate or schedule appropriate follow-up (if necessary) of these subjects and document the evolution of the subject's condition. All medications administered up to the time of discontinuation must also be recorded in the concomitant medication section of the data collection booklet.

# For non-compliance or violation of the rules contained in the protocol

When the patient no longer meets the standards of the trial, it may be withdrawn at the discretion of the responsible investigator or due to loss of follow-up.

# Follow-up of patients withdrawn prematurely

If a patient is prematurely removed from the trial, the investigator will provide the primary reason for the discontinuation and, as indicated by GCP standards, procedures will be followed according to standard treatment protocols for their pathology at the discretion of the responsible clinician.

# 8.- TREATMENT OF SUBJECTS

**Experimental Group**

# Tirofiban low-dose regimen;

- Intravenous bolus of 500 micrograms of tirofiban to pass in 5 minutes with pump. The pump will be programmed at 120 ml / h to pass in 5 minutes, which is equivalent to 10 ml (500 micrograms) of tirofiban.
- Dose reduction after 5 minutes to 200 micrograms / hour. The infusion pump should be programmed at 4 ml/hour for 24 hours (maximum total infused dose of 96 ml).

The experimental treatment will be administered in the interventional neuroradiology room, once the patient has accepted its inclusion in the study and has been assigned to the experimental group according to randomization. This drug should be administered as early as possible after the femoral puncture and always prior to the placement of the cervical stent. A maximum delay of 10 minutes after stent placement will be allowed for the initiation of antiplatelet medication. During the first 24 h post-procedure, the infusion of tirofiban initiated in the neuroradiology room should be maintained at a rate of 4ml / hour, until the CT of the control skull. The management of post-procedure blood pressure will be established based on clinical practice guidelines

habitual. Co-administration of unfractionated heparin with tirofiban will not be necessary, given the increased risk of intracranial hemorrhagic transformation.

Since the dosing schedule is low-dose, both the bolus and the infusion are below the recommended dose threshold for patients with severe renal impairment. In any case, an analytical control should be carried out within the first 24 hours after the administration of Tirofiban (earlier if necessary due to the presence of bleeding complications). Renal function, blood count (for hemoglobin and platelet control), and coagulation will be determined. If new-onset thrombocytopenia is confirmed, treatment with tirofiban should be discontinued.

A control CT scan will be performed 20 hours (+/-2h) after endovascular treatment. Once the absence of parenchymal hematoma (HP1 or HP2, according to ECASS criteria) is demonstrated, loading doses of ASA (300 mg) and clopidogrel (300 mg) will be administered in the tirofiban group, and the infusion of tirofiban and the double oral antiplatelet therapy must be maintained simultaneously for 4 hours, after which the administration of the experimental drug should be suspended. In this way, the tirofiban infusion pump will be maintained for up to 24 hours (+/- 2 hours) after the procedure. Oral antiplatelet medication may be administered orally if the patient has a good level of consciousness and does not associate dysphagia; or by placing a nasogastric tube.

**Control group**

**Intravenous acetylsalicylic acid**

There is no standardized antithrombotic management of ischemic strokes secondary to tandem lesion, however, in order to perform carotid angioplasty and the subsequent placement of cervical stents for the stabilization of atheromatous plaque, it makes the use of at least one antiplatelet almost mandatory, being intravenous acetylsalicylic acid the most accessible and widespread, being considered as the reference therapy.

Intravenous acetylsalicylic acid (Inyesprin ®), considered as a medicine, and which will be administered to the control group, contains lysine acetylsalicylate as its active ingredient.

Each vial contains 900 mg lysine acetylsalicylate (equivalent to 500 mg acetylsalicylic acid). The excipient is: glycocola.

# Protocol Administration of intravenous acetylsalicylic acid:

Each bottle of SOLUTION FOR INJECTION contains 1 gram of D,L-lysine-glycine acetylsalicylate (equivalent to 500 mg acetylsalicylic acid). This solution for injection is accompanied by 5 ml of water for injection. A single dose of 500 mg iv will be administered.

**Method of administration**:

Transfer the diluent to the vial with the powder and shake vigorously until the powder is completely dissolved. The solution for injection should be prepared immediately and used immediately after preparation. As previously described, it is advisable to administer ASA as soon as possible after femoral puncture and always before stent placement, allowing a delay of 10 minutes after placement of the cervical stent. In case of exceeding this time, the patient will be removed from the trial. Only a clear, newly prepared solution should be administered. The method of administration may be:

- Slow intravenous route
- By infusion (1 vial of IV ASPIRIN® in not more than 250 ml in 0.9% sodium chloride solution, 5% and 10% glucose solution, Ringer's or lactated Ringer's solution),
- Slowly through a 3-way key no more than 250 ml in sodium chloride solution 0.9%, glucose solution 5% and 10%, Ringer solution or lactated Ringer)

The control treatment will be administered under the same conditions as those explained for the experimental therapy, in the interventional neuroradiology room, once the patient has accepted his inclusion in the study and has been assigned to the control group according to randomization.

This drug should be administered as early as possible and always before cervical stent placement. It will not be necessary to maintain aspirin infusion. In the AAS group, a CT scan of the skull will also be performed 20 (+/-2 hours) after the endovascular procedure. Once the absence of parenchymal hematoma (HP1 or HP2, according to ECASS criteria) is demonstrated, clopidogrel (300 mg) and 100 mg ASA will be administered. Oral antiplatelet medication may be administered orally if the patient has a good level of consciousness and does not associate dysphagia; or by placing a nasogastric tube.

In both groups (experimental and control), double antiplatelet therapy will be maintained for 1 month after endovascular treatment, unless there are clinical reasons that do not advise it. After one month, clopidogrel should be discontinued while maintaining long-term ASA.

**Other medication**

Patients included in this trial may require the administration of another concomitant medication during endovascular therapy. According to usual clinical practice, the medication that can be used during thrombectomy is reflected in ANNEX VI.

# Duration and adjustments of treatment

The loading dose of oral antiplatelet agents will be administered as long as the existence of hemorrhagic transformation is ruled out in the control skull CT at 20 +/- 2 h.



# Contraindicated medication

For tirofiban

- - - Incompatibility with diazepam so they should not be administered in the same intravenous line

For AAA Injection

- - - Methotrexate at doses greater than 15 mg per week, with anti-inflammatory doses of ASA or analgesic or antipyretic doses: Increased toxicity of methotrexate especially hematologic toxicity (due to a decrease in renal clearance of methotrexate caused by ASA).
    - Oral anticoagulants with acetylsalicylic acid used at anti-inflammatory doses, or at analgesic or antipyretic doses and in patients with a history of gastroduodenal ulcer: Increased risk of bleeding.

# Concomitant medication

All patients should receive treatment for their stroke in accordance with usual medical practice (ANNEX VI).

# Rescue medication

Based on good clinical practice, all patients who leave the study prematurely will be recommended an alternative treatment. If withdrawal is due to a serious adverse event, patients will be monitored by the investigator or his/her designee until appropriate completion, i.e. until the adverse event disappears or is determined to be permanent.

# Calendar of visits and evaluations

| **Procedures** | **Visit 1 Inclusion** | **Visit 2 (Day 0) Admon medication** | **Visit 3**  **24-36 hours** | **Visit 4 Hospital discharge** | **Visit 5**  **Follow-up 30 days post-inclusion**  **(± 7d)** | **Visit 6 (90 days post-inclusion (± 7d) –**  **Telephone** |
| --- | --- | --- | --- | --- | --- | --- |
| Consent  informed | X |  |  |  |  |  |
| Review criteria of incl/excl.. | X |  |  |  |  |  |
| Randomization | X |  |  |  |  |  |
| History / DemograpsICH | X |  |  |  |  |  |
| Vital signs (TA, HR, FR,  weight) | X | X | X | X | x |  |
| Hemogram | X |  | X | X | X |  |
| Biochemistry | X |  | X |  |  |  |
| Coagulation | X |  | X | X | X |  |
| NIHSS Scale | X |  | X | X |  |  |
| Pregnancy test | X |  |  |  |  |  |
| Electrocardiogram  ma | X |  |  |  |  |  |
| Modified Rankin scale (pre-stroke in  Visit 1) | X |  |  | X |  | X |
| CT and Angio- | X |  |  |  |  |  |
| CT scan of skull |  |  | X |  |  |  |
| Signs and  Stroke symptoms | X |  |  |  |  |  |
| Biomarker sub-study |  | X |  |  |  |  |
| Admon Tirofiban/drug  control |  | X |  |  |  |  |
| Doppler TSA |  |  | X |  | X |  |
| Medication  concomitant | X | X | X | X | X | X |
| Adverse events |  | X | X | X | X | X |

*CT angiography will be performed 24 hours after the procedure in case of doubts in the Doppler, or existence of severe restenosis or intra-stent occlusion. TA, blood pressure; HR, heart rate; RF, respiratory rate; NIHSS: *National Institute of Health Stroke Scale*; TSA: supraaortic trunks.

# Procedures per visit

The follow-up of the patients in the present study consists of 5 scheduled visits (all face-to-face) and a last telephone visit 90 days post-inclusion. One month after the administration of the study medication, it will be necessary to perform an ultrasonographic / angio-CT test to control the cervical stent, in addition to determining the score on the modified rankin scale. In this protocol the visits are ordered chronologically and in parentheses the day or interval of days of the study in which they must be carried out is recorded.

For the purposes of this study, "Day 0" will be considered to be the day of initiation of study medication. The following sections detail the procedures to be carried out at each visit.

# Inclusion Visit (Visit 1)

The patient potentially eligible for the study will be informed of the objectives of the study and the overall requirements, before any specific evaluation of the study the patient must accept their participation (section 12.1).

Below are the procedures that will be carried out in this visit:

- - - - Informed consent of the study (section 12.1; record in medical records and provide the patient with a copy of the information sheet and IC) (Annex VII)
      - Clinical and analytical assessment of inclusion and exclusion criteria.
      - Informed consent (section 12.1) and randomisation
      - Clinical history and anamnesis. Patient demograpsICH
      - General physical exam including weight and vital signs
      - Verification of inclusion and exclusion criteria
      - Review of concomitant medication
      - Signs and symptoms of stroke will be recorded, as well as the time of onset of symptoms or the last normal view of the patient.
      - NIHSS scale (Annex VIII). Modified pre-stroke Rankin scale.
      - Blood count, coagulation, biochemistry.
      - Performing electrocardiogram
      - Stratified randomization to control group (ASA) or experimental group (Tirofiban) according to the use of intravenous fibrinolysis.

Once the results of all the procedures mentioned above have been obtained, and it has been verified that all the inclusion criteria and none of exclusion are met, the patient may continue in the main study, with voluntary participation in the sub-study of biomarkers, whose consent must also be signed (Annex VII). If, on the other hand, the patient does not meet the eligibility criteria, he will not be able to continue in the study and must register in the CRD the reason for the non-selection of the patient.

The patient's acceptance to participate in the Attila/ictus/2021 clinical trial (section 12.1), date of signature of consent, assigned patient number and study sponsor must be recorded in the patient's medical record. The patient will be given a copy of the patient information sheet and informed consent, as well as an identification card stating that the patient is participating in the clinical trial.

# Visit 2 (Day 0, Start of treatment)

This visit will be performed on all patients included in the trial.

All randomized patients will have a bioconnector placed (if not already placed) in the non-paretic arm if possible, to initiate the administration of the drugs (detailed description in ANNEX IX).

Below are the procedures that will be carried out in this visit:

- - - - Collection of the date of the visit.
      - Vital signs (blood pressure, heart rate, breathing rate, oxygen saturation).
      - Administration of experimental medication (tirofiban) or control (ASA): Time of initiation of antiplatelet therapy: aspirin or tirofiban (in this case also indicate the time of initiation of the infusion).
      - Stent used in extracranial stenosis.
      - Presence of phenomena of intra-stent aggregation or occlusion thereof; indicate in this case the use of rescue therapy if appropriate.
      - Score on the mTiCI recanalization scale (Annex X) at the intracranial level and presence of residual stenosis at the cervical level.
      - If patient has agreed to participate in the biomarker study (voluntary participation) → Extraction of blood samples for the sub-study of biomarkers by venipuncture of peripheral blood (during or immediately after the completion of mechanical thrombectomy).
      - Control of the dispensing and administration of medication.
      - Review of concomitant medication.
      - Assessment of adverse events.

# Visit 3 (24-36 hours after administration of antiplatelet drug)

This visit will be made to all patients included in the clinical trial between 24-36 hours after signing the informed consent in both groups.

All patients will be transferred to the stroke unit or in selected cases (especially if they have been intubated) to the ICU, making the reception by the usual procedure. During these first 24 h post-procedure, the infusion of tirofiban initiated in the neuroradiology room should be maintained at a rate of 4ml / hour, for 24 hours after the beginning of said infusion. The management of post-procedure blood pressure will be established based on routine clinical practice guidelines.

Below are the procedures that will be carried out in this visit:

- - - - A control CT scan will be performed at 20 hours (+/-2h) hours of endovascular treatment regardless of the randomization group (experimental or control). Once the absence of parenchymal hematoma (HP1 or HP2, according to ECASS criteria) is demonstrated, the process to be followed will be different depending on the experimental or control group:
        - In the experimental group (tirofiban) will proceed to administer loading doses of ASA (300 mg) and clopidogrel (300 mg), having to maintain simultaneously the infusion of tirofiban and the double oral antiplatelet for 4 hours after the realization of the control CT, after which the administration of the experimental drug should be suspended. In this way, the tirofiban infusion pump will be maintained for 24 hours (+/- 2 hours) after the procedure.
        - In the control group (ASA), a CT scan of the skull will also be performed 20 (+/-2) hours after the endovascular procedure. Once the absence of parenchymal hematoma (HP1 or HP2, according to ECASS criteria) is demonstrated, clopidogrel (300 mg) and 100 mg ASA will be administered.
      - After these examinations, the following should be collected in the CRD:
- Score on the ASPECTS scale (Annex XI)
- Presence of hemorrhagic transformation according to ECASS criteria (Annex XII).
  - - - Ultrasonographic control by Doppler of the stent at 24 hours after the procedure by an evaluator blind to the randomization group assessing:
- Presence of residual intra-stent stenosis ≥70%: defined as a peak systolic velocity (SVR) ≥ 300 cm/s.
- Intra-stent reocclusion: which will be defined by the presence at the level of the occlusion point, by a characteristic biphasic, brief and low-speed pattern both in the Doppler spectrum and in color mode (color image with both orthodromic and antidromic flow, red-blue just proximal to the occlusion). In addition, the image detected in B mode will show an anechoic appearance with a false appearance of permeable light, detecting absence of flow in color and Doppler mode.
- In case of severe stenosis or Doppler occlusion, CT angiography will be performed to confirm Doppler findings.
  - - - Clinical data:
- Date of visit.
- Physical examination and NIHSS score
- Presence of symptomatic intracranial hemorrhage defined as cerebral hemorrhagic transformation associated with worsening of 4 or more points on the NIHSS scale.
- Analytical study with blood count, biochemistry and coagulation (safety analysis).
- Control of dispensing/administration of medication.
- Review of concomitant medication
- Assessment of adverse events.

# Visit 4 (Follow-up at hospital discharge)

This visit will take place on the day of hospital discharge.

Below are the procedures that will be carried out in this visit:

- Date of visit
- Physical examination and NIHSS score.
- mRS score
- Analytical study with blood count and coagulation (safety analysis)
- Review of concomitant medication
- Assessment of adverse events.

# Visit 5 (Follow-up 30 days post-treatment, ± 7 days)

Below are the procedures that will be carried out in this visit:

- Ultrasonographic control by Doppler assessing the presence of intra-stent restenosis ≥70% or its reocclusion, as described in visit 3:
  - In case of severe stenosis or Doppler occlusion, CT angiography will be performed to confirm Doppler findings.
- Analytical study with blood count and coagulation (safety analysis)
- Review of concomitant medication.
- Assessment of adverse events.

# Visit 6 (follow-up 90 days post-inclusion ± 7 days). Telephone

- Clinical variables being reflected in the clinical history and CRD:
  - Modified rankin scale mRS.
- Review of concomitant medication.
- Assessment of adverse events.

# Unscheduled visit

- Physical examination and NIHSS score.
- Analytical study with blood count and coagulation (safety analysis)
- Review of concomitant medication.
- Assessment of adverse events.

# 9.- ASSESSMENT OF EFFECTIVENESS

# Primary and secondary efficacy endpoints

Main outcome variable: Rate of stent reocclusion in the acute phase of treatment at 24h.

Secondary efficacy endpoints:

- - - Rate of rescue therapy in the presence of intra-

stent during the procedure.

- - - Rate of good functional prognosis at 90 days (defined as a modified rankin scale (mRS) score between 0-2).
    - Reocclusion or significant restenosis at 30 days.
    - To improve pathophysiological knowledge of the complications associated with endovascular treatment of tandem lesions (re-occlusion) through the identification of related plasma biomarkers (biomarker sub-study).
    - Assessment of any of the biomarkers identified as a therapeutic target of reocclusion (biomarkers sub-study).

# Other studies

The research project includes a sub-study of biomarkers that has a specific informed consent and whose participation is optional by the patient. For more information, see Annex XIII.

# 10.- SECURITY ASSESSMENT

# Security primary variable

Determination of symptomatic bleeding rate, defined as any hemorrhagic transformation associated with worsening of 4 or more points on the NIHSS scale within the first 36 hours post-randomization.

# Security Assessments

The following clinical evaluations will be performed to assess the safety profile of the trial treatment.

# Physical examination, vital signs

A physical examination and a constant sampling will be carried out in each of the face-to-face visits.

# Laboratory tests

At baseline (Visit 0), prior to the start of study medication, and within the first 24-36 hours of randomization (Visit 3), blood samples will be obtained for the following analytical determinations:

- - - - Blood count: red blood cells, hemoglobin, hematocrit, leukocytes, neutrophils, lymphocytes, monocytes, eosinophils, basophils, platelets.
      - Plasma coagulation study (PATT).
      - General biochemistry: glucose, urea, creatinine, sodium, calcium, potassium.

At visits 4, 5 (at discharge and 30 days after inclusion), blood samples will be obtained for the following analytical determinations (safety study):

- - - - Blood count: red blood cells, hemoglobin, hematocrit, leukocytes, neutrophils, lymphocytes, monocytes, eosinophils, basophils, platelets.
      - Plasma coagulation study (PATT).

# Adverse events of interest for follow-up

The low-dose regimen of tirofiban has been previously used in several clinical studies of patients with ischemic stroke secondary to tandem lesion, showing sufficient safety data to consider its use in a clinical trial. However, the major complication to be taken into account in the experimental group is the increase in the rate of intracranial hemorrhage, whether symptomatic or not. Therefore, a CT scan of the skull will be performed on all patients 24 hours after the start of the procedure, before there is clinical neurological worsening of the patient.

Adverse effects arising from the catheterization approach are possible: inguinal hematoma requiring transfusion. These effects derived from the approach route represent a rare complication whose incidence is less than 1%. The evolution of arterial access will be monitored for the diagnosis and early treatment of a femoral pseudoaneurysm, as well as perforation or laceration of any arterial branch related to arterial access.

More information in the data sheet (Annex II) and (Annex XIV)

# Pharmacovigilance definitions Adverse Event (AA):

An adverse event is any unwanted medical reaction experienced

by the patient at any time during the course of the study, whether or not considered related to the study treatment. This definition includes the emergence of a new disease and the exacerbation of pre-existing conditions other than the indication under study.

# Adverse Reaction (AR):

An RA is any harmful, unintended reaction to an investigational drug, regardless of the dose administered.

# Serious Adverse Event (SAA) and Serious Adverse Event (RAG):

AEs or RAs which, at any dose, may cause death, threaten the life of the subject, require hospitalization of the patient or prolong an existing hospitalization, cause permanent or significant disability or disability, or result in a congenital anomaly or malformation are considered serious. Suspicions of medically significant AA or RA are also considered serious, even if they do not meet the above criteria, including major medical events that require intervention to prevent one of the consequences described above. All suspected transmission of an infectious agent via a medicinal product shall also be reported as serious.

The concept "life-threatening" in the definition refers to the fact that, in the opinion of the investigator, the patient at the time of AA or RA is at real risk of death; it does not mean that AA/RA hypothetically could have caused death if it had been more intense.

The concept "require hospitalization" will exclude both planned hospitalizations for scheduled treatments and those that have been planned or anticipated prior to the start of the study in relation to a pre-existing medical condition.

# Unexpected Adverse Reaction (UAR):

Any RA whose nature, intensity or consequences do not correspond to the reference safety information.

# Unexpected Serious Adverse Reaction (USAR):

RAG (defined previously), the nature, severity or consequences of which do not correspond to the reference safety information.

# Causality Criteria:

- Related AA: The temporal relationship of AA to the medication under study indicates a possible causal relationship and cannot be explained by factors such as the patient's clinical status, therapeutic interventions.
- Unrelated AA: The temporal relationship of AA to study medication indicates an unlikely causal relationship, or other factors (medication or concomitant conditions), other therapeutic interventions provide a satisfactory explanation for AA.

# Reference Safety Information

Data sheets: ANNEXES II and III

# Reporting and collection of serious adverse events

The principal investigator or a collaborator shall report to the Pharmacovigilance department PD-UICEC-HUVR, all serious adverse events (as defined below), whether or not considered treatment-related, expected or not, within 24 hours (one working day), of his knowledge (Annex XV). Serious adverse events occurring at any time after the patient's inclusion in the study (defined as the time when the subject's participation in the study is consented to, paragraph 12.1) and up to 30 days after the subject concludes or leaves the study. A subject is considered complete EITHER after the conclusion of the last visit or contact (e.g., telephone contact with the investigator or a collaborator), as indicated in the protocol evaluation schedule, OR after the last dose of study medication, whichever is later. Withdrawal is defined as the date on which a subject and/or the investigator determines that the subject can no longer meet the study requirements at any subsequent visits and evaluations.

The investigator shall complete and sign the AAG notification form (Annex XV) which he or she shall send by fax or e-mail to:

Clinical Research and Clinical Trials Unit Virgen del Rocío University Hospital

Pharmacovigilance Department: [pv_atila@scren.es](mailto:pv_atila@scren.es) Avda. Manuel Siurot S/N 41013. Seville

Phone: 955 01 34 14

Fax: 955095338

PD staff will review the form received and, if appropriate, request additional information from the investigator. The investigator shall provide information to the sponsor or to whoever assumes the tasks delegated by the sponsor (PD-UICEC-HUVR Unit) whenever requested and in any case when his initial assessment changes as to severity or causality. The reporting procedure described above shall be followed for the communication of monitoring information.

PD-UICEC-HUVR staff shall keep detailed records of all AAGs or of special interest communicated to them by researchers.

In the event of a medication error or the investigational medicinal product being used outside the protocol during the course of the study, the investigator shall notify the PD-UICEC-HUVR within 24 hours of becoming aware of it. The circuit for notification and the form will be the same as for AAGs.

AA that meets the severity criteria described in definition section 10.1 shall be considered serious. Clinically significant events that do not result in death, are life-threatening or require hospitalization may be considered serious adverse drug experiences when, based on appropriate medical judgment, they may endanger the subject or require medical or surgical intervention to prevent one of the outcomes listed in this definition. Examples of such medical events are allergic bronchospasm requiring intensive treatment, at home or in an emergency unit, blood dyscrasia or seizures that do not result in hospitalization, or development of dependence or substance abuse.

Laboratory test alterations are also required to be reported, unless otherwise stated in this section of the protocol.

# Exceptions to the collection of standard AA

When there is a deterioration of the disease under study, uncertainty may arise as to whether it is lack of efficacy of the test medication, progression of the disease or constitutes an AA. In these cases, unless the sponsor or the notifying physician considers that the study treatment contributed to the deterioration of the disease or local regulations state otherwise, such deterioration shall not be considered as AA but as loss of efficacy or progression of the disease if they meet the following definitions:

- - - - Loss of efficacy: Insufficient therapeutic effect collected as a result of efficacy. Discontinuation due to insufficient therapeutic effect (i.e. lack of efficacy) should not be listed as AA. A clinical failure should not be recorded as AA.
      - Disease progression: The progression of the disease can be considered as a worsening of the subject's condition attributable to the disease for which the different treatments of disease are being studied.

This worsening may consist of an increase in the severity of the disease under study and/or an increase in the symptoms of the disease. If it is an expected progression, unless it is more severe in intensity or more frequent than expected for the study condition treated in the trial it should not be recorded as AA.

Any event or hospitalization that is prolonged due to disease progression should not be recorded as a SAA, unless it is believed that the study drug has actively contributed to the progression of the disease, (insufficient therapeutic effect is not considered here).

In this sense, AA/AAG derived from or related to the reocclusion or restenosis of the stent will not be notified (these will be collected for evaluation as study variables in the data collection notebook) and with the progression of the study disease, events that due to their expected within the evolution of the pathology under study will not require specific notification to the Pharmacovigilance department (it will not be necessary to complete the PD form). SAES). The most common ones are described below:

- - - - Symptomatic or asymptomatic cerebral hemorrhage
      - Respiratory infection, urinary tract infection, phlebitis
      - Seizure
      - Pulmonary thromboembolism
      - Hyperperfusion syndrome.
      - Acute myocardial infarction

Events that are unequivocally associated with disease progression should not be reported as AA/AAG during the active study period, unless the outcome is fatal. It will be the cause that causes the death of the patient that is registered as AAG in the data collection notebook and notified by means of AAG form to PD-UICEC-HUVR within 24 hours of its knowledge.

# RAGI Expedited Notification

The PD-UICEC-HUVR department is responsible for notifying the AEMPS and the Autonomous Communities where the test is carried out, all the RAGI that are collected in the study, following the procedure indicated in current legislation.

The maximum period for notification of an individual case of suspicion of RAGI shall be 15 calendar days from the moment in which the sponsor has become aware of it. When the suspicion of RAGI has caused the death of the patient,

or endangering his life, the promoter will send the information within 7 calendar days from the moment he becomes aware of it. Complete this information, if possible, within 8 days.

This information should include an assessment of the significance and implication of the findings, including relevant previous experience with the same or similar medicinal products.

Likewise, the competent body of each of the Autonomous Communities where the test is carried out must be notified of suspected RAGI occurring in the health centers of its Community. In both cases, the RAGI notification form will be used for this purpose.

# Expeditious notification of other relevant safety information

The PD-UICEC-HUVR department shall notify as soon as possible and no later than 15 days after it becomes aware of any information that could modify the benefit/risk balance of the investigational medicinal product (e.g. increase in the percentage of occurrence of expected ARGs, AGRs occurring after the end of a clinical trial, new developments related to the conduct of the trial or development of the investigational medicinal product, any recommendations of the Data Monitoring Committee relevant to the safety of the subjects, etc.).

# Notification to investigators

The sponsor will present to the investigators safety information that could impact the safety of patients included in the study as soon as possible.

In addition, the investigator will be informed throughout the study about any safety aspects, including protocol modifications due to safety reasons.

# Medication errors

Medication errors are unintentional errors in prescribing, dispensing, administering or monitoring a medication while it is under the control of a healthcare professional or patient and which may cause harm to the patient.

Misuse refers to a situation in which the medical product is intentionally used inappropriately, not in accordance with the protocol.

Study medication errors and use outside the protocol will be recorded in the data collection notebook (CRD), regardless of whether they are associated with an AA/AAG or not. Misuse or abuse will be collected and reported in the safety database within 24 hours of the researcher's knowledge of it.

# 11.- STATISTICS

# Sample size calculation

For the calculation of the sample size, the ARCOSINE approximation was used. The following parameters were used: power 80%, alpha error 5%, estimated difference between two proportions of 22% (for control group, AAS) and 10% (for experimental group, tirofiban). With these considerations, and including a 5% loss, the sample size would be equal to 240 patients (120 in each group).

# Statistical analysis

DemograpsICH and baseline data

A descriptive analysis of all the demographic variables collected will be carried out, as well as the clinical data prior to the start of treatment. The qualitative variables will be expressed through absolute frequencies and percentages, while the quantitative variables will be presented through the mean, median, standard deviation, maximum, minimum and number of observations.

Effectiveness analysis

All p-values and confidence intervals will be calculated and evaluated using a bilateral confidence level of 95%.

The main efficacy endpoints will be analyzed by comparing the data corresponding to the baseline condition and follow-up by the t-test for paired samples if the variable meets the requirements of normality and by the Wilcoxon test for paired samples in another case. The unpaired t-student test will be used to compare the means from independent groups (intervention and control).

Security Analysis

The number and percentage of patients dropping out of the study due to adverse events, patients who have experienced at least one adverse event, the most common adverse events and patients who have experienced at least one serious adverse event shall be calculated. The 95% confidence interval will also be calculated. An interim analysis will be carried out, when the inclusion has reached 120 patients (60 from each group) to assess the safety of both therapies under study.

# Definitions of the study analysis populations

Intention-to-treat (ITT) population: All patients were randomized.

Population by protocol (PP): All patients who have received 90 full days of study treatment.

# 12.- ETHICAL ASPECTS

The trial will be carried out in accordance with the principles emanating from the Declaration of Helsinki, and according to current legal regulations (Royal Decree 1090/2015), and will not start until the approval of the reference CEIC has been obtained, the agreement of the directors of the Institutions, and the authorization of the Spanish Agency of Medicines and Health Products.

The investigator must meet all protocol requirements. If a situation occurs where a temporary deviation from the protocol is required, the investigator or other physician responsible for the patient should contact the monitor as soon as possible in order to discuss the situation and agree on an appropriate course of action. The investigator will document the deviation from the protocol and the circumstances that required it.

# Informed consent

The patient must give consent before being admitted to the clinical study (Annex VII). The physician shall explain the nature, purposes and possible consequences of the clinical trial in a manner understandable to the patient. The information provided by the doctor should also be recorded. In obtaining and documenting it, researchers will comply with the relevant legislation (Article 4 of Royal Decree 1090/2015), the rules of good clinical practice and the ethical principles that have their origin in the Declaration of Helsinki.

Informed consent will not be required in the event of an emergency. The patient may be included and administration of the study medication/procedures may be initiated without prior informed consent in the cases described in Article 35(1) and (2) of Regulation (EU) No 536/2014 of the European Parliament and of the Council of 16 April 2014:

- - - emergency situation caused by a sudden serious or life-threatening condition of the study population
    - The administration of study medication should be carried out as early as possible
    - It is a trial with a specific interest for the population in which the research is conducted.

This inclusion without prior IC should be recorded in the patients' medical records.

In the event of patients who are not able to make an informed decision, a family member/legal representative of the patient will be contacted by telephone to request the verbal telephone IC. This inclusion with verbal telephone IC should be recorded in the patients' medical records.

The investigator shall request the IC from the subject or his/her legally designated representative without undue delay, whichever is faster, and the subject or his/her legally designated representative, whichever is faster, shall be provided with the information about the study as soon as possible.

Where informed consent has been obtained from the legally designated representative, informed consent to continue participating in the clinical trial shall be obtained from the subject as soon as he or she is able to give it.

The subject of the study will give his consent, signing the corresponding form. The researcher will receive an appropriate number of informed consent models through the sponsor. To this end, each model must bear the signature of the investigator and the patient.

The investigator will not initiate any investigation for the trial until he or she has obtained consent by proceeding according to the assumptions described above.

# Data protection

The processing, communication and transfer of personal data of all participating subjects will comply with the provisions of Regulation (EU) 2016/679 of the European Parliament and of the Council of 27 April 2016 on the protection of natural persons with regard to the processing of personal data and on the free movement of such data and Organic Law 3/2018, of 5 December on the Protection of Personal Data and guarantee of digital rights. In accordance with the provisions of the aforementioned legislation, the patient may exercise the rights of access, modification, opposition and cancellation of data, for which he must contact his study doctor.

The anonymity of the subjects participating in the study will be maintained at all times. Thus, the data collected for the study will be identified by a code and only the researcher and collaborators will be able to relate these data with

the patient and his/her medical history. Therefore, the identity of the patient will not be revealed to any person except for exceptions: personnel authorized by the sponsor, when necessary, to verify the data and procedures of the study, but always maintaining the confidentiality of the same in accordance with current legislation; in case of medical emergency or legal requirement (health authorities: Spanish Agency for Medicines and Health Products and Local Committee for Clinical Trials).

The data from this study will be used only for the specific purposes of the study.

# Responsibilities of study participants

The participant must follow the instructions of the researchers and communicate any eventuality to them. The subject shall be duly informed of any prohibitions or restrictions to be observed during the conduct of the test.

Failure to comply with these recommendations will result in abandonment of the study.

All subjects participating in the study have the right to leave the study at any time, withdrawing their consent, without having to justify this decision and without this implying any detriment to their clinical follow-up. If this occurs, the investigator will try to have the subject perform all necessary assessments to ensure that no adverse events occur and to ensure appropriate follow-up in the event that any type of problem has occurred.

# Monitoring and auditing

The study will be monitored through local visits, telephone calls and periodic inspection of the CRDs frequently enough to verify the following:

- Rate of inclusion of patients, compliance with the rules of the protocol procedures, integrity and accuracy of the data entered in the notebooks, verification against the original documents and occurrence of adverse events.
- Monitoring visits will be conducted by study monitors. It is understood that these monitors will be able to access patients' medical records after being requested by the investigator. The researcher will dedicate the time sufficient for these visits and will facilitate access to all documentation for authorised persons.
- The study may be audited by an independent body. In the same way, members of the CEIC will be able to follow up on it.

# Premature termination or suspension of the study

If the trial is terminated prematurely or suspended, the sponsor should promptly inform the investigator and the regulatory authorities of the termination or suspension and the reason for it. The sponsor or investigator should promptly inform the EIRC and provide the EIB with the reason for the termination or suspension, as specified in the relevant regulatory requirements.

# Study documentation

The documentation related to the study (protocol, CRD, informed consent, authorizations) will be filed in a safe place and easily accessible by the research team. All information contained in clinical, histological, biochemical reports, observations or other activities is necessary for the reconstruction and evaluation of the study.

# 13.- FINANCING AND INSURANCE

# Financing

The project has received funding by public call for health research projects (AES 2021), modality health research projects of the Carlos III Health Institute with file number PI21/01322.

# Insurance

The promoter has contracted a civil liability insurance policy in accordance with the requirements specified in article 9 of RD 1090/2015.

# 14.- PUBLICATION POLICY

These will be adjusted to the provisions of Royal Decree 1090/2015 of December 4, which regulates Clinical Trials with medicines, the Ethics Committees of Research with medicines and the Spanish Register of Clinical Studies, article 42, which includes the following text:

*"1. The sponsor shall be obliged to publish the results, both positive and negative, of authorised clinical trials, preferably in scientific journals before they are disclosed to the non-health public, irrespective of the obligations to publish the*

*the results in the Spanish Registry of Clinical Studies (REec) and the provisions of Regulation (EU) No 536/2014 of the European Parliament and of the Council of 16 April 2014.*

1. *When studies and research works on medicines are made public, aimed at the scientific community, the funds obtained by the author, by or for their realization, and the source of financing will be stated.*
2. *The anonymity of the subjects participating in the trial shall be maintained at all times.*
3. *Treatments of as yet undetermined efficacy will not be announced prematurely or sensationally, nor will this be exaggerated. Intermediate results which may compromise the reliability of the final test results shall not be publicised.*
4. *The advertising of medicinal products for human use under investigation is strictly prohibited, as established in the revised text of the Law on guarantees and rational use of medicines and health products, in Royal Decree 1416/1994, of 25 June, which regulates the advertising of medicinal products for human use, in Royal Decree 1907/1996, of 2 August, on advertising and commercial promotion of products, activities or services with purported health purposes, and in Law 34/1988, of 11 November, General Advertising.*
5. *In all cases, in order to make public the general results of the investigations once concluded, the guidelines of the European Commission and, where appropriate, the instructions of the Spanish Agency for Medicines and Health Products will be followed.*
6. *Where a sub-study of a clinical trial is completed at a later date than the remainder of the trial, it shall be necessary for the summary of its results to be published in the year following its completion, without delay in the submission of the results of the remainder of the trial."*

# ANNEX I. LIST OF PARTICIPATING CENTERS (updated to July 2023)

| **CENTER** | **PRINCIPAL INVESTIGATOR** |
| --- | --- |
| Virgen del Rocío University Hospital (Seville) | Elena Zapata Arriaza |
| Reina Sofía University Hospital (Córdoba) | Fernando Delgado Acosta |
| Virgen Macarena University Hospital (Seville) | Miguel Angel Gamero |
| Hospital Complex Torrecárdenas (Almería) | Laura Amaya Pascasio |
| Dr. Josep Trueta Hospital in Girona | Saima Bashir |
| University and Polytechnic Hospital of La Fe  (Valencia) | Irene Escudero Martinez |
| University Hospital of Cruces (Bilbao) | Maria del Mar Freijó |
| University Hospital of Badajoz | Jose Maria Ramirez Moreno |
| University Hospital Complex A Coruña | Sonia Mosteiro |
| Hospital Clínico Universitario Virgen de la  Arrixaca. Murcia | Jose Diaz Perez |
| Hospital Ramón y Cajal | Isabel Bermudez |
| Hospital Germans Trias i Pujol | Alejandro Bustamante Ranger |
| General University Hospital of Alicante | Jose Gallego |

**ANNEX II. DATA SHEET Tirofiban (AGRASTAT*0.05 mg/ml solution for infusion)**


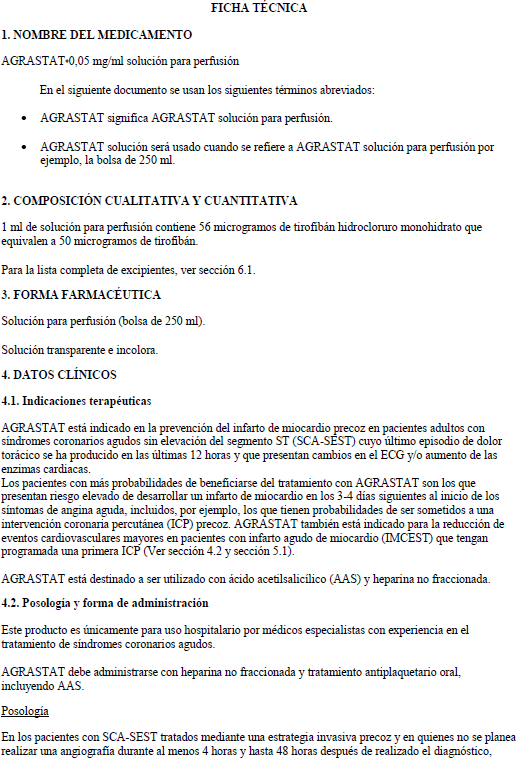


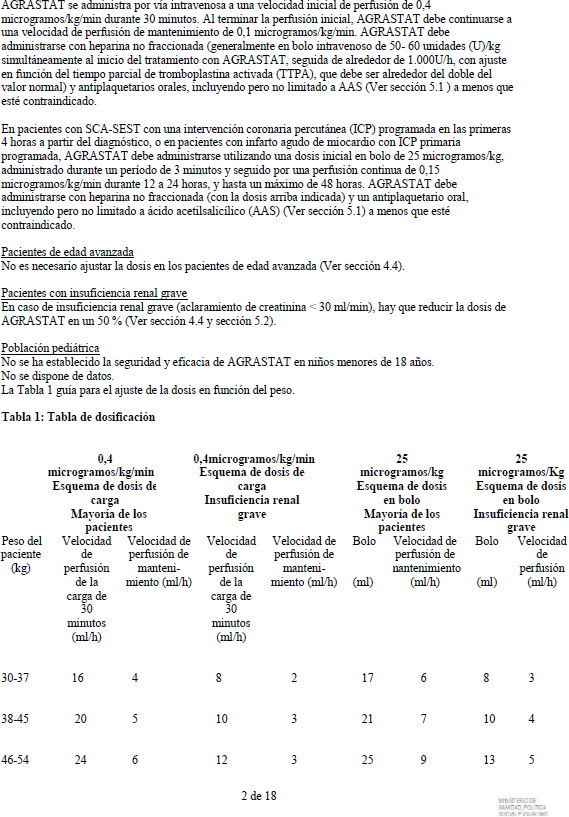


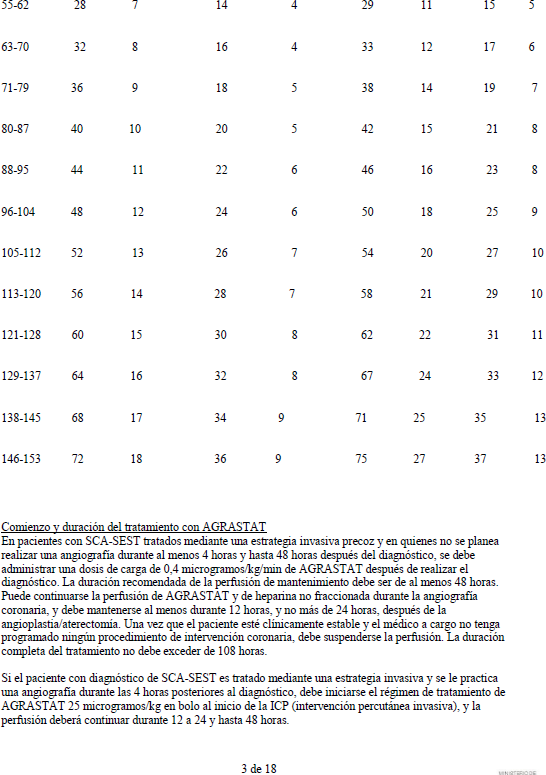


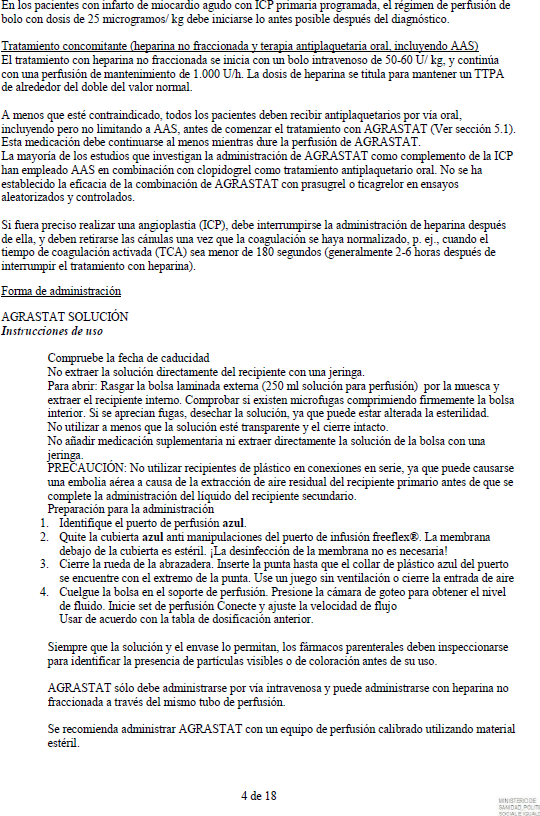


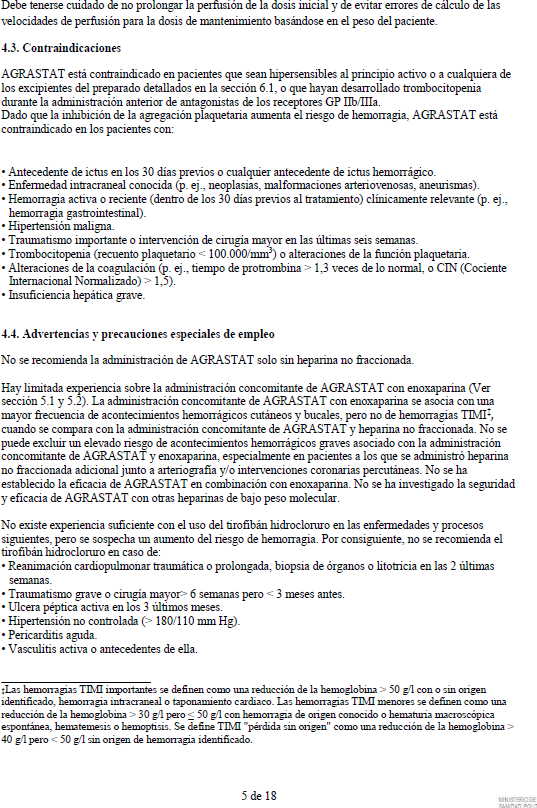


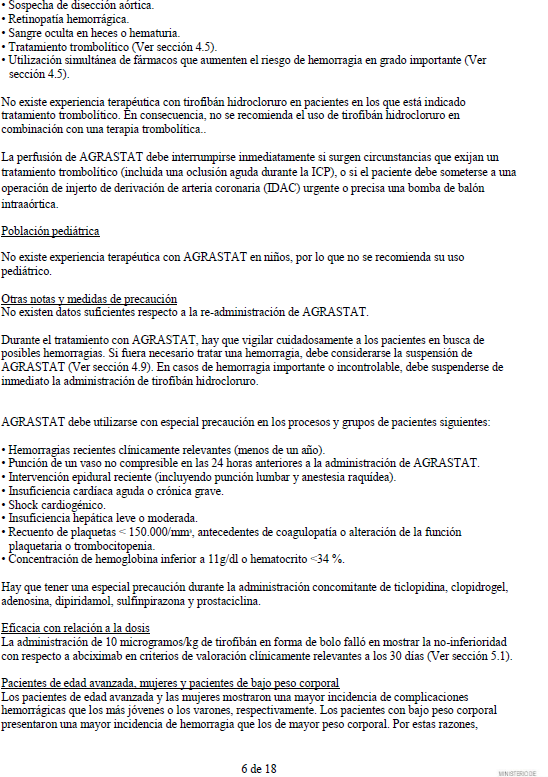


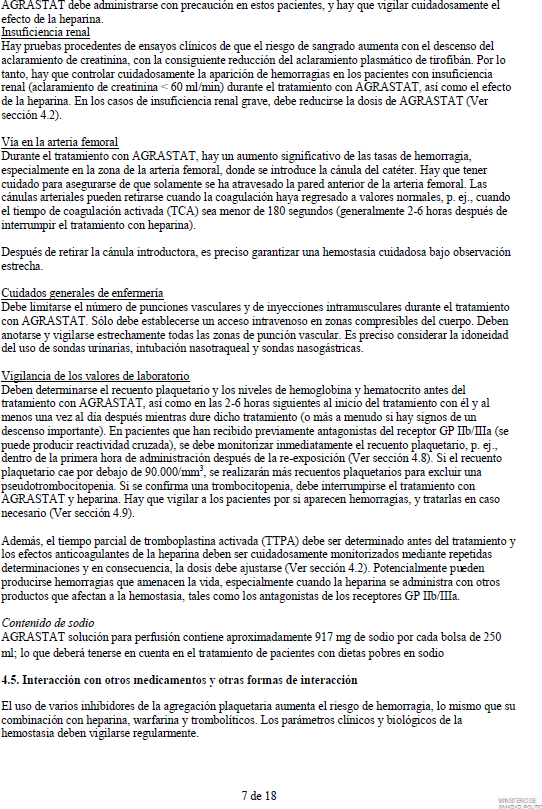


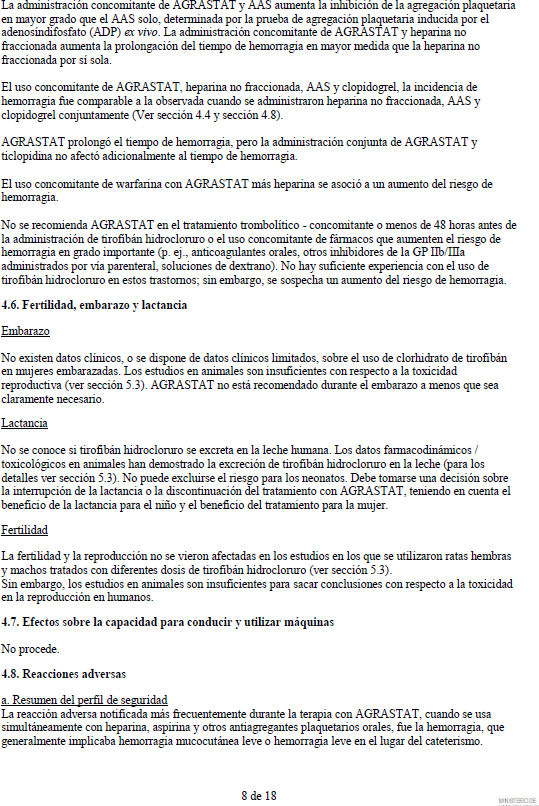


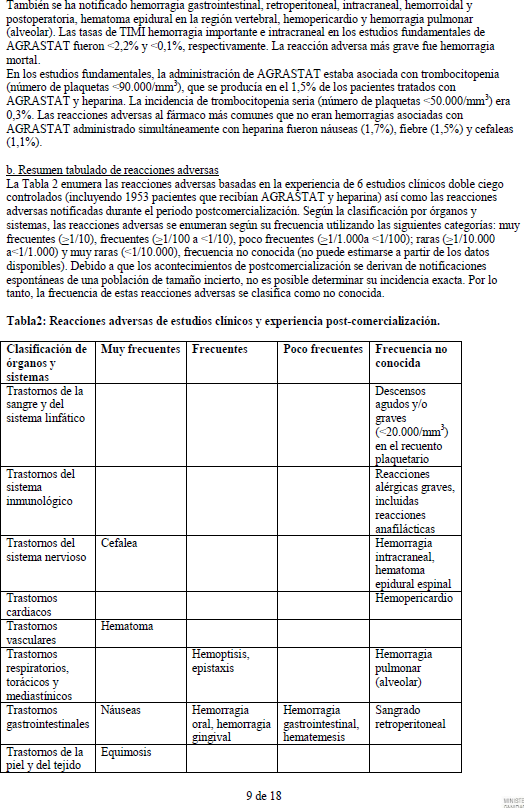


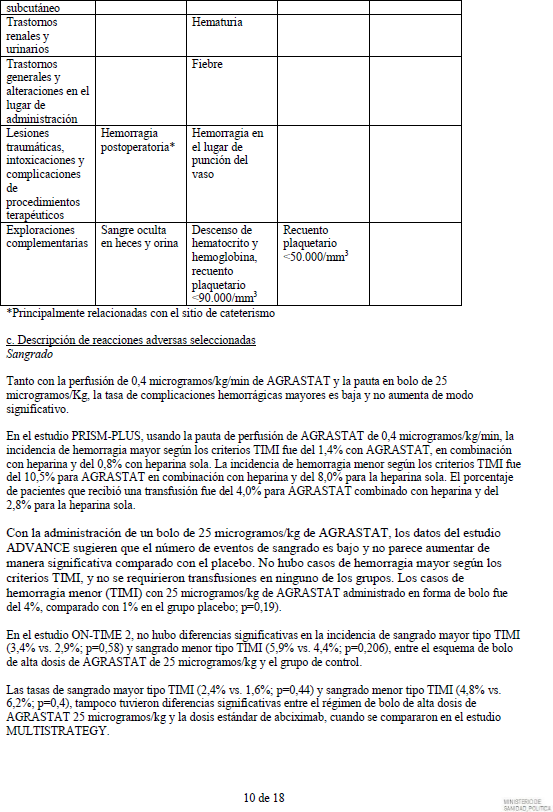


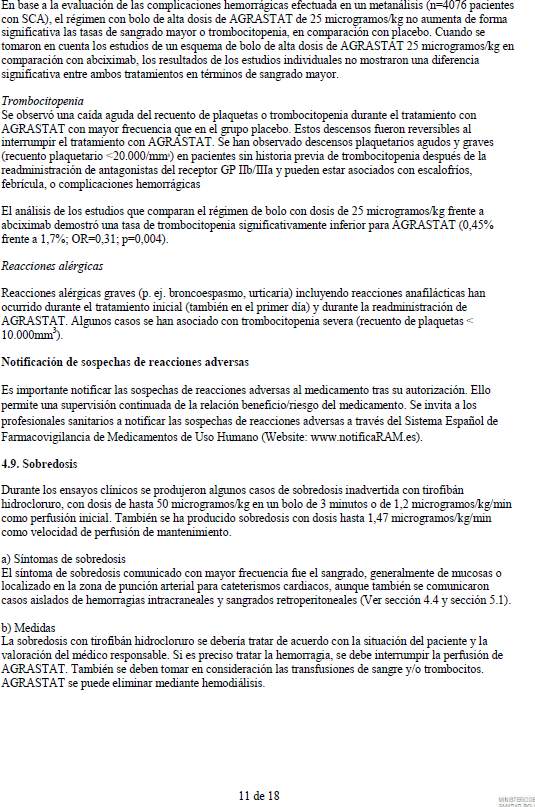


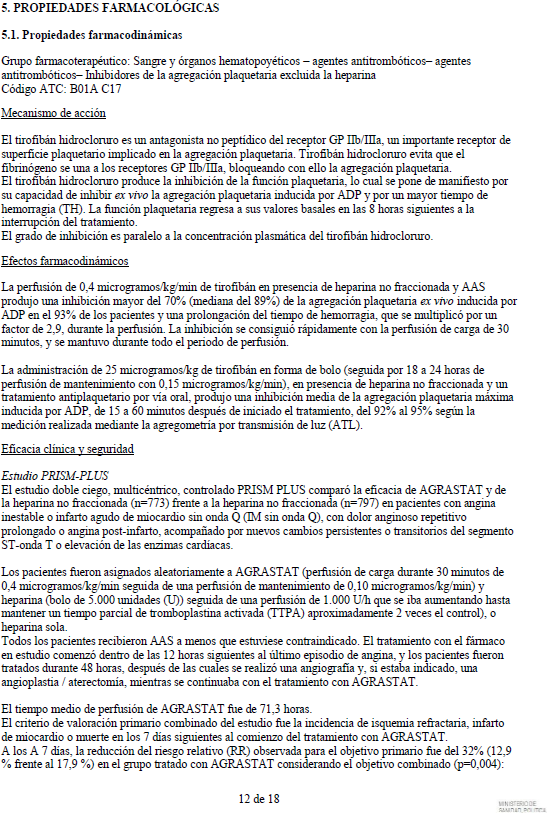


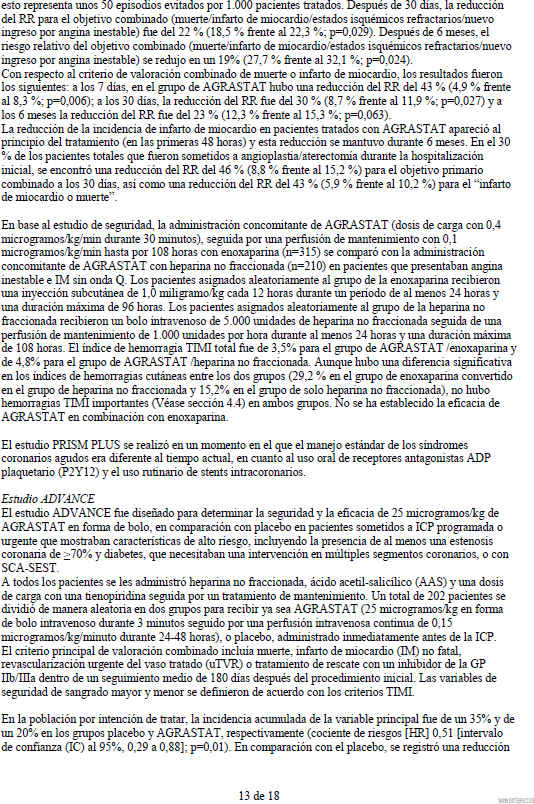


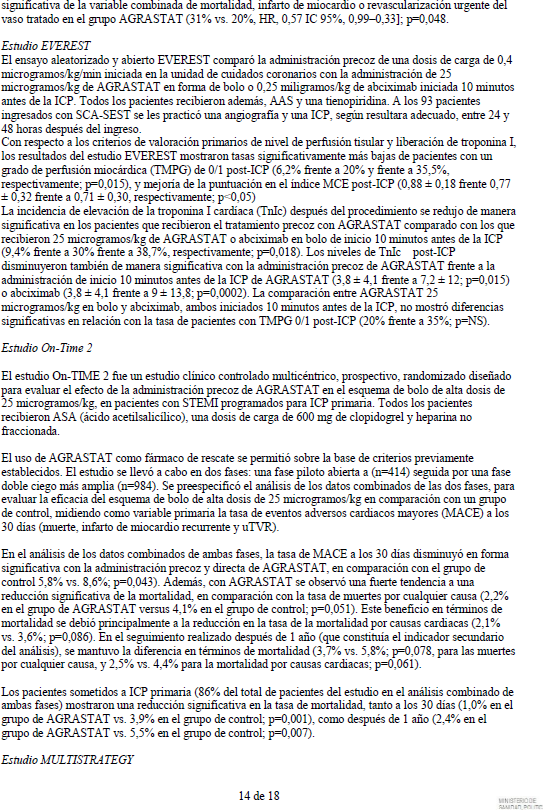


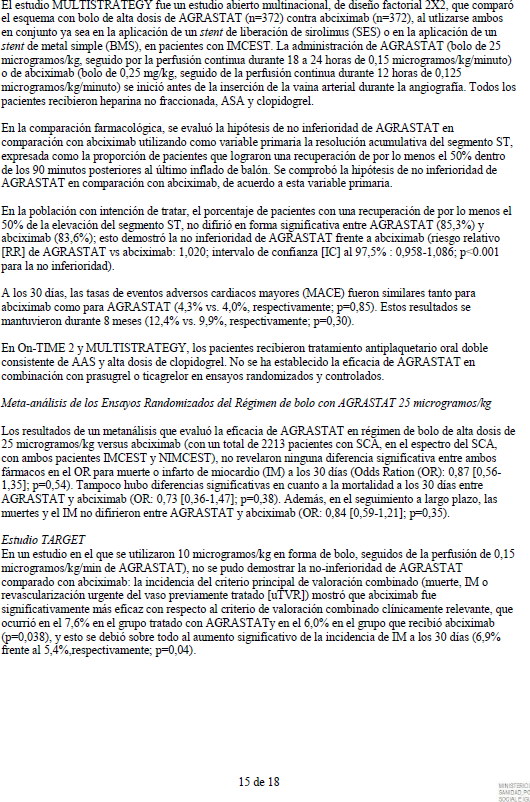


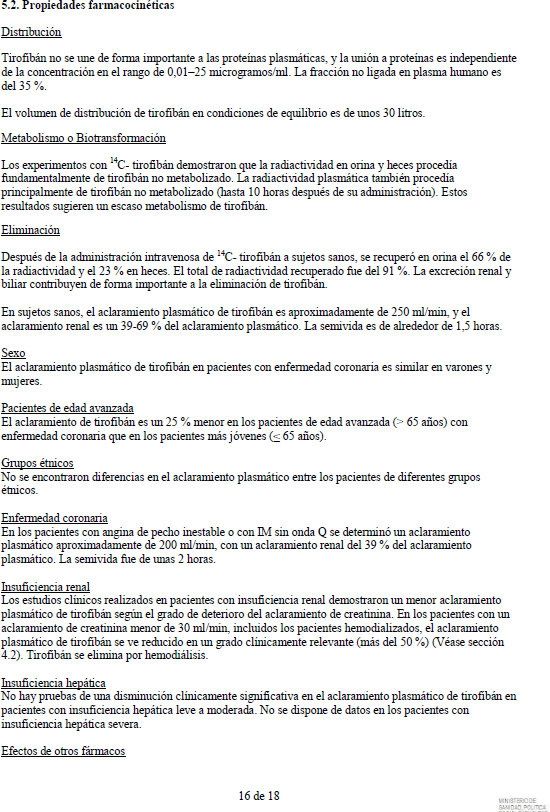


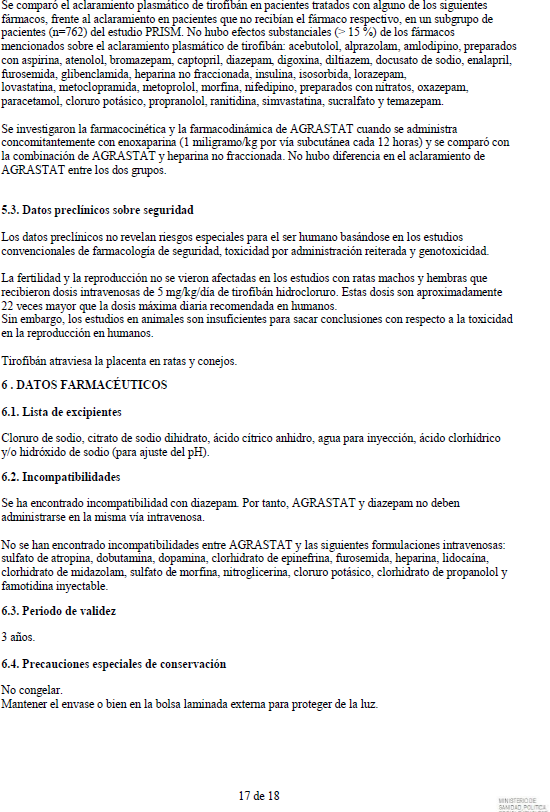


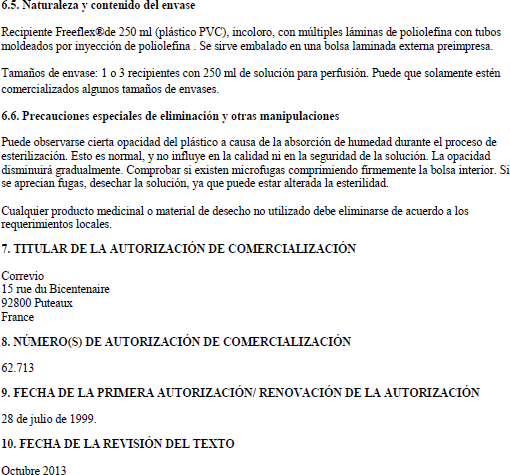


# ANNEX III. DATA SHEET Acetylsalicylic Acid Injection (INYESPRIN®)


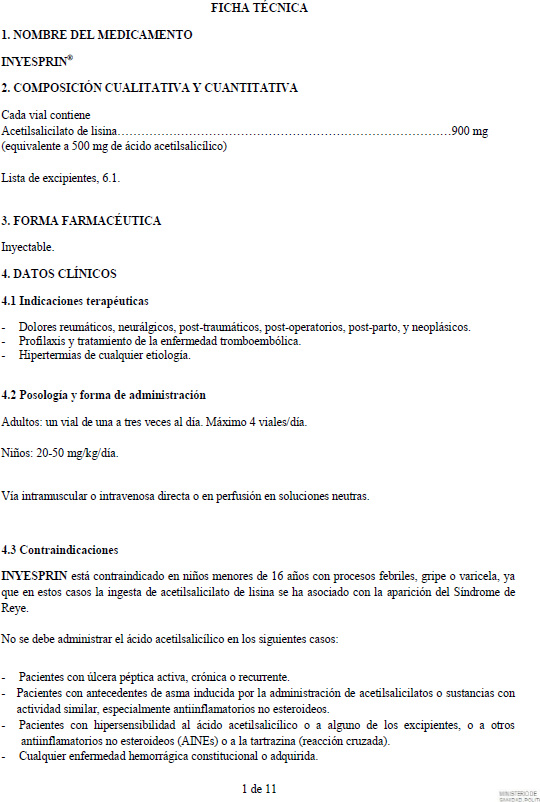


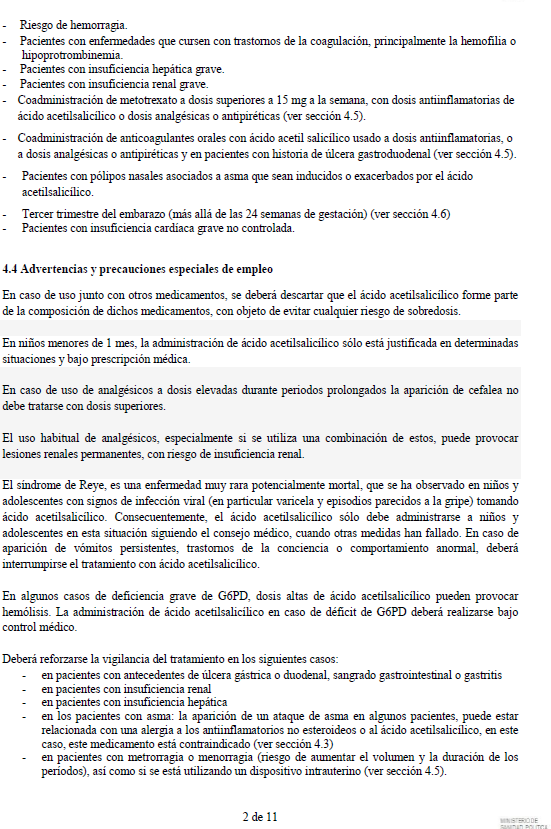


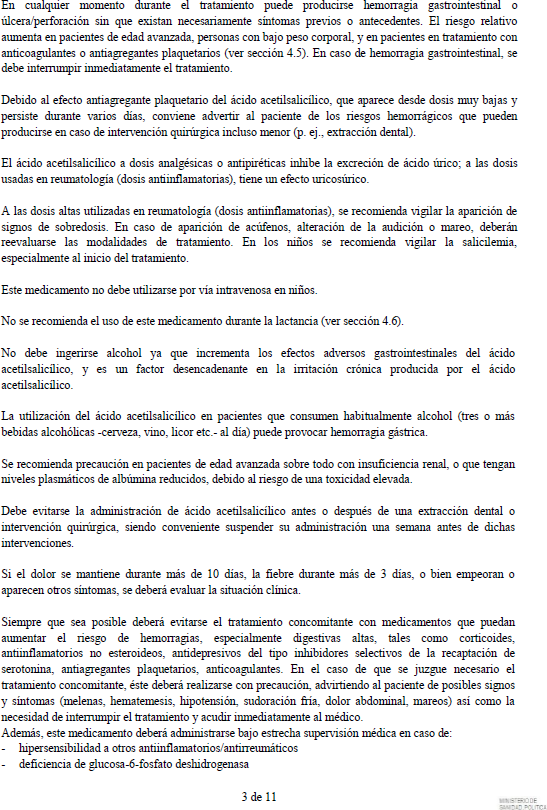


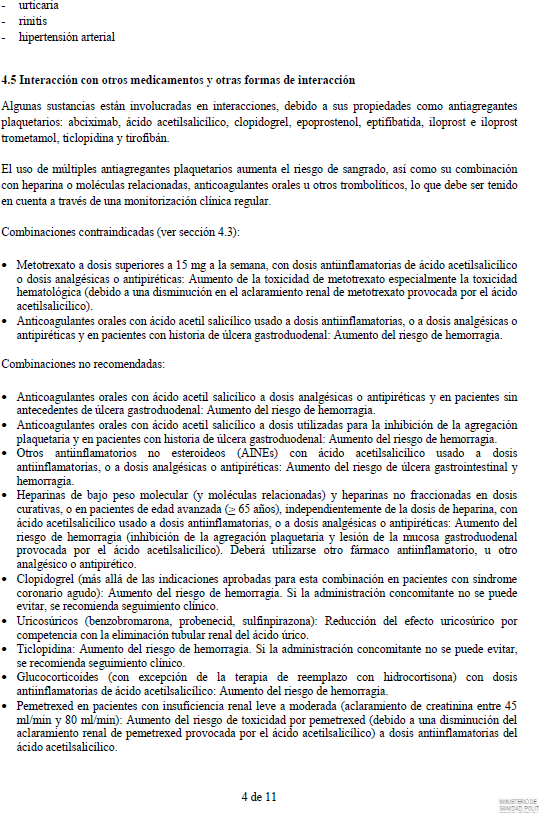


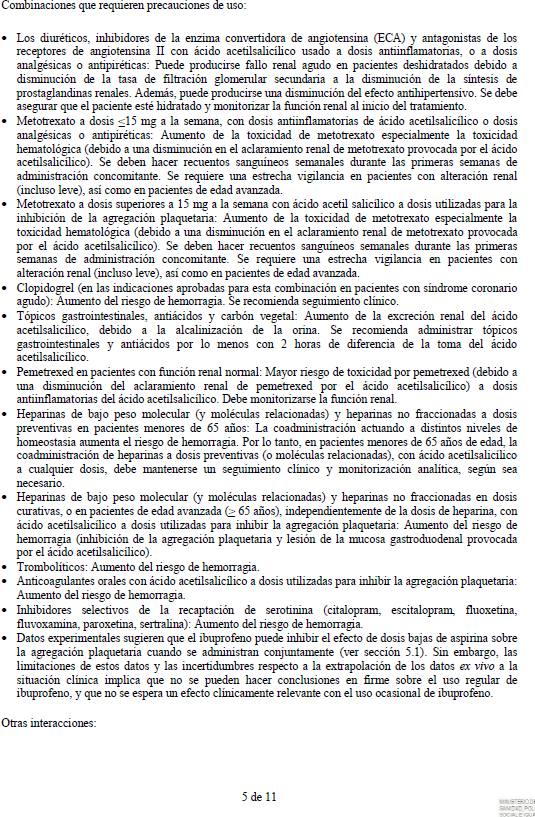


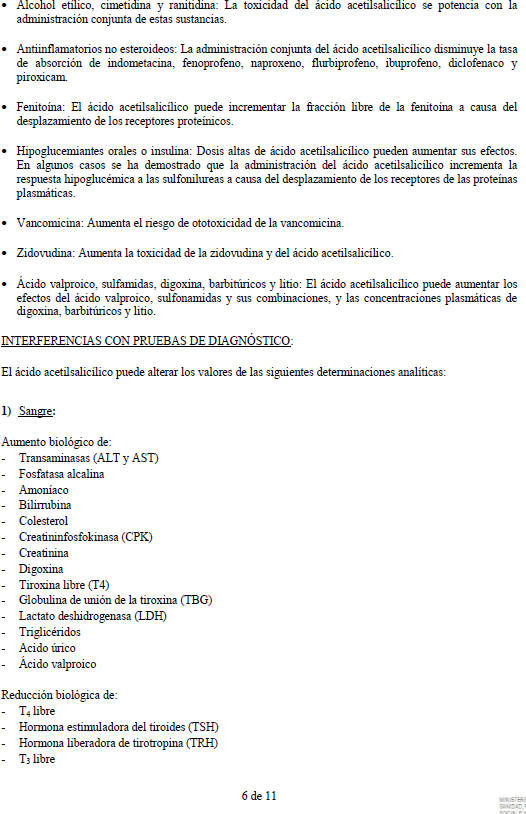


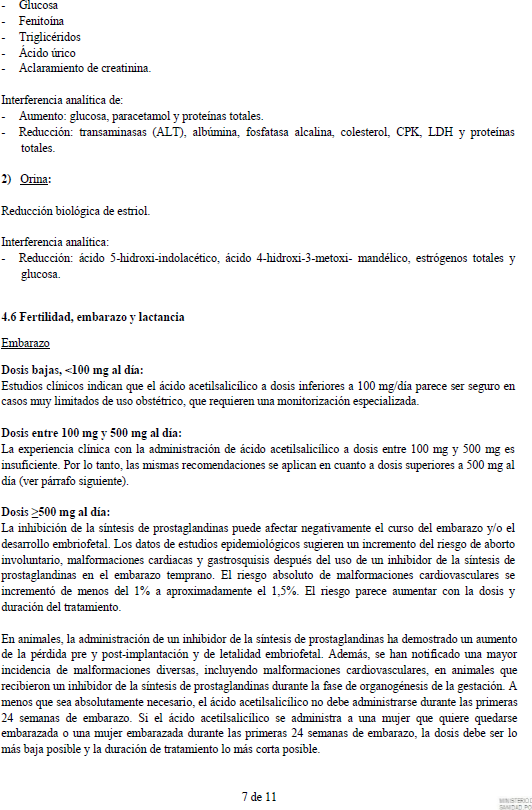


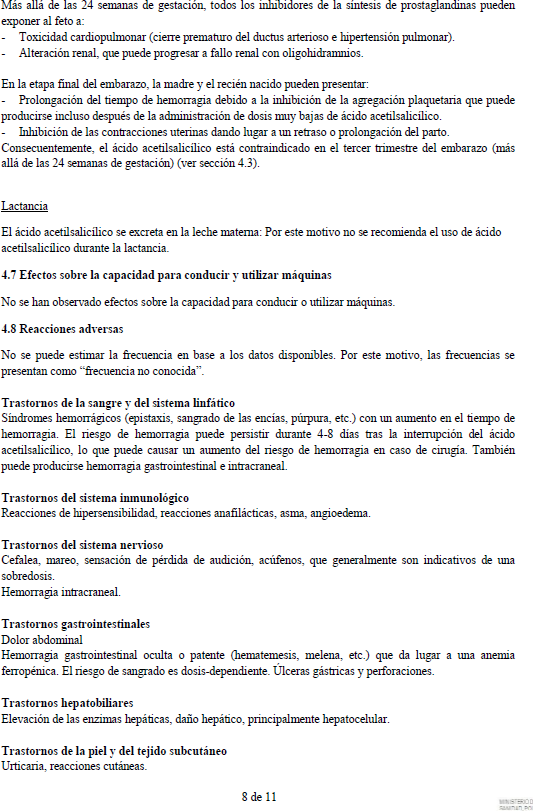


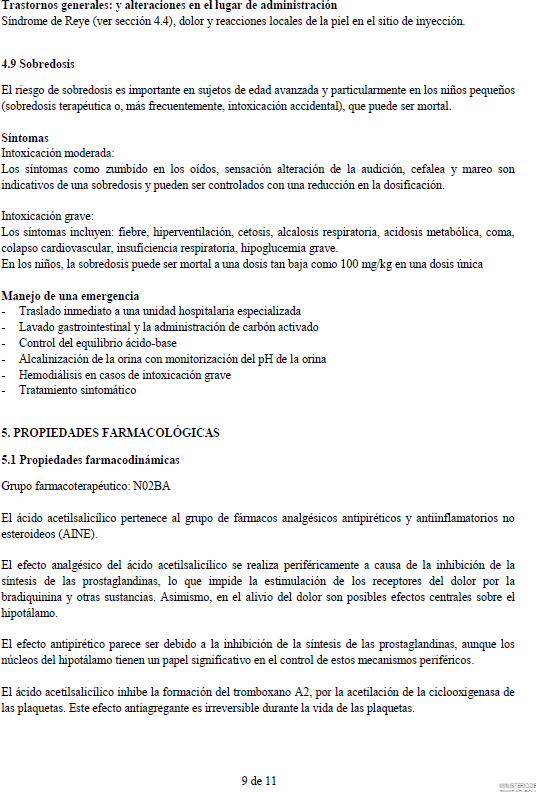


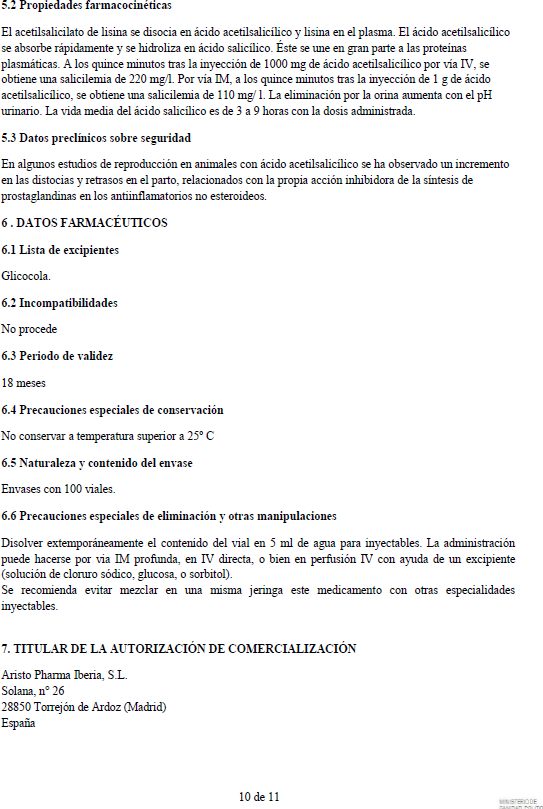


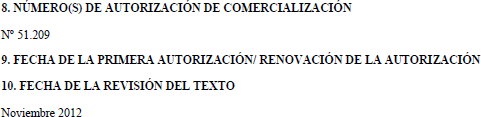


**ANNEX IV. BIBLIOGRAPHY**

**1.** Assis Z, Menon BK, Goyal M, Demchuk AM, Shankar J, Rempel JL, et al. Acute ischemic stroke with tandem lesions: technical endovascular management and clinical outcomes from the ESCAPE trial. J Neurointerv Surg. 2018;10(5):429-33.

2. Kim YS, Garami Z, Mikulik R, Molina CA, Alexandrov AV, CLOTBUST Collaborators. Early recanalization rates and clinical outcomes in patients with tandem internal carotid artery/middle cerebral artery occlusion and isolated middle cerebral artery occlusion. Stroke. 2005;36(4):869-71.

3. Anadani M, Spiotta AM, Alawieh A, Turjman F, Piotin M, Haussen DC, et al. Emergent Carotid Stenting Plus Thrombectomy After Thrombolysis in Tandem Strokes: Analysis of the TITAN Registry. Stroke. 2019;50(8):2250-2.

4. Zapata-Arriaza E, de Albóniga-Chindurza A, Ortega-Quintanilla J, Escudero-Martínez I, Moniche F, Medina-Rodríguez M, et al. Clinical Outcomes of Mechanical Thrombectomy in Stroke Tandem Lesions According to Intracranial Occlusion Location. J Stroke. 2021;23(1):124-7.

5. Powers WJ, Rabinstein AA, Ackerson T, Adeoye OM, Bambakidis NC, Becker K, et al. Guidelines for the Early Management of Patients With Acute Ischemic Stroke: 2019 Update to the 2018 Guidelines for the Early Management of Acute Ischemic Stroke: A Guideline for Healthcare Professionals From the American Heart Association/American Stroke Association. Stroke. 2019;50(12):e344-418.

6. Saver JL, Goyal M, Bonafe A, Diener HC, Levy EI, Pereira VM, et al. Stent-retriever thrombectomy after intravenous t-PA vs. t-PA alone in stroke. N Engl J Med. 2015;372(24):2285-95.

7. Campbell BCV, Mitchell PJ, Kleinig TJ, Dewey HM, Churilov L, Yassi N, et al. Endovascular therapy for ischemic stroke with perfusion-imaging selection. N Engl J Med. 2015;372(11):1009-18.

8. Berkhemer OA, Fransen PSS, Beumer D, van den Berg LA, Lingsma HF, Yoo AJ, et al. A randomized trial of intraarterial treatment for acute ischemic stroke. N Engl J Med. 2015;372(1):11-20.

9. Jovin TG, Chamorro A, Cobo E, de Miquel MA, Molina CA, Rovira A, et al. Thrombectomy within 8 hours after symptom onset in ischemic stroke. N Engl J Med.;372(24):2296-306.

10. Goyal M, Demchuk AM, Menon BK, Eesa M, Rempel JL, Thornton J, et al. Randomized assessment of rapid endovascular treatment of ischemic stroke. N Engl J Med. 2015;372(11):1019-30.

11. Goyal M, Menon BK, van Zwam WH, Dippel DWJ, Mitchell PJ, Demchuk AM, et al. Endovascular thrombectomy after large-vessel ischaemic stroke: a meta-analysis of individual patient data from five randomised trials. Lancet. 2016;387(10029):1723-31.

12. Poppe AY, Jacquin G, Roy D, Stapf C, Derex L. Tandem Carotid Lesions in Acute Ischemic Stroke: Mechanisms, Therapeutic Challenges, and Future Directions. AJNR Am J Neuroradiol. 2020;41(7):1142-8.

13. Wilson MP, Murad MH, Krings T, Pereira VM, O’Kelly C, Rempel J, et al. Management of tandem occlusions in acute ischemic stroke - intracranial versus extracranial first and extracranial stenting versus angioplasty alone: a systematic review and meta-analysis. J Neurointerv Surg. 2018;10(8):721-8.

14. Haussen DC, Turjman F, Piotin M, Labreuche J, Steglich-Arnholm H, Holtmannspötter M, et al. Head or Neck First? Speed and Rates of Reperfusion in Thrombectomy for Tandem Large Vessel Occlusion Strokes. Interv Neurol. 2020;8(2-6):92-100.

15. Zapata-Arriaza E, Moniche F, González A, Bustamante A, Escudero-Martínez I, De la Torre Laviana FJ, et al. Predictors of Restenosis Following Carotid Angioplasty and Stenting. Stroke. 2016;47(8):2144-7.

16. Gory B, Haussen DC, Piotin M, Steglich-Arnholm H, Holtmannspötter M, Labreuche J, et al. Impact of intravenous thrombolysis and emergent carotid stenting on reperfusion and clinical outcomes in patients with acute stroke with tandem lesion treated with thrombectomy: a collaborative pooled analysis. Eur J Neurol. 2018;25(9):1115-20.

17. Sadeh-Gonik U, Tau N, Friehmann T, Bracard S, Anxionnat R, Derelle AL, et al. Thrombectomy outcomes for acute stroke patients with anterior circulation tandem lesions: a clinical registry and an update of a systematic review with meta-analysis. Eur J Neurol. 2018;25(4):693-700.

18. Eker OF, Bühlmann M, Dargazanli C, Kaesmacher J, Mourand I, Gralla J, et al. Endovascular Treatment of Atherosclerotic Tandem Occlusions in Anterior Circulation Stroke: Technical Aspects and Complications Compared to Isolated Intracranial Occlusions. Front Neurol. 2018;9:1046.

19. Zhu F, Bracard S, Anxionnat R, Derelle AL, Tonnelet R, Liao L, et al. Impact of Emergent Cervical Carotid Stenting in Tandem Occlusion Strokes Treated by Thrombectomy: A Review of the TITAN Collaboration. Front Neurol. 2019;10:206.

20. Renú A, Blasco J, Laredo C, Llull L, Urra X, Obach V, et al. Carotid stent occlusion after emergent stenting in acute ischemic stroke: Incidence, predictors and clinical relevance. Atherosclerosis. 2020;313:8-13.

21. Steglich-Arnholm H, Holtmannspötter M, Kondziella D, Wagner A, Stavngaard T, Cronqvist ME, et al. Thrombectomy assisted by carotid stenting in acute ischemic stroke management: benefits and harms. J Neurol. 2015;262(12):2668-75.

22. Wallocha M, Chapot R, Nordmeyer H, Fiehler J, Weber R, Stracke CP. Treatment Methods and Early Neurologic Improvement After Endovascular Treatment of Tandem Occlusions in Acute Ischemic Stroke. Front Neurol. 2019;10:127.

23. Kamarova M, Baig S, Patel H, Monks K, Wasay M, Ali A, et al. Antiplatelet Use in Ischemic Stroke. Ann Pharmacother. 2022;56(10):1159-73.

24. Bhatia K, Ladd LM, Carr KH, Di Napoli M, Saver JL, McCullough LD, et al. Contemporary Antiplatelet and Anticoagulant Therapies for Secondary Stroke Prevention: A Narrative Review of Current Literature and Guidelines. Curr Neurol Neurosci Rep. 2023;23(5):235-62.

25. Zinkstok SM, Roos YB, ARTIS investigators. Early administration of aspirin in patients treated with alteplase for acute ischaemic stroke: a randomised controlled trial. Lancet. 25 de agosto de 2012;380(9843):731-7.

26. Zhao W, Che R, Shang S, Wu C, Li C, Wu L, et al. Low-Dose Tirofiban Improves Functional Outcome in Acute Ischemic Stroke Patients Treated With Endovascular Thrombectomy. Stroke. 2017;48(12):3289-94.

27. Cheng Z, Geng X, Gao J, Hussain M, Moon SJ, Du H, et al. Intravenous Administration of Standard Dose Tirofiban after Mechanical Arterial Recanalization is Safe and Relatively Effective in Acute Ischemic Stroke. Aging Dis. 2019;10(5):1049-57.

28. Pan X, Zheng D, Zheng Y, Chan PWL, Lin Y, Zou J, et al. Safety and efficacy of tirofiban combined with endovascular treatment in acute ischaemic stroke. Eur J Neurol. 2019;26(8):1105-10.

29. Paciaroni M, Bogousslavsky J. Antithrombotic therapy in carotid artery stenosis: an update. Eur Neurol. 2015;73(1-2):51-6.

30. Borchert RJ, Simonato D, R Hickman C, Fuschi M, Thibault L, Henkes H, et al. P2Y12 inhibitors for the neurointerventionalist. Interv Neuroradiol. 2022;28(1):92-103.

# ANNEX V. HELSINKI DECLARATION OF THE WORLD MEDICAL ASSOCIATION

**Ethical principles for medical research involving human subjects**

**Adopted by the 18th World Medical Assembly, Helsinki, Finland, June 1964 and amended by the**

**29th World Medical Assembly, Tokyo, Japan, October 1975 35th World Medical Assembly, Venice, Italy, October 1983 41st World Medical Assembly, Hong Kong, September 1989**

**48th General Assembly Somerset West, South Africa, October 1996 52nd General Assembly, Edinburgh, Scotland, October 2000**

**Note of Clarification to Paragraph 29, added by the WMA General Assembly, Washington 2002**

**Note of Clarification to Paragraph 30, added by the WMA General Assembly, Tokyo 2004 59th General Assembly, Seoul, Korea, October 2008**

**64th General Assembly, Fortaleza, Brazil, October 2013**

1. **INTRODUCTION**
   1. The World Medical Association (WMA) has promulgated the Declaration of Helsinki as a proposal of ethical principles for medical research involving human subjects, including the investigation of identifiable human material and information.

The Declaration should be considered as a whole and a paragraph should be applied with consideration of all other relevant paragraphs.

- 1. In accordance with the WMA's mandate, the Declaration is intended primarily for physicians. The WMA urges others involved in medical research involving human subjects to adopt these principles.

1. **GENERAL PRINCIPLES**
2. The Declaration of Geneva of the World Medical Association binds the physician to the formula "to look after my patient's health solicitously and first and foremost," and the International Code of Medical Ethics states that: "The physician shall consider what is best for the patient when providing medical care."
3. The duty of the physician is to promote and ensure the health, welfare and rights of patients, including those involved in medical research. The knowledge and conscience of the physician must be subordinated to the performance of this duty.
4. The progress of medicine is based on research that must ultimately include studies on human subjects.
5. The main purpose of medical research involving human subjects is to understand the causes, course and effects of diseases and to improve preventive, diagnostic and therapeutic interventions (methods, procedures and treatments). Even the best proven interventions must be continually evaluated through research to be safe, efficient, effective, accessible, and of quality.
6. Medical research is subject to ethical standards that serve to promote and ensure respect for all human beings and to protect their health and individual rights.
7. Although the main objective of medical research is to generate new knowledge, this objective should never take precedence over the rights and interests of the person participating in the research.
8. In medical research, it is the duty of the physician to protect the life, health, dignity, integrity, right to self-determination, privacy and confidentiality of personal information of persons participating in research. The responsibility for the protection of persons taking part in research should always lie with a doctor or other health professional and never with the research participants, even if they have given their consent.
9. Physicians should consider ethical, legal and legal norms and standards for research involving human subjects in their own countries, as well as existing international norms and standards. A national or international ethical, legal or legal requirement must not be allowed to diminish or eliminate any protective measures for persons involved in research set out in this Declaration.
10. Medical research should be conducted in a manner that minimizes potential damage to the environment.
11. Medical research involving human subjects should be carried out only by persons with appropriate scientific and ethical education, training, and qualifications. Research on healthy patients or volunteers needs the supervision of a competent and appropriately qualified physician or other health professional.
12. Groups that are underrepresented in medical research should have appropriate access to participation in research.
13. A physician who combines medical research with medical care should involve his or her patients in the research only to the extent that this demonstrates justified potential preventive, diagnostic, or therapeutic value and if the physician has good reason to believe that participation in the study will not adversely affect the health of patients taking part in the research.
14. Appropriate compensation and treatment should be ensured for individuals who are harmed during their participation in research.
15. **RISKS, COSTS AND BENEFITS**
16. In the practice of medicine and medical research, most interventions involve some risks and costs.

Medical research involving human subjects should only be conducted when the importance of its objective outweighs the risk and costs to the person participating in the research.

1. All medical research involving human subjects should be preceded by a careful comparison of the risks and costs to the individuals and groups participating in the research, compared to the expected benefits to them and to other individuals or groups affected by the disease being investigated.

Measures should be implemented to minimize risks. Risks must be continuously monitored, evaluated and documented by the investigator.

1. Physicians should not be involved in research studies involving human subjects unless they are sure that the risks have been adequately assessed and that they can be satisfactorily addressed.

When the risks involved outweigh the expected benefits or if there is conclusive evidence of definitive results, physicians should consider whether to continue, modify, or immediately discontinue the study.

1. **VULNERABLE GROUPS AND INDIVIDUALS**
2. Some groups and individuals under investigation are particularly vulnerable and may be more likely to suffer abuse or additional harm.

All groups and individuals must receive specific protection.

1. Medical research in a vulnerable group is only justified if the research responds to the health needs or priorities of this group and the research cannot be conducted in a non-vulnerable group. In addition, this group may benefit from knowledge, practices or interventions derived from research.
2. **SCIENTIFIC REQUIREMENTS AND RESEARCH PROTOCOLS**
3. Medical research involving human subjects should conform to generally accepted scientific principles and should be supported by a thorough knowledge of the scientific literature, other relevant sources of information, as well as sound laboratory experiments.

carried out and in animals, where appropriate. Care must also be taken of the welfare of the animals used in the experiments.

1. The design and method of any study involving human subjects must be clearly described and justified in a research protocol.

The protocol should always refer to relevant ethical considerations and should indicate how the principles set forth in this Declaration have been considered. The protocol should include information on funding, sponsors, institutional affiliations, potential conflicts of interest and incentives for study individuals, and information on stipulations for treating or compensating individuals who have suffered harm as a result of their participation in the research.

In clinical trials, the protocol should also describe appropriate arrangements for post-trial stipulations.

1. **RESEARCH ETHIC COMMITTEES**
2. The research protocol should be submitted, for consideration, comment, advice and approval to the relevant research ethic committee prior to commencing the study. This committee should be transparent in its functioning, independent of the investigator, sponsor or any other type of undue influence, and should be appropriately qualified. The committee should consider the laws and regulations in force in the country where the research is conducted, as well as existing international standards, but these should not be allowed to diminish or eliminate any of the protections for persons participating in the research set out in this Declaration.

The committee has the right to monitor ongoing trials. The investigator has an obligation to provide control information to the committee, especially on any serious adverse incident. No amendment should be made to the protocol without the consideration and approval of the committee. After the study ends, researchers must submit a final report to the committee with a summary of the results and conclusions of the study.

1. **PRIVACY AND CONFIDENTIALITY**
2. Every precaution should be taken to protect the privacy of the person involved in the investigation and the confidentiality of their personal information.
3. **INFORMED CONSENT**
4. The participation of persons capable of giving informed consent in medical research should be voluntary. Although it may be appropriate to consult family members or community leaders, no person capable of giving informed consent should be included in a study unless they freely agree.
5. In medical research involving human subjects capable of giving informed consent, each potential individual should be provided with adequate information about the objectives, methods, sources of funding, potential conflicts of interest, institutional affiliations of the investigator, calculated benefits, foreseeable risks and discomforts arising from the experiment, post-study stipulations, and all other relevant aspects of the research. The potential person should be informed of the right to participate or not to participate in the research and to withdraw his or her consent at any time, without exposing himself or herself to retaliation. Special attention should be paid to the specific information needs of each potential individual, as well as to the methods used to deliver the information.

After ensuring that the individual has understood the information, the physician or other appropriately qualified person should then request, preferably in writing, the individual's informed and voluntary consent. If consent cannot be given in writing, the process for achieving it must be formally documented and witnessed.

Everyone involved in medical research should have the option to be informed about the overall results of the study.

1. When seeking informed consent for participation in research, the physician should take special care when the potential individual is linked to him by a relationship of dependency or if he consents under pressure. In such a situation, informed consent must be requested by an appropriately qualified person who has nothing to do with that relationship.
2. When the potential individual is unable to give informed consent, the physician should seek informed consent from the legal representative. These persons should not be included in research that has no potential of benefit to them, unless it aims to promote the health of the group represented by the potential individual and this research cannot be conducted on persons capable of giving informed consent and the research involves only minimal risk and cost.
3. If a potential individual participating in the research considered incapable of giving informed consent is able to give his assent to participate or not to participate in the research, the physician must ask for it, in addition to the consent of the legal representative. The potential individual's disagreement must be respected.
4. Research on individuals who are not physically or mentally capable of giving consent, for example unconscious patients, can be conducted only if the physical/mental condition that precludes giving informed consent is a necessary characteristic of the investigated group. In these circumstances, the doctor must ask for informed consent from the legal representative. If such a representative is not available and if the research cannot be delayed, the study may be conducted without informed consent, provided that the specific reasons for including individuals with a disease that does not allow them to give informed consent have been stipulated in the research protocol and the study has been approved by a research ethics committee. Consent to remain in the research should be obtained as soon as possible from the individual or a legal representative.
5. The physician should fully inform the patient of the aspects of care that are related to the investigation. The patient's refusal to participate in research or his or her decision to withdraw should never adversely affect the doctor-patient relationship.
6. For medical research using identifiable human material or data, such as research on material or data contained in biobanks or similar repositories, the physician should seek informed consent for collection, analysis, storage and reuse. There may be exceptional situations in which it will be impossible or impracticable to obtain consent for such research. In this situation, research can only be conducted after being considered and approved by a research Ethics committee.
7. **USE OF PLACEBO**
8. The potential benefits, risks, costs and efficacy of any new intervention should be assessed by comparing it with the best proven interventions, except in the following circumstances:
   - When there is no proven intervention, the use of a placebo, or no intervention, is acceptable or
   - When for methodological, scientific and pressing reasons, the use of any intervention less effective than the best proven, the use of a placebo or no intervention is necessary to determine the efficacy and safety of an intervention.
   - and patients who receive any intervention less effective than the best proven intervention, placebo or no intervention, will not face additional risks, serious adverse effects or irreversible harm as a result of not receiving the best proven intervention.

Great care must be taken to avoid abusing this option.

1. **POST-TRIAL STIPULATIONS**
2. Prior to the clinical trial, sponsors, investigators, and host country governments should provide post-trial access to all participants who still need an intervention that has been identified as beneficial in the trial. This information must also be provided to participants during the informed consent process.
3. **REGISTRATION AND PUBLICATION OF RESEARCH AND DISSEMINATION OF RESULTS**
4. All research studies involving human subjects must be registered in a publicly available database before accepting the first person.
5. Researchers, authors, sponsors, directors and editors all have ethical obligations regarding the publication and dissemination of their research results. Researchers have a duty to make the results of their research on human subjects publicly available and are responsible for the completeness and accuracy of their reports. All parties must accept ethical standards of information delivery. Both negative and positive results must be published or otherwise they must be publicly available. The publication should cite the source of funding, institutional affiliations and conflicts of interest. Research reports that do not adhere to the principles described in this Statement should not be accepted for publication.
6. **INTERVENTIONS NOT PROVEN IN CLINICAL PRACTICE**
7. When proven interventions in the care of a patient do not exist or other known interventions have proved ineffective, the physician, after seeking expert advice, with the informed consent of the patient or an authorized legal representative, may allow himself to use unproven interventions, if, in his opinion, this gives some hope of saving life, restore health or alleviate suffering. Such interventions should be investigated further in order to assess their safety and efficacy. In all cases, such new information should be recorded and, where appropriate, made available to the public.

# ANNEX VI. CONCOMITANT MEDICATION

**CLOPIDOGREL**

Directions

Secondary prevention of cerebral atherothrombotic events. Its current indication in neurovascular pathology includes transient ischemic attack (TIA) or non-cardioembolic stroke minor, symptomatic intracranial stenosis and in case of carotid revascularization due to significant symptomatic stenosis requiring the placement of cervical stents.

Presentation

Clopidogrel Ur 75 mg film-coated tablets Loading and maintenance dose.

Treatment with Clopidogrel should be initiated with a single loading dose of 300 mg and subsequently continued with a dose of 75 mg once daily.

Contraindications

Hypersensitivity to the active substance or to some of the excipients, severe hepatic impairment, active pathological bleeding.

**LABETALOL**

Directions

Urgent management of high blood pressure Presentation

The form comes as Trandate ® 1 ampoule of 100 mg in 20 ml (5mg/ml). Bowling

Bolus of 10mg (2ml) undiluted to pass in 1 minute. If necessary, repeat the same dose every 5-10 min. If with 3 boluses TA control is not achieved, start infusion.

Perfusion

Dilute 5 ampoules (100ml in total) of Trandate (500mg) in SSF to complete a total of 250cc.

1 ml = 2 mg labetalol

LABETALOL

(1000 mg in 250 cc)

1mg/min-30 cc/h

2 mg/min -60 cc/h

3 mg/min -90 cc/h

4 mg/min -120 cc/h

Start infusion at 1 mg/min = 30 cc/h until blood pressure control. Side effects

Vomiting, postural hypotension, nausea, vertigo Precautions

If HR < 60-70 bpm, it is preferable to use other antihypertensives. In diabetics and bronchopaths, other options are preferable.

Remarks

BP control every 15 min to avoid hypotension. Heart rate monitoring.

Contraindications: asthma bronchial arrhythmia cardiac blockade atrioventricular, heart failure,

Caution in diabetics and bronchopaths.

**URAPIDILO**

Directions

Urgent management of arterial hypertension Form of presentation.

1 ampoule of 50mg/10ml. Elgadil Bowling

25 mg (1/2 ampoule) in 50cc.

If it does not go down BP repeat bolus up to 3 times every 5 min, if it is not stabilized start infusion. Perfusion

Depending on volume management, dilute 5 ampoules of Urapidilo (50 cc) + 200 cc of S. Total physiological of 250 cc (1ml = 1mg) Or dilute 5 ampoules of Urapidilo (50 cc) + 450 cc of S. Physiological up to a total of 500cc (1 ml = 0.5mg). Start the infusion at 5mg/h (5ml and 10ml respectively) and gradually increase until blood pressure levels normalize. Maximum dose of 90mg/h

Starting dose

5-10 ml/h = 5-10ml/h. It will go up (from 10 to 10) according to tolerance and response until the blood pressure figures normalize.

Maximum 90 ml/H = 90mg/H Contraindications

Hypersensitivity to the active substance or to any of the excipients Side effects

Hypotension, headache, dizziness, sweating, nausea, bradycardia. Remarks

The infusion of urapidil will be a maximum of 7 days.

**CLEVIDIPINE**

Directions

Indicated for rapid reduction of refractory blood pressure Form of presentation

Cleviprex 0.5 mg/ml emulsion for injection, vials 50mg/100 ml Infusion

Initiate intravenous infusion of clevidipine at 4 ml/h (2 mg/h); The dose can be doubled every 90 seconds until the desired blood pressure range is reached.

Usual control range at 8-12 ml/h (4-6 mg/h); Max dose 64 ml/h (32 mg/h). It is recommended not to administer more than 1000 ml (500 mg) in 24h.

Contraindications

Clevidipine should not be used in patients with defects in lipid metabolism such as pathological hyperlipidemia, lipoid nephrosis or acute pancreatitis if accompanied by hyperlipidaemia.

**METOCLOPRAMIDE**

Directions

Prevention and symptomatic treatment of nausea and vomiting.

Form of presentation

Primperan 10 mg/2ml solution for injection. Bowling

Intravenously, the dose is 10 mg in a slow bolus (at least for 3 minutes). The maximum recommended daily dose is 30 mg or 0.5 mg/kg body weight.

Contraindications

Hypersensitivity to the active substance or to any of the excipients, gastrointestinal bleeding, pheochromocytoma, history of drug-induced tardive dyskinesia, Parkinson's disease, taking Levodopa or dopamine agonists, known history of methemoglobinemia with metoclopramide or NADH cytochrome b5 reductase deficiency.

Remarks

Extrapyramidal disorders may occur, particularly in young adults, and/or when high doses are used.

**ONDANSETRON**

Directions

Prevention and symptomatic treatment of nausea and vomiting. Form of presentation

Yatrox 4 and 8 mg solution for injection Bolus

A single dose of 4 mg may be given through a slow intravenous route. Contraindications

Hypersensitivity to the active substance or to any of the excipients.

**MIDAZOLAM**

Directions

Short-acting sleep inducer indicated for conscious sedation before and during diagnostic or therapeutic procedures with or without local anesthesia

Form of Presentation

Ampoules of 15 mg/3 ml, 5 mg/5 ml and 50 mg/10 ml, for IV administration Method of administration

Bolus administration of 3-5mg IV direct bolus Contraindications

Hypersensitivity to benzodiazepines.

Use of this medicine for conscious sedation of patients with severe respiratory failure or acute respiratory depression

**FENTANYL**

Directions

An opioid analgesic used in premedication, induction, maintenance, and for postoperative pain control. Analgesic supplement in general or regional anesthesia.

Presentation form Ampoules of 0.15mg/3ml. Method of administration

Bolus of 25-50 mcg (0.25-0.50 mg) for perioperative-postoperative analgesia. Contraindications

Hypersensitivity to the active substance(s)

Respiratory depression without mechanical ventilation

Concomitant administration with monoamine oxidase inhibitors or within 2 weeks after discontinuation of use.

Known intolerance to fentanyl citrate or other morphinomimetics.

**EPHEDRINE**

Directions

Treatment of Hypotension Presentation

Ampoules with 3mg/ml Method of administration:

Administration of intravenous slow bolus in boluses of 3-6mg repeated every 3-4 minutes depending on needs up to a maximum of 30mg.

Contraindications

Hypersensitivity to the active substance(s) Combination with phenylephrine, pseudoephedrine and methylphenidate Combination with MAOIs

**ATROPINE**

Directions:

Sinus bradycardia. Presentation Ampoule of 1 mg.

Method of administration:

1 mg bolus. 0.5-1 mg may be repeated and may be repeated every 5 minutes up to a maximum dose of 0.04 mg/kg. Immediate effect and short duration.

Adverse effects:

Anticholinergic effects (hypotension, tachycardia, urinary retention, blurred vision, constipation, dermatitis, headache, ataxia and disorientation). Antidote: physostigmine.

Contraindications

Contraindications: SVT and ventricular. Precaution in patients with glaucoma and coronary artery disease

**DOPAMINE**

Presentation

Ampoule of 200 mg in 5 cc. Perfusion

Preparation: 200 mg (1 ampoule, 5cc) in SG5% to complete 250 cc Solution with 800 μg/cc.

Pharmacodynamics

Inotropic and pressor, with dose-dependent effect: At low doses (~2 μg/kg/min) increases diuresis and natriuresis.

At medium doses (4-10 μg/kg/min) contractility and heart rate increase. At high doses (>10 μg/kg/min) the vasoconstrictor effect predominates

Directions

Its main indication is in severe heart failure (especially with decreased/unstable blood pressure) and as a first-line pressor drug. It's especially useful.

in patients with hypotension and bradycardia. Caution in patients with tachycardia because it increases the heart rate.

Side effects

Tachycardia, arrhythmias, myocardial ischemia, or increased pre-existing pulmonary hypertension


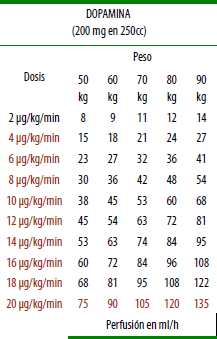


**AMIODARONE**

Form of presentation

Trangorex®; ampoule of 150 mg in 3 cc, tablet of 200 mg. Indications:

Tachyarrhythmias associated with Wolf-Parkinson-White syndrome, paroxysmal atrial fibrillation, prevention of recurrence of atrial fibrillation and flutter, supraventricular and ventricular tachycardias.

Method of administration

IV dose: A. Loading dose: bolus of 150-300 mg (5 mg/kg) in 50 ml 5% SG in 15-30 min.

B. Maintenance dose: – 1st regimen: 300 mg in 250 ml of 5% OS / 8h on the first day. As maintenance the next three days 300 mg, in 250 ml of 5% OS in 24h. Second regimen: Alternatively to the previous maintenance regimen, a maintenance infusion of 10 mg/kg in 24 hours may be used.

Administration should be discontinued or reduced if hypotension, bradycardia, or arrhythmia is controlled.

Oral dose: The oral starting dose is 200 mg/8h for 7 days and the maintenance dose is 100-200 mg/day.

Adverse effects:

Phlebitis, feeling of suffocation, sweating, nausea, hypotension, bradycardia. corneal deposits, photosensitization, bluish tint to the skin, paresthesias, tremor, ataxia, ventricular arrhythmias, thyroid dysfunction, severe interstitial pneumonitis.


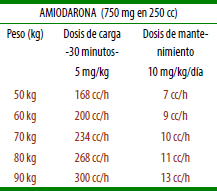


**DIGOXIN**

Presentation

Comp. of 0.25 mg and ampoule of 0.25 mg. Directions

Heart failure (especially useful in AF or SVT with rapid ventricular response), Flutter, Atrial fibrillation and atrial tachycardia with ectopic focus

Form of administration

Preparation: 0.25-0.50 mg i.v. bolus followed by 0.25 mg every 4-6 h. to complete 0.75-1 mg / 24h (only if necessary for frequency control). The oral maintenance dose is 0.25 mg/day.

Contraindications

Breast disease, advanced AV block, and Wolf-Parkinson-White syndrome. Caution in patients with hypoxia, hypokalemia, hypercalcemia, hypomagnesemia and renal insufficiency (Adjust dose according to renal function).

Adverse effects:

Vomiting, diarrhea, blurred vision, diplopia, headache, mental disorders, urticaria, gynecomastia, ventricular extrasystoles, SVT with AV block, AV block. Digitalis poisoning can simulate arrhythmias in which it is used as a therapeutic agent.

# ANNEX VII. Patient information sheet and informed consent.

| **STUDY TITLE** | Multicentre randomised clinical trial to assess efficacy and safety of tirofiban versus intravenous aspirin in patients with acute ischaemic stroke secondary to tandem lesion undergoing recanalisation therapy by endovascular therapy (ATILA Study). |
| --- | --- |
| **STUDY CODE** | ATTILA-stroke-2021 |
| **EudraCT NUMBER** | 2021-003874-30 |
| **PROMOTER** | Andalusian Public Foundation for the Management of Health Research in Seville (FISEVI). |
| **PRINCIPAL INVESTIGATOR** |  |
| **CENTER** |  |

Before signing this informed consent, carefully read the information provided below and ask the questions you consider appropriate.

# Introduction

We are writing to inform you about a research study in which you are invited to participate. The study has been approved by an Ethics Committee for Research with medicines and by the Spanish Agency for Medicines and Health Products, in accordance with current legislation, Royal Decree 1090/2015 of 4 December and European Regulation 536/2014 of 16 April, which regulates clinical trials with medicines.

Our intention is that you receive the correct and sufficient information so that you can decide whether or not to accept this study. To do this, read this fact sheet carefully and we will clarify any doubts that may arise. In addition, you can consult with the people you see fit.

# Voluntary participation

We invite you to participate in the study because you have had an acute ischemic stroke (a heart attack or lack of blood supply) of the anterior territory secondary to tandem lesion (a special type of lesion to the cerebral blood vessels) that requires thrombectomy treatment (intravenous drugs that "break" the thrombus) and need to place a cervical stent (a device that helps prevent this type of stroke from occurring in the future). The placement of the stent makes it necessary to use another group of treatments called antiplatelet agents that prevent the device from becoming obstructed but can increase side effects such as bleeding from different locations. This study aims to know which treatment is most appropriate.

You should know that your participation in this study is voluntary and that you may decide NOT to participate. If you choose to participate, you can change your decision and withdraw consent at any time, without altering your relationship with your doctor or harming your health care.

# Objective of the study

To assess the efficacy and safety of the use of an antiplatelet called tirofiban compared to aspirin (also antiplatelet) in patients with ischaemic stroke such as the one you have suffered, i.e. secondary to tandem lesion. To do this, we want to measure in what proportion of patients have stent seclusion in the acute phase of treatment at 24 hours as well as measure the presence of hemorrhages that cause symptoms according to international scales in use.

# Description of the study

The procedures that will be performed in the study are those usually used in the treatment of this pathology, that is, they are aimed at reducing in the shortest possible time the occlusion of the artery that has caused the stroke. For this you will undergo a catheterization, the administration of thrombectomy (rupture of the thrombus with drugs) and the placement of a carotid stent (in the neck), for which you will require an antiplatelet treatment, which is what we intend to study. In order to draw valid conclusions from this comparison, it is planned to include a total of 240

Patients who have suffered an acute ischemic stroke of the anterior territory secondary to tandem lesion, with indication of thrombectomy treatment and need for cervical stent placement. In order to compare medications, you will be randomly assigned (like when we flip a coin) to receive.

- an intravenous injection of the experimental drug (tirofiban)
- Control drug (injectable aspirin).

Both you and your doctor will know the treatment to which you have been assigned, only a study collaborator will evaluate all the data without knowing the treatment, to avoid the influence that this could have on the interpretation of the results.

# Activities of the study

A total study duration of three years is expected from the inclusion of the first patient. The study is structured in five face-to-face visits and a last one by telephone:

- Visit 1 – patients will be informed of the study, will be reviewed if they meet the eligibility criteria of the study and, if they do, patients will accept their participation with the signing of the informed consent
- Visit 2 – administration of a single dose of study medication
- Visit 3 (at 24-36h after the administration of the study medication) – performance of safety analysis, CT and Angio-CT of the skull and study of cerebral irrigation (ultrasound).
- Visit 4 – will coincide with the patient's hospital discharge
- Visit 5 – follow-up 30 days after treatment administration
- Visit 6 - telephone follow-up 90 days after treatment administration.

Summary of the schedule of visits and procedures:

| **Procedures** | **Visit 1 Inclusion** | **Visit 2 (Day 0) Admon medication** | **Visit 3**  **24-36 hours** | **Visit 4 Hospital discharge** | **Visit 5**  **Follow-up 30 days post-inclusion**  **(± 7d)** | **Visit 6 (90 days post-inclusion (± 7d) –**  **Telephone** |
| --- | --- | --- | --- | --- | --- | --- |
| Consent  informed | X |  |  |  |  |  |
| Review criteria of incl/excl.. | X |  |  |  |  |  |
| Randomization | X |  |  |  |  |  |
| History / DemograpsICH | X |  |  |  |  |  |
| Vital signs (TA, HR, FR,  weight) | X | X | X | X | x |  |
| Hemogram | X |  | X | X | X |  |
| Biochemistry | X |  | X |  |  |  |
| Coagulation | X |  | X | X | X |  |
| NIHSS Scale | X |  | X | X |  |  |
| Pregnancy test | X |  |  |  |  |  |
| Electrocardiogram ma | X |  |  |  |  |  |
| Modified Rankin scale (pre-stroke in  Visit 1) | X |  |  | X |  | X |
| CT and Angio- | X |  |  |  |  |  |
| CT scan of skull |  |  | X |  |  |  |
| Signs and symptoms of stroke | X |  |  |  |  |  |
| Biomarker sub-study |  | X |  |  |  |  |
| Admon Tirofiban/drug control |  | X |  |  |  |  |
| Doppler TSA |  |  | X |  | X |  |
| Concomitant medication | X | X | X | X | X | X |
| Adverse events |  | X | X | X | X | X |

*CT angiography will be performed 24 hours after the procedure in case of doubts in the Doppler, or existence of severe restenosis or intra-stent occlusion. TA, blood pressure; HR, heart rate; RF, respiratory rate; NIHSS: *National Institute of Health Stroke Scale*; TSA: supraaortic trunks.

The experimental drug is a treatment that serves to maintain the opening or patency of a stent or mesh in the internal carotid artery that has caused an acute stroke. This mesh will be placed during an urgent procedure to restore blood flow to your brain. The procedure of

Intravenous treatment is performed at the same time as endovascular treatment by expert and specialized personnel. Sedation or general anaesthesia is usually used for endovascular treatment.

Once a vein is punctured and channeled, we proceed to inject controlled by an infusion pump a very low amount of intravenous tirofiban for 24 hours or intravenous aspirin in about 10 minutes. As a general rule, after the procedure you will be admitted to the Stroke Unit for observation, blood pressure management and treatment if necessary.

Mechanical thrombectomy uses iodinated contrast (which is introduced through catheters) and X-rays, which are ionizing radiation. After the procedure, these tubes will be removed immediately or in the following days and the puncture point will be closed with a sealing system so that no blood (hematoma) collects.

If you have agreed to participate in the biomarker sub-study, a blood sample will be drawn.

# Risks and inconveniences arising from your participation in the study

Related to study medication

The study medication is authorized and marketed for use in Spain. The experimental drug (tirofiban) is indicated for the prevention of a specific type of early myocardial infarction (without ST-segment elevation) whose last episode of chest pain has occurred in the last 12 hours and which present changes in the electrocardiogram and / or increase in cardiac enzymes. The control drug (injectable aspirin) is indicated for rheumatic, neuralgic, post-traumatic, post-operative, postpartum, neoplastic pain, as well as in the prophylaxis and treatment of thromboembolic disease, and hyperthermia of any etiology.

There is no agreement on which drug is the most indicated in the case of needing the administration of an antiplatelet agent and thrombolytic treatment, the results of published studies are not sufficient to make a recommendation in this regard, so it is proposed to carry out this clinical study in which you are invited to participate.

As both medicines are authorised, there is accessible information in the package insert about the side effects of both Tirofiban and ASA injection, so you can check with your study doctor for this information.

Summary of the safety profile of Tirofiban

- The most frequently reported adverse reaction during therapy with tirofiban, when used concomitantly with heparin, aspirin and other oral antiplatelet agents, was bleeding (usually mild bleeding in the skin or mucous membranes or minor bleeding at the catheterization site).
- Gastrointestinal (digestive system), retroperitoneal (behind the peritoneum), intracranial, haemorrhoidal and postoperative haemorrhage, epidural haematoma in the vertebral region, haemopericardium and pulmonary (alveolar) haemorrhage have also been reported.
- The most serious adverse reaction was fatal bleeding.
- In pivotal studies, tirofiban administration was associated with thrombocytopenia (platelet count <90,000/mm3), which occurred in 1.5% of patients treated with tirofiban and heparin. The incidence of severe thrombocytopenia (platelet count <50,000/mm3) was 0.3%. Non-haemorrhagic adverse reactions of tirofiban administered concomitantly with heparin were nausea (1.7%), fever (1.5%) and headache (1.1%).

Summary of the safety profile of ASA injection

According to the technical sheet, the frequency cannot be estimated based on the available data, so the frequencies are presented as "frequency not known".

- Hemorrhagic syndromes (epistaxis, bleeding gums, purpura, etc.) with an increase in bleeding time. The risk of bleeding may persist for 4-8 days after discontinuation of aspirin, which may cause an increased risk of bleeding in case of surgery. Gastrointestinal and intracranial bleeding may also occur.
- Hypersensitivity reactions, anaphylactic reactions, asthma, angioedema.
- Headache seasickness sensation of loss of audition tinnitus which are usually indicative of an overdose.
- Intracranial hemorrhage.
- Abdominal pain
- Occult or overt gastrointestinal bleeding (hematemesis, melena, etc.) leading to iron deficiency anemia. The risk of bleeding is dose-dependent. Gastric ulcers and perforations.
- Elevation of liver enzymes, liver damage, mainly hepatocellular.
- Skin and subcutaneous tissue disorders
- Hives, skin reactions.
- Reye's syndrome, pain and local skin reactions at the injection site.

Related to other study procedures

The common risks to any angiography or catheterization are the formation of a clot (thrombus) in the vessel being studied or in the tube (catheter) that could leave that area without irrigation or move to a vessel of the brain (occurs in one every 300 cases) causing a lack of transient or permanent irrigation (thrombosis) or the accumulation of blood or hematoma at the site of the puncture that occurs in 5%.

The use of iodinated contrast could exceptionally trigger an allergic reaction that is usually mild and immediate (hives, itching, redness, etc.) or very unlikely serious (edema of the larynx, drop in blood pressure, etc.). Death could occur in one in every hundred thousand studies. If you are allergic to metals or iodine (contrast media), you should be warned before having this test. In cases of heart or kidney disease, these pathologies could be aggravated by the use of iodinated contrast.

The most common risks from sedation or anesthesia are nausea, vomiting, dizziness, headache, throat irritation, changes in blood pressure, and pain. These side effects are usually moderate, short, and treatable. In the long term, it can provoke anxiety, sleep disorders,

nightmares, flashbacks of the event, post-traumatic stress and fear of anesthesia. However, the percentage of cases in which it occurs is really low.

Specific risk of the treatment to be performed: Rupture of the artery to be treated and bleeding as a result, which may cause a cerebral hemorrhage, which may require urgent surgical intervention for treatment. Since late treatment is to be performed, it can theoretically increase the frequency of brain hematomas

X-rays are used, which are ionizing radiations. Its use is very safe and its advantages far outweigh its disadvantages. However, there are some risks, even if minimal, from radiation. Therefore, it must be warned before its realization of the existence of a pregnancy already known or of the suspicion that it may exist. There is a risk whose probability is remote: the appearance of tumors as a result of radiation.

In the case of the need to place a stent in patients with an acute stroke like you there is no demonstrated risk of increased frequency of cerebral or peripheral hemorrhages.

# Possible benefits

You may not get a direct benefit from your participation in this study. However, the evaluation of new therapeutic options to address the management of atherosclerotic extracranial lesion could in the future improve the prognosis in other patients who suffer from it.

# Contact in case of doubts

If you have any questions or need more information during your participation, please contact <<Study Physician, including name, service, how to reach you, contact telephone number and hours of operation on that phone>>.

In case of urgency or emergency you should contact your usual center.

If you need medical care by a team other than the one that offered you to participate in this study, you must report your participation in this trial and provide

All possible information relating to the study. For example, indicate the importance of carrying the card that has been provided at the time of inclusion.

# What treatment will I receive when the clinical trial ends?

When you finish your participation you will receive the best treatment available and that your doctor considers the most appropriate for your disease.

# Sure

The Study Promoter has an insurance policy that complies with current legislation (Royal Decree 1090/2015) and that will provide compensation and indemnity in case of impairment of your health or injuries that may occur in relation to your participation in the study, provided that they are not a consequence of the disease being studied or the evolution of your disease as a result of the ineffectiveness of the treatment.

For more information regarding this section, consult with the principal investigator of the study in your center, with the CEIm that has evaluated the study, or the information related to the study available in the Spanish Registry of Clinical Studies (<https://reec.aemps.es/>).

# Protection of personal data

Both the promoter and the center will ensure that the principles contemplated in the data protection regulations, both national and European, are complied with.

For more information on confidentiality and protection of personal data, please refer to Appendix 1.

What will my data be used for?

Your data is necessary for the sponsor to develop the medicine, obtain permission to introduce and maintain it on the market, monitor its safety and be covered by health insurance, i.e. throughout the drug development programme. Therefore, they will be used as planned in this study, as well as within the related research activities necessary for this drug development program in order to:

- understand how the study drug and similar drugs work in the body,
- better understand the disease studied and associated health problems,
- learn from previous studies to plan new studies or improve methods of scientific analysis,
- Publish research results in scientific journals or use them for educational purposes.

# Other relevant information

A description of this clinical trial will be available in [http://reec.aemps.es](http://reec.aemps.es/), as required by Spanish law.

Any new information regarding the drugs used in the study that may affect your willingness to participate in the study, which is discovered during your participation, will be communicated to you by the investigators as soon as possible.

You should know that you may be excluded from the study if the sponsor or the study investigators consider it appropriate, either for safety reasons (your disease does not respond adequately, any adverse event that occurs due to the medication under study, etc.) or because they consider that you are not complying with established procedures. In either case, you will receive an adequate explanation of the reason for your withdrawal from the study.

By signing the attached consent sheet, you agree to comply with the study procedures that have been exposed to you.

# Sub-study of biomarkers

All study participants are offered the possibility to participate in a sub-study of obtaining blood samples for the analysis of biomarkers in order to:

- To improve pathophysiological knowledge of the complications associated with endovascular treatment of tandem lesions (re-occlusion) through the identification of related plasma biomarkers.
- Assessment of any of the biomarkers identified as a therapeutic target of reocclusion.

If you are interested in participating in this sub-study you must record it at the end of the informed consent of the general ATILA study, an additional blood sample will be drawn at the first study visit and before the administration of the experimental treatment / control.

# Collection and use of biological samples

Objectives

Your participation in this clinical trial involves the collection and use of biological samples for research purposes, for which Law 14/2007 on biomedical research and Royal Decree 1716/2011 will be observed, regulations that guarantee respect for the rights that assist you. By signing this document, reviewed and favorably evaluated by the Drug Research Etichs Committee that has approved this clinical trial, you agree that your samples will be used for the purposes of this study. Blood samples will only be drawn to monitor the safety of the medication administered and, if you have agreed to participate in the biomarker study, for the biomarker sub-study.

# Sampling procedures, nuisances and possible risks

Some of the samples will be obtained during the usual follow-up of your disease or process; Others will be requested because they will be necessary to meet the objectives of this study. Below we explain what they are and the risks associated with the procedures used to obtain them:

- Blood samples: 4 blood samples will be obtained (6 if you agree to participate in the biomarker sub-study) and the amount extracted in each analysis will be 9 ml of blood. For most people, needle sticks for blood collection are not a problem. However, occasionally, they can cause bleeding, bruising, discomfort, infection and/or pain at the point of blood collection. You may also feel dizzy.

The possible risks arising from the procedure carried out to obtain these samples will be covered by the insurance of the clinical trial.

The samples will be associated with a code that can only be related to their identity by authorized personnel in the study. The data derived from the use of these samples will be treated in the same way as the rest of the data obtained during this test (see section on the protection of personal data).

The samples and associated data will be kept under appropriate security conditions and it is guaranteed that the subjects cannot be identified through means considered reasonable by persons other than those authorized.

# Expected benefits

No direct benefit is expected from your participation in the study. However, the knowledge gained from studies carried out from your samples and many others can help medical progress and, therefore, other people. It shall not receive any financial benefit from the donation of the samples and the transfer of the data provided, nor shall it have rights to any commercial benefits of the discoveries that may be made as a result of the research carried out.

# Place of analysis and storage of samples

During the development of the assay, the samples belonging to the sub-study of biomarkers will be analyzed in the Neurovascular Research Laboratory (responsible: Dr. Elena Zapata of the Radiodiagnosis Service of the Virgen del Rocío University Hospital in Seville). The samples shall be kept in storage until the end of the test and all its associated analyses. Once the test is finished, the excess samples will be destroyed.

During this process, the promoter of the test will be responsible for the samples.

If you require additional information you can contact our interventional neuroradiology staff on the phone: 955013500 or email: [sevillanri@gmail.com](mailto:sevillanri@gmail.com)

# Future use of samples

Once the test is finished, the remaining samples will be destroyed, unless you consent to them being stored and used in future

Research (see Yes/No option at the end of the sheet) The purpose of storing these samples is to be used in future research projects. The samples obtained for the sub-study of biomarkers, will be stored in the private collection C330011 in the name of Dr Joan Montaner, in the Registry of Biobanks of Andalusia, and with number C.0003347 in the Carlos III health institute. They will not be transferred to third parties, and will be used in projects favorably reported by a Research Ethics Committee, and related to studies of biomarkers related to stroke.

In case the use or transfer of their samples in a different investigation is raised, their consent would be requested. You may contact (collection manager or principal investigator) for information on the projects in which your samples have been used.

# Right of revocation of consent

If you change your mind regarding the donation of the biological samples and the transfer of the data provided, you have the right to request their destruction or anonymization, through your doctor/researcher/principal investigator of the collection/biobank. However, you should be aware that the data obtained in the analyses carried out up to that moment may be used for the purposes requested and may be kept in compliance with the corresponding legal obligations.

# Implications of the information obtained when analyzing the samples

If you choose to participate in the study, the analysis of your biological samples may yield information relevant to your health or that of your family. In accordance with current legislation, you have the right to be informed of the data obtained in the course of the study.

If you want to know the relevant data for your health that are obtained, inform yourself through your doctor about the implications that this information may have for you and your family. This information will be communicated to you if you wish; In the event that you prefer not to be informed, your decision will be respected.

# Appendix 1. PROTECTION OF PERSONAL DATA RELATED TO THE DOCUMENT PATIENT INFORMATION SHEET AND CI OF THE ATILA STUDY

**Study title**: Multicenter randomized clinical trial to assess efficacy and safety of tirofiban versus intravenous aspirin in patients with acute ischemic stroke secondary to tandem lesion, undergoing recanalization therapy by endovascular treatment (ATILA Study).

**Study code**: ATILA-ictus-2021

**EudraCT No**.: 2021-003874-30

**PROMOTER:** Andalusian Public Foundation for the Management of Health Research in Seville (FISEVI).

# Who is responsible?

FISEVI is the promoter of this study. It is based in Seville.

Both the Centre and the Promoter are responsible for their respective treatments, each of them corresponding to the obligations derived from their activity. The center is responsible for all the data that appear in the history and that can identify you and the promoter of those that are collected in this study in coded form (pseudonymised).

The role of the data controller is to ensure that your information is used correctly, in particular by applying appropriate technical and organisational measures to ensure that the data is processed in accordance with the applicable regulations.

The promoter and the centre shall comply with the data protection regulations:

- Regulation (EU) 2016/679 of the European Parliament and of the Council of 27 April 2016 (GDPR) on the protection of natural persons with regard to

regarding the processing of personal data and the free movement of such data

- Organic Law 3/2018 of December 5, Protection of Personal Data and guarantee of digital rights (LOPDPGDD) and any other development regulation.

# What about confidentiality?

At all times, the confidentiality of your data will be maintained. During your participation in the study you will be identified by a code and neither the investigator nor the hospital will transfer to the sponsor any information that can directly identify you.

The list that relates the identification code with the data that identifies you (name, surname, medical record number,...) are kept confidential in your health center.

Access to your identified personal information will be restricted to the study doctor / collaborators, health authorities (Spanish Agency for Medicines and Health Products, foreign health authorities), the Ethics Committee for Research with Medicines (CEIm) and personnel authorized by the sponsor (monitors and study managers), when they need it to verify the data, study procedures, and compliance with standards of good clinical practice; but always maintaining the confidentiality of them. Your identity may be revealed in exceptional cases, such as medical emergencies for your health or legal requirement. The processing, communication and transfer of personal data of all participants will comply with the provisions of the applicable regulations.

# How long will your data be kept?

All the information we request is necessary to participate in this study and it is mandatory to provide it in order to guarantee its correct development.

The Center, the researcher and the Sponsor are obliged to keep the data collected for the study according to the legal deadlines established in the regulations. The

sponsor and investigator for at least 25 years after the end of the study (according to clinical trial regulations) and the center for the time necessary to provide adequate care (according to regulations governing clinical history).

# What rights do I have?

With respect to your data, you have the following rights that you can exercise before the principal investigator and / or center:

- You can ask at any time what data is being stored (right of access), who uses it and for what purpose; You may request a copy of your personal data for your own use.
- You may request to receive a copy of the personal data provided by you in order to transmit it to other persons (portability).
- You can correct the personal data provided by you and limit the use of data that is incorrect (right of rectification and deletion).
- You can object to the use of your personal data or restrict it (right to object).

In relation to the rights over your personal data, we remind you that there are some limitations in order to guarantee the validity of the research and comply with the legal duties of the sponsor and the requirements of authorization of medicines. If you decide to stop participating in the trial or withdraw your consent to the processing of your data, the data collected up to that point cannot be deleted. You should know that if you decide to withdraw consent to the processing of your data, it could lead to your cessation of participation in the trial.

To protect your rights, we will use as little information as possible. We also inform you of your right to file a claim with the Data Protection Agency before any action of the Promoter or the Center that you consider violates your rights in terms of data protection.

# Who do I contact?

You can contact the data protection officer of your center, or contact the data protection officer of the promoter

Contact details of the Data Protection Officer (DPO) of the Centre or institution: [dpd.sspa@juntadeandalucia.es](mailto:dpd.sspa@juntadeandalucia.es)

Contact details of the DPO of the promoter: [dpd.sspa@juntadeandalucia.es](mailto:dpd.sspa@juntadeandalucia.es)

# How will the results be communicated?

The sponsor will publish the results of the study through the Spanish Register of Clinical Trials and the EU Clinical trials register <https://www.clinicaltrialsregister.eu/>.

The sponsor is obliged to publish the results, both positive and negative, of the authorised clinical trials, preferably, in scientific journals before they are disclosed to the non-health public, regardless of the obligations to publish the report of the results in the Spanish Register of Clinical Studies (REec) and the provisions in this regard in Regulation (EU) No 536/2014 of the European Parliament and of the Council, of 16 April 2014.

When studies and research papers on medicines are made public, aimed at the scientific community, the funds obtained by the author, by or for their realization, and the source of funding will be stated.

The anonymity of the subjects participating in the trial shall be maintained at all times.

# Safeguards for the protection of your personal data

Appropriate safeguards shall be taken to protect encrypted data during and after the test, including:

- Access to encrypted data shall be limited to persons subject to confidentiality obligations (including the obligation not to attempt to re-identify patients or decode clinical data).
- Encrypted data shall be protected by security measures to prevent alteration, loss and unauthorised access and additional measures may be applied to prevent identification.
- A data protection impact assessment shall be applied to identify and mitigate potential privacy risks, if any, associated with each scientific investigation.
- The encrypted data will not be shared for direct marketing purposes or for purposes other than legal obligations or that are not considered scientific research in accordance with current data protection legislation. In particular, they will not be used to make decisions about future services that may be offered to you, such as insurance.

**INFORMED CONSENT**

| STUDY TITLE | Multicentre randomised clinical trial to assess efficacy and safety of tirofiban versus intravenous aspirin in patients with acute ischaemic stroke secondary to tandem lesion undergoing recanalisation therapy by endovascular therapy (ATILA Study). |
| --- | --- |
| STUDY CODE | ATTILA-stroke-2021 |
| EudraCT NUMBER | 2021-003874-30 |
| PROMOTER | Andalusian Public Foundation for the Management of Health Research in Seville (FISEVI). |
| PRINCIPAL INVESTIGATOR |  |
| CENTER |  |

I last name of the participant)

(name and

- I have read the information sheet and appendix 1 that has been given to me about the study.
- I have been able to ask questions about the study.
- I have received enough information about the study.
- I spoken with (name and surname of the researcher)
- I understand that my participation is voluntary.
- I understand that I can withdraw from the study:
  - Whenever.
  - Without having to give explanations.
  - Without this affecting my medical care.

I will receive a signed and dated copy of this information and informed consent sheet

I freely agree to participate in the study, I confirm that I have read the

**Appendix 1** and I agree with its contents.

| Participant's signature | Signature of the  researcher |
| --- | --- |
| Date: / / | Date: / / |



(Name, signature and date to be filled in by the participant)

# When IQ is obtained in people with modified ability to give their IQ

| Signature of the representative legal, family or Related person in fact |
| --- |
| Date: / / |

(Name, signature and date to be filled in by the legal representative, family member or related person)

I want to be **told about information derived from the research** that may be relevant to my health:

- YES
- NO

| Participant's signature |
| --- |
| Date: / / |

(Name, signature and date to be filled in by the participant)

| Signature of the representative legal, family or Related person in fact |
| --- |
| Date: / / |

(Name, signature and date to be filled in by the legal representative, family member or related person)

**Regarding the biomarker sub-study: I agree to participate in the biomarker sub-study and the extraction of the specific blood samples explained in the information sheet:**

- YES
- NO

I want the study doctor to communicate to me information derived from the research that may be relevant and applicable to my health or that of my family members:

- YES Contact telephone or e-mail (participant or legal representative, family member or person Linked of Done)



- NO

I consent to the storage and use of biological samples and associated data for future research under the conditions explained in this information sheet.

- YES
- NO

I agree to be contacted in case I need more information or additional biological samples.

- YES Telephone o contact e-mail (participant or legal representative, family member or person Linked of Done)



- NO

| Participant's signature |
| --- |
| Date: / / |

(Name, signature and date to be filled in by the participant)

| Signature of the representative legal, family or Related person in fact |
| --- |
| Date: / / |

(Name, signature and date to be filled in by the legal representative, family member or related person).

# INFORMED CONSENT BEFORE WITNESSES

| STUDY TITLE | Multicentre randomised clinical trial to assess efficacy and safety of tirofiban versus intravenous aspirin in patients with acute ischaemic stroke secondary to tandem lesion undergoing recanalisation therapy by endovascular therapy (ATILA Study). |
| --- | --- |
| STUDY CODE | ATTILA-stroke-2021 |
| EudraCT NUMBER | 2021-003874-30 |
| PROMOTER | Andalusian Public Foundation for the Management of Health Research in Seville (FISEVI). |
| PRINCIPAL INVESTIGATOR |  |
| CENTER |  |

I (name and surname of witness), as a witness, I affirm that in my presence D/Dª has been informed (name and surname of the participant) and the information sheet that has been given to him about the study has been read, so that:

- You have been able to ask questions about the study.
- You have received sufficient information about the study.
- Has spoken with

(name and surname of the researcher)

- You understand that your participation is voluntary.
- You understand that you can withdraw from the study:
  - Whenever.
  - Without having to give explanations.
  - Without this affecting your medical care.

The patient will receive a signed and dated copy of this information and informed consent sheet

The participant freely agrees to participate in the study and confirms that he has read **Appendix 1** and agrees with its contents.

| Signature of the witness | Signature of the  researcher |
| --- | --- |
| Date: / / | Date: / / |



(Name, signature and date to be filled in by the witness)

The **Participant wishes to be informed of the information derived from the research** What may be relevant to your health:

- YES
- NO

| Signature of the witness |
| --- |
| Date: / / |

(Name, signature and date to be filled in by the witness)

**Regarding the biomarker sub-study: I agree to participate in the biomarker sub-study and the extraction of the specific blood samples explained in the information sheet:**

- YES
- NO

I want the study doctor to communicate to me information derived from the research that may be relevant and applicable to my health or that of my family members:

- YES Telephone or email of contact (witness)



- NO

I consent to the storage and use of biological samples and associated data for future research under the conditions explained in this information sheet.

- YES
- NO

I agree to be contacted in case I need more information or additional biological samples.

- YES Telephone or email of contact (witness)



- NO

| Signature of the witness |
| --- |
| Date: / / |

(Name, signature and date to be filled in by the witness)

The study participant has indicated that he or she cannot read/write.

A study staff member has read the Patient Information Sheet document, reviewed and discussed it with the participant, and been given the opportunity to ask questions or consult with others.

The witness must be an impartial person, outside the study.

**ANNEX VIII. Description of the *National Institutes of Health Stroke Scale* (NIHSS)**


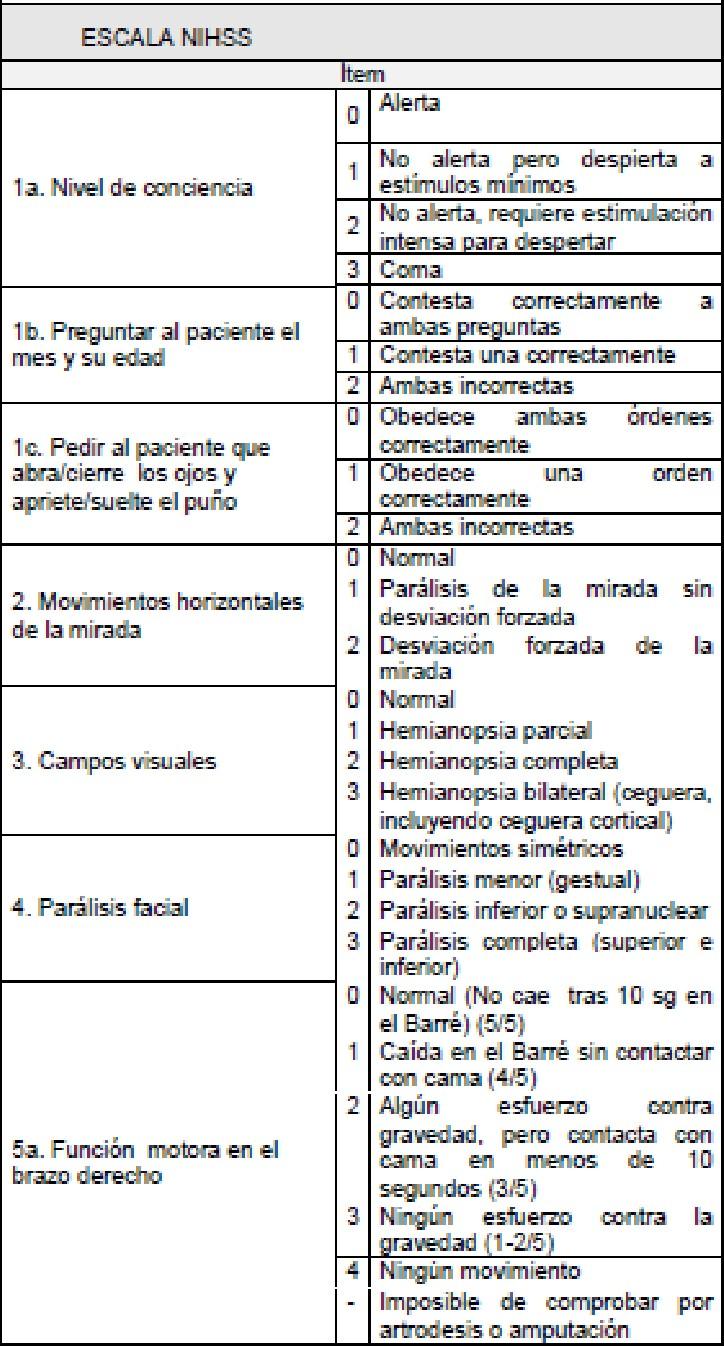


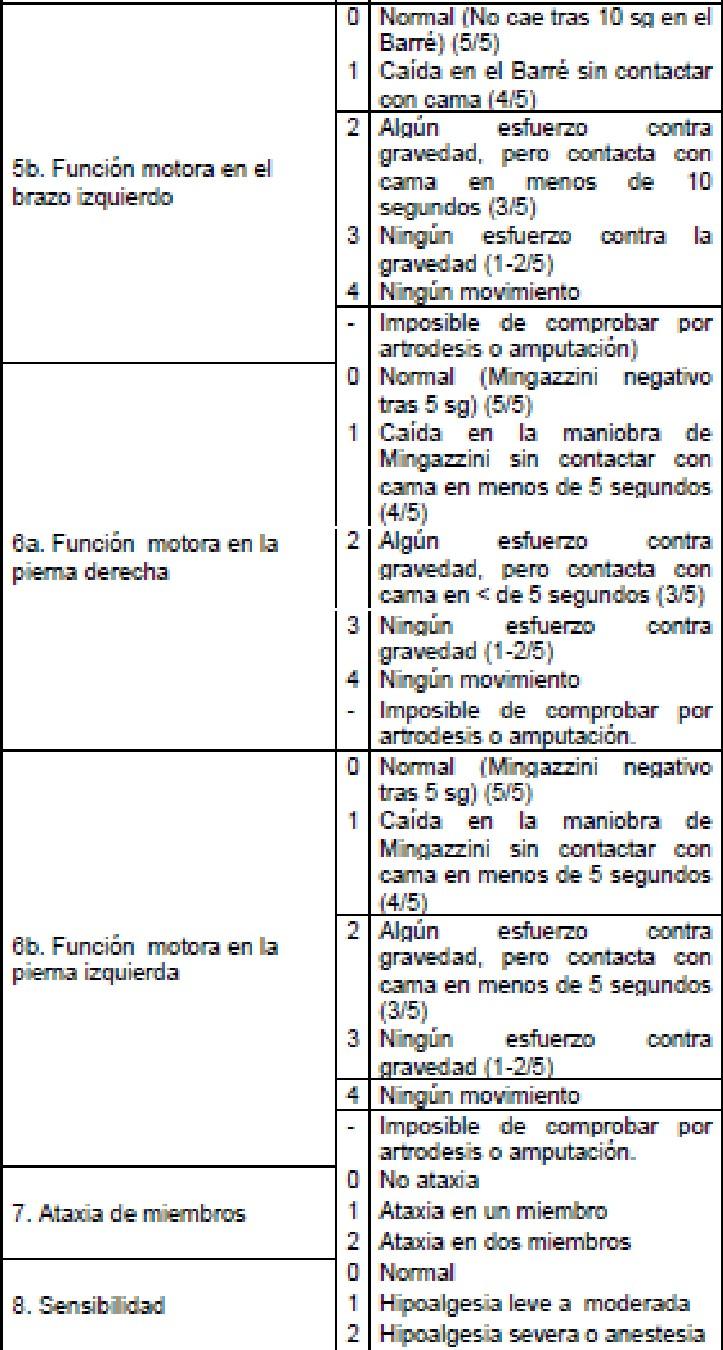


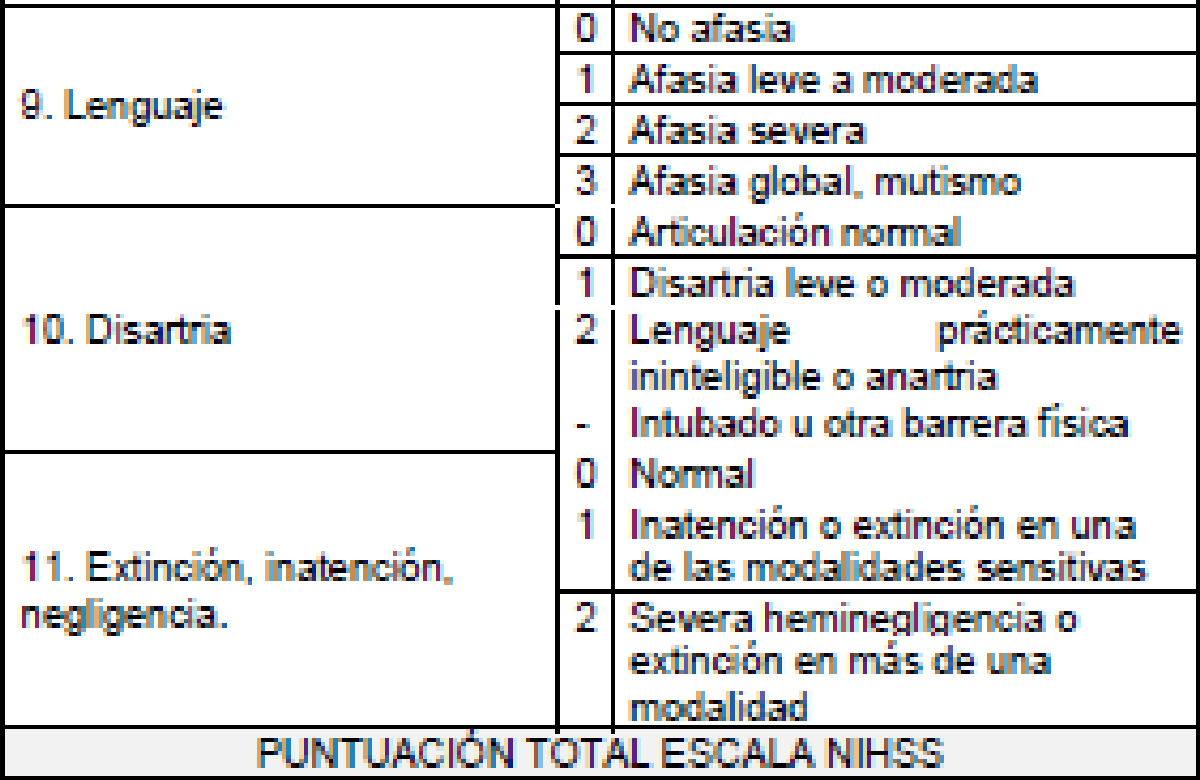


# ANNEX IX. Technical description of peripheral venous catheter placement

**Definitions Venoclysis**

It is the administration of diluted drugs intravenously through a continuous or intermittent drip system.

**Intermittent infusion**

The medication that is administered will be diluted in a small amount of saline or dextrose and will be transfused between 30 minutes or more depending on the medication.

**Infusion continues**

The drug is administered in a time greater than 12 hours, the volume of dilution will depend on the type of medication from 100 ml or more.

**Intravenous bolus**

A relatively high dose of a medication given intravenously over a short period of time, usually between 1 and 30 minutes.


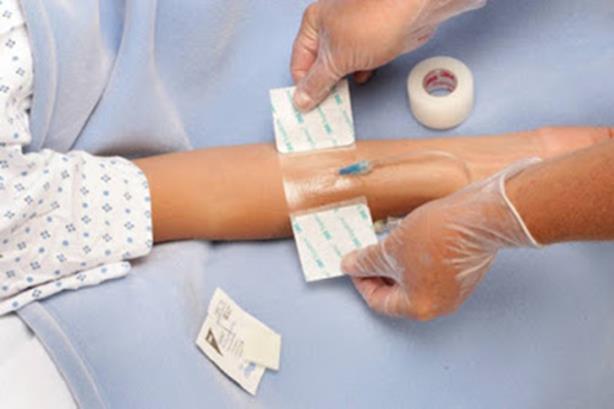


Track fixation

**Directions**

Peripheral venous catheter placement is indicated in the following situations:

- Administration of intravenous pharmacological treatment.
- Intravenous hydration in patients with oral intolerance.
- Resuscitation with intravenous solutions in patients in shock.
- Transfusion of blood products.

**Peripheral venous catheter placement procedure Materials**

- Sterile fanny pack or tray
- Barren field
- Cotton swabs
- 5ml syringes.
- Saline
- Peripheral venous catheter of various calibers (24, 22, 20, 18, 16)
- Three-way wrench with extension
- Tegaderm 6 x 7 or 10 x 12 cm
- Sterile gloves.
- Ligature
- Medical alcohol at 70°, iodized alcohol can also be used.


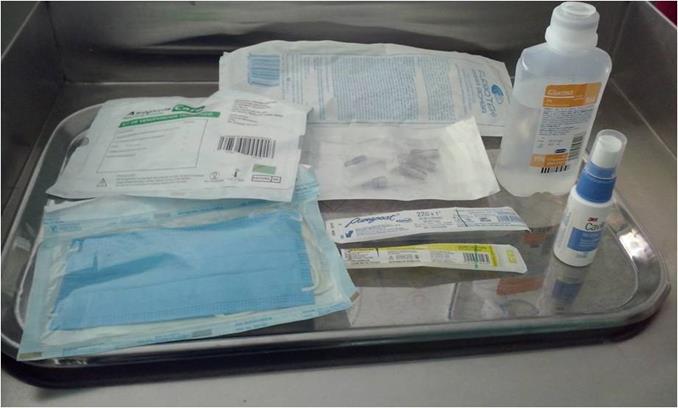


**Procedure**

- Clinical hand washing
- Putting on sterile gloves
- Choosing the vein
- Clean the puncture site with an alcohol-soaked swab
- Ligate the chosen arm in the area closest to the patient
- With the dominant hand insert the needle into the chosen vein at an angle of 30 to 40 degrees
- Once channeled, (which is evidenced by the exit of blood through the metal needle) progress a few millimeters, to later enter only the abbocath without the needle
- Release the ligature
- Connect the three-way key with the extension which must be purged with saline
- Fix the abbocath with tegaderm or fabric tape neatly
- Enter date of placement of the line and name of nurse
- Administer indicated medical treatment
- Clinical hand washing
- Register the procedure.


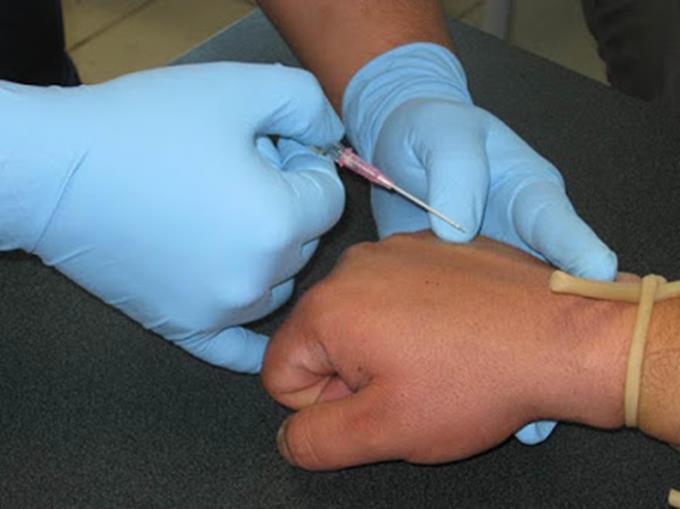


# ANNEX X. Description scale Modified treatment in cerebral ischemia (mTICI) score for intracranial recanalization post-thrombectomy


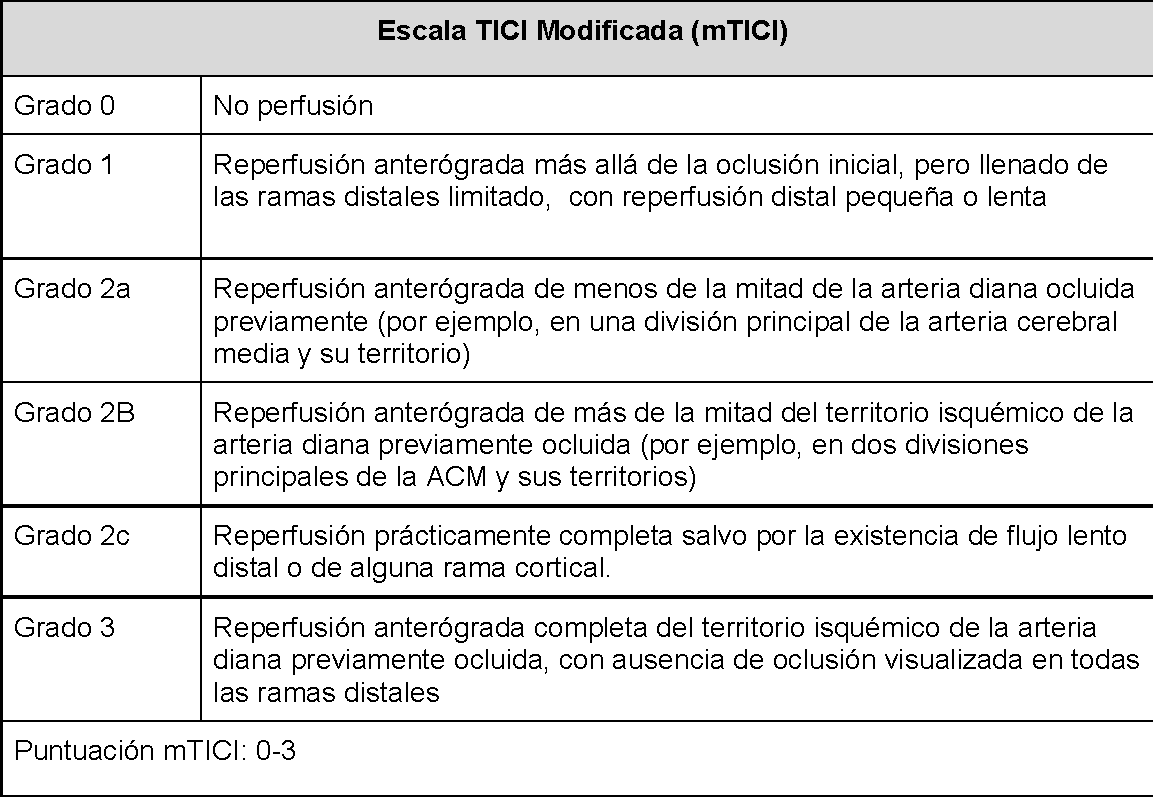


**ANNEX XI. Description of the ASPECTS scale (Alberta Stroke Protocol Programme Early CT Score)**

The ASPECTS scale allows, in a quantitative way, to give a numerical value to a qualitative assessment in which we assess incipient signs of ischemic brain involvement.

It consists of assessing the absence of differentiation of structures that we usually see in a TAC. Divided the cerebral hemisphere that has caused some damage into 10 segments:

- Caudate ( C)
- Lenticular (L)
- Insula (I)
- Internal capsule (IC)
- Cortical regions of the territory of the middle cerebral artery (M1-M6)

It is evaluated in two CT sections, one just at the level of the frontal horns of the lateral ventricle and the other cut in part convexity of the lateral ventricle. A score of 10 is that we have been able to differentiate all the structures. One of 0 is that no structures are identified and hypodense areas suggestive of established infarction are already observed.


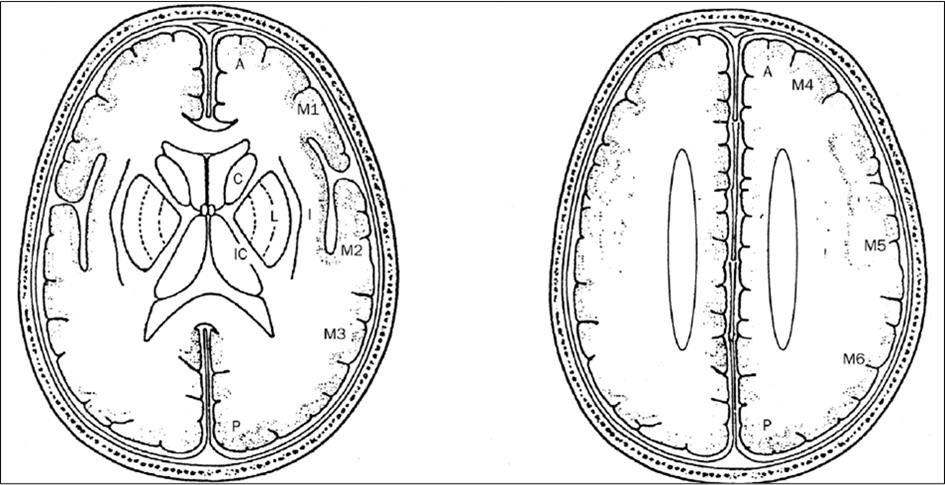


# ANNEX XII. Nomenclature of the hemorrhagic transformation of ischemic stroke according to ECASS criteria

| Nomenclature of hemorrhagic transformation of ischemic stroke | |
| --- | --- |
| H1 | Small petechiae along the margins of the infarction |
| H2 | Petechiae more confluent along the margins and in the nucleus of the infarction without space-occupying effect. |
| PH1 | Hematoma of small or intermediate size located in the center of the infarction not exceeding 30% of the volume of the infarction. Slight space-occupying effect. |
| PH2 | Large hematoma that exceeds 30% of the volume of the infarction with significant space occupying effect. |
| r | The r is added when it occurs in a remote location of the infarction area |

**ANNEX XIII. Sub-study of Blood Biomarkers**

Biomarkers Candidates:

There are no published data on biomarkers associated with intra-stent reocclusion in internal carotid artery lesions. The selected candidate biomarkers come from a pilot study conducted in coronary pathology. Those selected are: circulating superoxide dismutase 3 (SOD3), nitric oxide (NO), endothelial nitric oxide synthetase (eNOS). Both markers shall be determined by conventional ELISA techniques, according to the manufacturer's instructions. All determinations will be made blind to clinical data.

Discovery Studies:

An exploration of >500 proteins will be performed using OLINK technology.

Circulating levels of these proteins will be analyzed in the plasma of 30 patients (15 patients with reocclusion and 15 patients without reocclusion) using a small volume of 100 μl. A replication of the two most significant biomarkers will be carried out by ELISA. The independent predictors of reocclusion will be determined by logistic regression analysis, and the additional predictive value of the biomarkers described above and the two best biomarkers discovered through the use of the aptamer array, on the clinical variables will be evaluated by the integrated index of improvement of discrimination.

- The objectives for conducting this sub-study are secondary and are described below:
  - To improve pathophysiological knowledge of the complications associated with endovascular treatment of tandem lesions (re-occlusion) through the identification of related plasma biomarkers.
  - Assessment of any of the biomarkers identified as a therapeutic target of reocclusion.
- Blood draw.

Peripheral blood samples will be drawn by peripheral blood venipuncture in patients who agree to participate in the voluntary biomarker substudy. The following will be removed:

- - 1 tube of serum with separator gel of 9 ml. After extraction, the blood will be allowed to clot for 15-30 minutes at room temperature (18-22°C) and centrifuged at 2,000 x g, 15 min 4ºC. The serum shall be divided into 7 aliquots of not less than 300μl per aliquot and stored at -80°C until further analysis.
  - 1 EDTA plasma tube. It will be turned 2-3 times and left cold (4ºC) until centrifuged before 3h. It will be centrifuged at 2,000 x g,

15min, 4ºC. The plasma will be taken and deposited in 7 aliquots of not less than 300μl. An aliquot with the white series remaining at the plasma-cell interface of the tube will be collected. They will be stored at -80ºC until further analysis.

# ANNEX XIV. World Health Organization Table for Toxicity Gradation

|  | ***Nota: 0*** | ***Nota: 1*** | ***Nota: 2*** | ***Nota: 3*** | ***Nota: 4*** |
| --- | --- | --- | --- | --- | --- |
| ***Hematologic (Adults)*** | | | | | |
| Hemoglobin | >= 6.8  mmol/l  >110 g/l | 5.6 - 6.7 mmol/l 95 - 109 g/l | 4.95 - 5.8  mmol/l 80 - 94 g/l | 4.0 - 4.9 mmol/l 65 - 79 g/l | < 4.0 mmol/l  <65 g/l |
| Leukocytes (109/l) | >= 4.0 | 3.0 - 3.9 | 2.0 - 1.9 | 1.0 - 1.9 | < 1.0 |
| Granulocytes (109/l) | >= 2.0 | 1.5 - 1.9 | 1.0 - 1.4 | 0.5 - 0.9 | < 0.5 |
| Platelets (109/l) | > 100 | 75 – 99 | 50 - 74 | 25 - 49 | < 25 |
| Hemorrhage | None | Mild, petechiae, no transfusion | Mild blood loss, 1-2 units transfusion per episode | Gross blood loss, 3- 4 units transfusion per episode | Debilitating massive blood loss, > 4 units transfusion per episode |
| ***Hepatic*** | | | | | |
| Bilirubin | =<1.25 x N(a) | 1.26 - 2.5 x N(a) | 2.6 - 5 x N(a) | 5.1 - 10 x N(a) | > 10 x N(a) |
| Transaminases (ASAT/ALAT) | =<1.25 x N(a) | 1.26 - 2.5 x N(a) | 2.6 - 5 x N(a) | 5.1 - 10 x N(a) | > 10 x N(a) |
| Alkalinephosphata is | =<1.25 x N(a) | 1.26 - 2.5 x N(a) | 2.6 - 5 x N(a) | 5.1 - 10 x N(a) | > 10 x N(a) |
| Clinical | Notinclud ed | Notincluded | Notincluded | Precoma | Hepatic coma |
| ***Gastrointestinal*** | | | | | |
| Oral (mucositis; stomatitis) | No change | Soreness/erythema | Erythema, ulcers, can eat solids | Ulcers, requires liquid diet only | Ulcers with hemorrhage & necrosis, alimentation not possible |

| Esophagus | None | Mild fibrosis, no pain, slight difficulty with solid food | Fibrosis, mild pain, semi-solid food only | Severe fibrosis, severe pain, liquid only, dilatation req'd | Necrosis, fistula, perforation |
| --- | --- | --- | --- | --- | --- |
| Anorexia/weightlo ss | None | Anorexia withoutweightloss | Weightloss<5% | Weightloss 5-10% | Weightloss>10  % |
| Nausea/vomiting | None | Nausea withoutvomiting | Transient vomiting; 1-5 emetic episodes/day | Vomiting requiring therapy; 6-10 emetic episodes/day | Intractablevomi ting |
| Diarrhea | None | Transient (< 2  days); 3-4 loose stools/day | Tolerable, but  >2 days; 5-7 loose stools/day | Intolerable, requiring therapy;  > 7 loose stools/day | Hemorrhhagic; Dehydration&el ectrolyteimbala nce |
| ***Renal, bladder*** | | | | | |
| Blood urea (urea-nitrogen) or creatinine | =<1.25 x N(a) | 1.26 - 2.5 x N(a) | 2.6 - 5 x N(a) | 5.1 - 10 x N(a) | > 10 x N(a) |
| Creatinineclearanc e | Normal range | 0.75-0.99L(a) | 0.5-0.74L(a) | 0.25-0.49L(a) | <0.25L(a) |
| Proteinuria | No change | 1 +, < 3 g/l | 2 - 3+, 3 - 10  g/l | 4+, > 10 g/l | Nephroticsyndr ome |
| Hematuria | No change | Microscopic | Gross | Gross + clots | Obstructiveuro pathy |
| ***Pulmonary*** | | | | | |
| Respiratorysympto ms | None | Mildortransientdys pnea | Exertionaldysp nea | Dyspnea at rest, req's intermittent oxygen | Complete bed rest, continuous oxygen or assisted ventilation req'd |
| X-ray | Normal | Linear streaking | Bilateral, opacification< 50%  lungvolume | Opacification 50 -  75% | Opacification> 75% |

| Function | Normal | 2550% decrease in DCD or VC | > 50%  decrease in DCD or VC | Notincluded | Notincluded |
| --- | --- | --- | --- | --- | --- |
| ***Allergic*** | None | Transientrash, edema | Urticaria, bronchospasm; Non-parenteral therapyneeded | Serum sickness, bronchospasm; parenteral therapy required | Anaphylaxis |
| ***Cutaneous*** | No change | Erythema, macules, papules, pigmentation, slight atrophy, nail changes | Drydesquamati on, vesiculation, pruritus | Moistdesquamatio n, ulceration | Exfoliative dermatitis, necrosis requiring surgical intervention |
| ***Hair*** | No loss | Minimalhairloss | Moderate, patchy alopecia | Complete alopecia, but reversible | Complete & non-reversible alopecia |
| ***Infection, specifysite*** | None | Minor infection, no active Rx | Moderate infection, req's active Rx | Majorinfection, hospitalization | Major, life threatening, infection with hypotension |
| ***Cardiac*** | | | | | |
| Rhythm | Normal | ST-T changes, sinus tachycardia > 110 at rest | Unifocal PVC, atrial arrhythmia | Multifocal PVC | Ventricular tachycardia, intractable CHF |
| Function | No change | Asymptomatic, but abnormal cardiac sign | Transient asymptomatic dysfunction; no therapy required | Symptomatic dysfunction responsive to therapy | Symptomatic dysfunction non-responsive to therapy |
| Left Ventricular Ejection Fraction (LVEF) | No drop in LEV, as compared with initial value | LVEF drops 1-5% | LVEF drops 6-  10% | LVEF drops 11-15% | LVEF drops> 15% |
| Ischemia | Notscored | Notscored | Notscored | Transient angina or ischemic changes on EKG | Myocardialinfar ction |

| Pericarditis | No change | Asymptomatic effusion and/or ECG suggestive of pericarditis | | Symptomatic, no taprequired | Effusion req'g taps or constrictive pericarditis not req'g surgery | Tamponadeorc onstrictive pericarditis req'gpericardec tomy |
| --- | --- | --- | --- | --- | --- | --- |
| ***Neurologic*** | | | | | | |
| State of consciousness, cortical | Alert | Transientletharg yoragitation | Somnolence < 50% of waking hours or moderate agitation | | Severe somnolence  > 50% of waking hours; agitation or confusion; disorientation, hallucination | Coma; seizures or toxicpsychosis |
| Mood | No change | Mild, only mentioned after questioning | Moderate, easily in tears | | Severe, (anxiety, depression) visibly depressed/anxious, pharmacotherapy indicated | Suicidal ideation, clinical treatment indicated |
| Cerebellar | None | Slightincoordinat ion | Intention tremor, dysmetria, slurred speech, nystagmus | | Ataxia locomotive | Cerebellar necrosis |
| Peripheral, sensory | Normal | Mild paresthesias and/or decreased tendon reflexes | Moderateparesth esias, absentDTR's | | Severe paresthesias and/or disabling objective sensory loss interfering with function | Intolerable paresthesias and/or function loss |
| Peripheral motor | Normal | Subjectiveweakn ess | Mild objective weakness without significant impairment of function | | Objective weakness with impairment of function | Paralysis |
| Constipation (b) | None | Mild | Moderate | | Abdominal distension | Constipation, distension and vomiting req'g surgery |


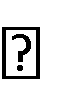

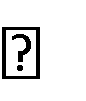

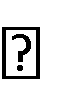

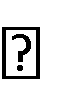


| Bladderfunction | Normal | Milddysfunction | Moderatedysfun ction | | Severebladderdysf unction | Dysfunction&di stensionreq'gsu rgery |
| --- | --- | --- | --- | --- | --- | --- |
| Pain (c) | None | Mild | Moderate | | Severe | Intractable |
| ***Othertoxicities*** | | | | | | |
|  | ***Grade : 0*** | ***Nota: 1*** | | ***Nota: 2*** | ***Nota: 3*** | ***Nota: 4*** |
| ***Feverwith drug*** | None | Fever< 38 C | | Fever 38 C -  40 C | Fever > 40 C, or with chills | Feverwithhypot ension |
| ***Fatigue*** | None | Mild; fatigue without decrease in daily activities | | Moderate, periodic interfering with performance; in bed < 50% of the day | Severe, clearly interfering with performance status; in bed > 50% of the day | State of exhaustion with very poor performance status; unable to take care of self |
| ***Flu-likesyndrome*** | None | Mild | | Moderate | Severe | Intractable |
| ***Headache*** | None | Mild | | Moderate | Severe | Intractable |
| ***Flushing*** | None | Mild | | Moderate | Severe | Intractable |
| ***Myalgia/arthralgi a*** | None | Mild | | Moderate | Severe | Disabling |
| ***Local reaction (injectionsite)*** | None | Pain | | Pain + inflammation | Ulceration | Surgeryindicate d |
| ***Vasculitis*** | None | Restrictedcutaneou s | | Generalizedcut aneous | Hemorhagic | Systemic |
| ***Other*** | None | Mild | | Moderate | Severe | Life- threatening |

N(a) = Upper limit of normal value of population under study. L(a) = lower normal value of the local institution b = This does not include constipation resultant from narcotics. c = Only treatment related pain is considered, not disease related pain (the usage of narcotics may be helpful in grading pain, depending upon the tolerance level of the patient).

# ANNEX XV. FORM OF NOTIFICATION OF SERIOUS ADVERSE EVENTS

| Subject Code: | | | | | REPORT TYPE: ☐Initial ☐ Tracking | | | | |
| --- | --- | --- | --- | --- | --- | --- | --- | --- | --- |
| **1. CENTER INFORMATION** | | | | | | | | | |
| School number: | | Principal Investigator: | | | | | | | |
| Person notifying the AAG: | | | | | | | Tel: | | |
| Fax: | | Mail: | | | | | | | |
| **2. SUBJECT INFORMATION** | | | | | | | | | |
| Sex | Age (At the beginning of the AA) | | Age group (fill in only if the age of the subject is unknown) | | | Date of birth  (dd-mmm-yy) | | Weight  (kg) | Height  (cm) |
| - Male - Female | ☐Years ☐Months ☐Days | | ☐Newborn  ☐Child  ☐Adult | ☐Baby  ☐Adolescent  ☐Old man | | _ _ – _ _ _ – _ _ _ _ | |  |  |
| **3. ADVERSE EVENT.** | | | | | | | | | |
| **Serious adverse event** (Specify the diagnosis or syndrome, if known. If signs and symptoms are unknown.)**:**  ............................................................................................................................................................................................................................................  ............................................................................................................................................................................................................................................ | | | | | | | | | |
| **DESCRIPTION OF THE ADVERSE EVENT** (Provide full information about the circumstances, sequence, diagnosis, and treatment of the AA) | | | | | | | | | |
|  | | | | | | | | | |
| Start date (dd-mmm-yy):_ _ – _ _ _ – _ _ _ _ End date: (dd-mmm-yy):_ _ – _ _ _ – _ _ _ _ | | | | | | | | | |
| Severity criteria:   - Death - Life-threatening - Requires or prolongs hospitalization - Permanent or significant disability - Birth defect or birth defect - Clinically relevant | | | | | Outcome (status at time of notification):   - Recovered   ☐In recovery   - Not recovered - Recovered with sequelae   ☐Mortal   - Unknown | | | | |
| In case of death, fill in the following information: | | | | | | | | | |
| Date of success: _ _ – _ _ _ – _ _ _ _ | | Cause: | | | | | | | |
| An autopsy was performed: ☐ Yes ☐No ☐ Unknown  Indicate the cause of death according to the autopsy: | | | | | | | | | |



| **4. INVESTIGATIONAL PRODUCT/MEDICINAL PRODUCT** (Indicate the treatment(s) assigned to the subject after randomisation)  If you need more space, use copies of this page and check this box  ☐ | | | | | | | | | | |
| --- | --- | --- | --- | --- | --- | --- | --- | --- | --- | --- |
| Medicament | Dosages and units | Frequency | | Road | Start date  (dd-mmm-yy) | End date  (dd-mmm-yy)  (if you continue to check the box) | | | Causal relationship | |
|  |  |  | |  |  | ☐ | | | ☐Related  ☐Unrelated | |
|  |  |  | |  |  | ☐ | | | ☐Related  ☐Unrelated | |
|  |  |  | |  |  | ☐ | | | ☐Related  ☐Unrelated | |
| Action taken with medication in response to AA | | | ☐Withdrawal of medication  ☐Temporary discontinuation of medication  ☐Unknown | | | | ☐Dose decrease  ☐Dose increase | | | ☐Unchanged  ☐Not applicable |
| Did the AA subside when stopping medication or reducing dose? | | | ☐Yes ☐No ☐Unknown ☐No Applies | | | | | | | |
| Did AA come back when medication was reintroduced ? | | | ☐Yes ☐No ☐Unknown ☐No Applies | | | | | | | |
| **5. CONCOMITANT MEDICATION** (Include those concomitant and basic treatments that you have taken in the two weeks prior to the start date of the AA. Do not include treatment given to treat AA or treatment given after the AA start date)  If you need more space, use copies of this page and check this box  ☐ | | | | | | | | | | |
| Medicament | Daily dose (units) | Frequency | | Road | Start date  (dd-mmm-yy) | End date (dd-mmm-yyyy) (if you continue  check the box) | | Causal relationship | | Indication |
|  |  |  | |  |  | ☐ | | ☐Related  ☐Unrelated | |  |
|  |  |  | |  |  | ☐ | | ☐Related  ☐Unrelated | |  |
|  |  |  | |  |  | ☐ | | ☐Related  ☐Unrelated | |  |
| Action taken with medication in response to AA | | | ☐Withdrawal of medication  ☐Temporary discontinuation of medication  ☐Unknown | | | | ☐Dose decrease  ☐Dose increase | | | ☐Unchanged  ☐Not applicable |
| Did the AA subside when stopping medication or reducing dose? | | | ☐Yes ☐No ☐Unknown ☐No Applies | | | | | | | |
| Did AA come back when medication was reintroduced ? | | | ☐Yes ☐No ☐Unknown ☐No Applies | | | | | | | |
| **6. ATLERNATIVE CAUSE** | | | | | | | | | | |
| Is there any possibility that AA is related to some other alternative cause to medication?  ☐Yes ☐No If yes, specify (for more information in the description section of the AAG if necessary): | | | | | | | | | | |

| **7. RELEVANT MEDICAL HISTORY**  If you need more space, use copies of this page and check this box  ☐ | | | | | | | | |
| --- | --- | --- | --- | --- | --- | --- | --- | --- |
| Pathological history | | Start date  (dd-mmm-yy) | | | | End date  (dd-mmm-yy) | | (if you continue to check the box) |
|  | |  | | | |  | | ☐ |
|  | |  | | | |  | | ☐ |
|  | |  | | | |  | | ☐ |
|  | |  | | | |  | | ☐ |
| **Complementary information of the data of the Medical History** | | | | | | | | |
|  | | | | | | | | |
| **8. LABORATORY DATA AND OTHER COMPLEMENTARY EXAMINATIONS:** (indicate only the results of tests relevant to document the reported AAG)  If you need more space, use copies of this page and check this box  ☐ | | | | | | | | |
| **TEST PERFORMED** | **TEST DATE**  (dd-mmm-yy) | | **RESUTL**  **(Units)** | **Rank**  **Reference** | | | **COMMENT** | |
|  |  | |  |  | | |  | |
|  |  | |  |  | | |  | |
|  |  | |  |  | | |  | |
|  |  | |  |  | | |  | |
| **Additional information on laboratory data and other examinations:** | | | | | | | | |
|  | | | | | | | | |
| **Signature of the notifying researcher** | | | | | **Date of notification** | | | |
|  | | | | |  | | | |
| **Signature of the Head of Pharmacovigilance** | | | | | **Date of receipt of notification** | | | |
|  | | | | |  | | | |

**SEND IMMEDIATELY BY FAX TO THE UICEC PHARMACOVIGILANCE NODE OF THE VIRGEN DEL ROCÍO UNIVERSITY HOSPITAL**

# (FAX: 955095338) / Email: [pv_atila@scren.es](mailto:pv_atila@scren.es)
