# Supplementary material for: Statistical analysis plan for the multicenter, open, randomized controlled clinical trial to assess the efficacy and safety of intravenous tirofiban vs aspirin in acute ischemic stroke due to tandem lesion, undergoing recanalization therapy by endovascular treatment (ATILA trial)
Source: Trials. 2024 Jan 9;25:35. doi: 10.1186/s13063-023-07817-9 (PMC10775524; doi:10.1186/s13063-023-07817-9)
Supplement: Supplementary file 2 — Additional file 2. Study Protocol. [file 13063_2023_7817_MOESM2_ESM.pdf]

Check for updates

Study Protocol

EUROPEAN STROKE JOURNAL

European Stroke Journal
2023, Vol. 8(1) 380–386
© European Stroke Organisation 2022
Article reuse guidelines:
sagepub.com/journals-permissions
DOI: 10.1177/23969873221146383
journals.sagepub.com/home/eso
SAGE

Safety and efficacy of tirofiban in acute ischemic stroke due to tandem lesions undergoing mechanical thrombectomy: A multicenter randomized clinical trial (ATILA) protocol

Manuel Medina-Rodríguez[1,2], Francisco Moniche[1,2], Asier de Albóniga-Chindurza[2,3], Joaquin Ortega-Quintanilla[2,3], Leire Ainz-Gómez[1,2], Blanca Pardo-Galiana[1,2], Juan Antonio Cabezas-Rodríguez[1,2], Marta Aguilar-Pérez[2,3], Aynara Zamora[2], Fernando Delgado-Acosta[4], Elvira Jiménez-Gómez[4], Isabel Bravo Rey[4], Rafael Oteros Fernández[4], María del Mar Freijo Guerrero[5], Eva González Díaz[6], Irene Escudero-Martínez[7], Lluis Morales Caba[8], Isabel Vielba-Gomez[8], Sonia Mosteiro[9], María del Mar Castellanos Rodrigo[10], Laura Amaya Pascasio[11], Carlos Hidalgo[12], Luis Fernandez Prudencio[13], Jose María Ramirez Moreno[14], Jose Díaz Pérez[15], Gema Sanz-Fernandez[16], Pablo Baena-Palomino[1,2], Miguel Ángel Gamero-García[2,17], Silvia Jiménez Jorge[18], Clara Rosso Fernández[18], Joan Montaner[2,17], Alejandro González García[2,3] and Elena Zapata-Arriaza[2,3]

Abstract

Background: In-stent thrombosis after mechanical thrombectomy (MT) worsen outcomes in acute ischemic stroke (AIS) due to tandem lesions (TL). Although an optimal antiplatelet therapy is needed, the best approach to avoid in-stent thrombosis is yet to be elucidated.

[1]Stroke Unit, Neurology Department, Virgen del Rocío University Hospital, Sevilla, Spain
[2]Neurovascular Research Program, Seville Biomedical Research Institute, Seville, Spain
[3]Interventional Neuroradiology Department, Virgen del Rocío University Hospital, Sevilla, Spain
[4]Interventional Neuroradiology Department, Reina Sofía University Hospital, Córdoba, Spain
[5]Neurology Department, Cruces University Hospital, Vizcaya, Spain
[6]Interventional Neuroradiology Department, Cruces University Hospital, Vizcaya, Spain
[7]Neurology Department, La Fe University and Polytechnic Hospital, Valencia, Spain
[8]Interventional Neuroradiology Department, La Fe University and Polytechnic Hospital, Valencia, Spain
[9]Interventional Neuroradiology Department, A Coruña University Hospital Complex, A Coruña, Spain
[10]Neurology Department, A Coruña University Hospital Complex, A Coruña, Spain
[11]Neurology Department, Torrecardenas University Hospital, Almería, Spain
[12]Interventional Neuroradiology Department, Torrecardenas University Hospital, Almería, Spain
[13]Interventional Neuroradiology Department, Badajoz University Hospital, Badajoz, Spain
[14]Neurology Department, Badajoz University Hospital, Badajoz, Spain
[15]Interventional Neuroradiology Department, Virgen de la Arrixaca University Clinical Hospital, Murcia, Spain
[16]Neurology Department, Juan Ramon Jimenez University Hospital, Huelva, Spain
[17]Neurology Department, Virgen Macarena University Hospital, Seville, Spain
[18]Clinical Research and Clinical Trials Support Unit, Virgen del Rocio University Hospital, Sevilla, Spain

Corresponding author:
Elena Zapata-Arriaza, Interventional Neuroradiology Department, Virgen del Rocío University Hospital and Neurovascular Lab-Seville biomedical Research Institute, Av Manuel Siurot S/N, Seville 41013, Spain.
Email: elena.zpt@gmail.com

Hypothesis: Low-dose intravenous tirofiban is superior to intravenous aspirin in avoiding in-stent thrombosis in patients undergoing MT plus carotid stenting in the setting of AIS due to TL.

Methods: The ATILA-trial is a multicenter, prospective, phase IV, randomized, controlled (aspirin group as control), assessor-blinded clinical trial. Patients fulfilling inclusion criteria (AIS due to TL, ASPECTS ⩾ 6, pre-stroke modified Rankin Scale ⩽2 and onset <24 h) will be randomized (1:1) at MT onset to experimental (intravenous tirofiban) or control group (intravenous aspirin). Intravenous aspirin will be administered at a 500 mg single dose and tirofiban at a 500 µg bolus followed by a 200 µg/h infusion during first 22 h. All patients will be followed up to 3 months. Sample size estimated is 240 patients.

Outcomes: The primary efficacy outcome is the proportion of patients with carotid in-stent thrombosis within the first 24 h after MT. The primary safety outcome is the rate of symptomatic intracranial hemorrhage. Secondary outcomes include functional independence defined as modified Rankin Scale 0–2, proportion of patients undergoing rescue therapy due to in-stent aggregation during MT and carotid reocclusion at 30 days.

Discussion: ATILA-trial will be the first clinical trial regarding the best antiplatelet therapy to avoid in-stent thrombosis after MT in patients with TL.

Trial registration: NCT0522596.

Keywords

Tirofiban, ischemic stroke, tandem lesions, carotid stenting

Date received: 22 July 2022; accepted: 22 November 2022

Introduction and rationale

Acute ischemic stroke (AIS) due to tandem lesions (TL) is defined as severe stenosis or occlusion of cervical carotid artery associated with an ipsilateral intracranial occlusion. They represent approximately the 20% of AIS involving anterior circulation undergoing mechanical thrombectomy (MT).[1] Although TL had been associated with poor prognosis, recent studies showed similar rates of functional outcomes and symptomatic intracranial hemorrhage (sICH) compared to isolated intracranial occlusions.[2–4] Intravenous thrombolysis (IVT) followed by MT is considered the standard of care in TL,[5,6] but there is no consensus regarding the best therapy of the extracranial lesion. The main dilemma appears when the extracranial lesion requires carotid stenting and concomitant antiplatelet therapy (AT) to avoid in-stent thrombosis. AT may increase the rate of sICH and should be avoided within the first 90 min after IVT.[5] Intravenous (IV) aspirin is the most recommended drug. Although tirofiban might be an option when aspirin is not available or ineffective (level of evidence IIb), the effect of tirofiban has not been well studied in the setting of AIS.

Study purpose

To test the hypothesis that low-dose protocol of IV-tirofiban is superior to IV-aspirin in avoiding in-stent thrombosis at 24 h in patients undergoing MT plus carotid stenting in the setting of AIS due to TL.

Methods and design

ATILA is a national-multicenter, prospective, phase IV, randomized, controlled (aspirin group as control), assessor-blinded clinical trial of IV-tirofiban versus IV-aspirin in AIS due to TL undergoing MT. The study flow chart is shown in Figure 1. The trial is a national study conducted in 10 stroke centers in Spain. The protocol is registered in http://clinicaltrials.gov (NCT0522596) and approved by the Spanish Agency of Medicines and Medical Devices (Eudra-CT: 2021-003874.30).

Patient population

Inclusion criteria

1. Patients with AIS due to TL undergoing MT in whom carotid stent placement is planned during the procedure.
2. The locations of intracranial occlusion that may be included are: terminal internal carotid artery (“carotid T”), segments M1 and M2 of the middle cerebral artery, segment A1 of the anterior cerebral artery, and posterior cerebral artery with fetal origin.
3. Carotid stenosis degree must be ⩾70%, carotid near-to-occlusion or complete carotid occlusion.
4. The etiology of internal carotid lesion is presumed to be atherosclerotic.
5. ASPECTS ⩾ 6.
6. Age ⩾18 years.
7. Written informed consent. Informed consent by phone call to relatives is allowed due to the emergency situation.

Exclusion criteria

1. Age <18 years.
2. ASPECTS < 6.
3. Radiological findings of bilateral stroke or stroke involving both anterior and posterior circulation.

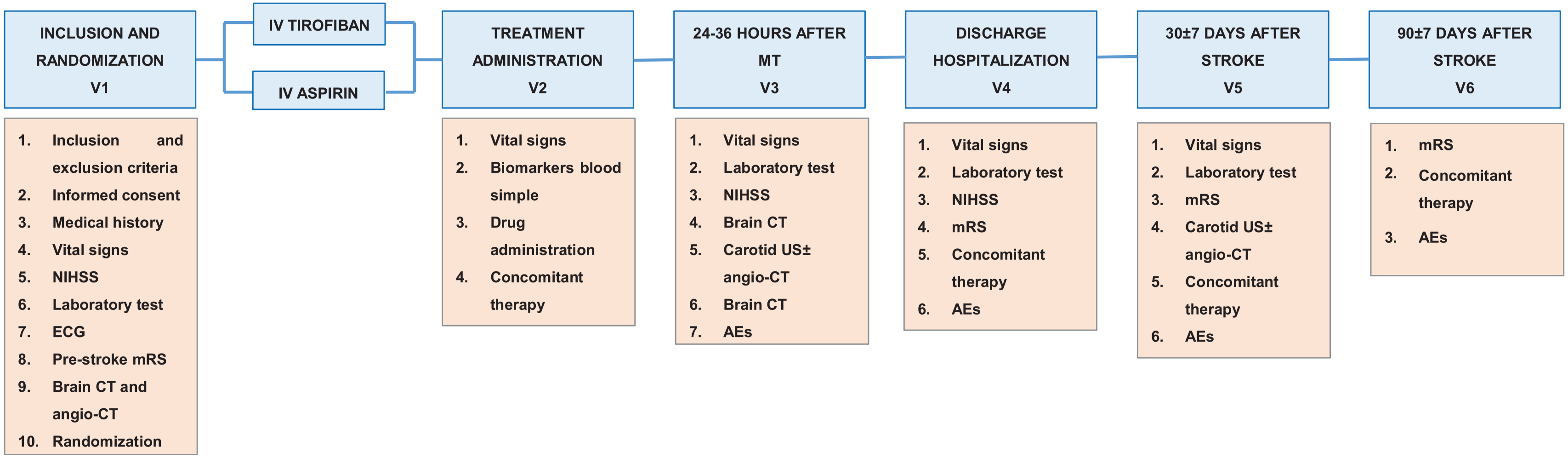

Figure 1. ATILA trial flow chart. In every patient a carotid ultrasound will be performed at 24 h. In case of moderate-to-severe stenosis or occlusion of the stent, an angio-CT will be performed to confirmed these findings.
NIHSS: National Institute of Health Stroke Scale; IV: intravenous; mRS: modified Rankin Scale; MT: mechanical thrombectomy; US: ultrasound; AE: adverse event.

4. Radiological findings suggesting internal carotid dissection or carotid web.
5. Carotid stenosis/occlusion due to carotid stent occlusion or previous endarterectomy.
6. Patients eligible for carotid puncture.
7. Patients under dual antiplatelet therapy at time of randomization.
8. Modified Rankin Scale >2.
9. History of allergy to iodinated contrast media.
10. Pregnancy.
11. Patients with atherosclerotic intracranial occlusion or arterial dissection.
12. History of allergy to aspirin or tirofiban.
13. History of platelet count <100.000 mm^3 or history of induced thrombocytopenia by GP IIb/IIIa inhibitors or iv aspirin.
14. Concomitant therapy with direct oral anticoagulants 48 h prior to randomization OR with vitamin-K-antagonist with INR > 1.7.
15. History of peptic ulcer in the last 3 months prior to randomization.
16. Any medical condition who may lead to high bleeding risk in the opinion of the investigator.

Randomization and blinding

After informed consent by the patient or relatives is obtained, patients will be randomized to either control group (IV-aspirin) or experimental group (IV-tirofiban) (experimental or control in ratio 1:1). Patients can be randomized at any time within baseline neuroimage and before groin-puncture. A blinded clinician, certified in carotid ultrasound and in assessment of the modified Rankin Scale (mRS), will undertake assessment of the primary outcomes by clinical visit.

Treatment

Mechanical thrombectomy. During the endovascular recanalization the first step is treating the intracranial occlusion and then carotid angioplasty, unless the cervical carotid stenosis or occlusion prevents the passage the devices for intracranial reperfusion, forcing cervical angioplasty prior to intracranial navigation. In both cases, a carotid stent is placed after intracranial reperfusion has occurred.

Study medication: Antiplatelet therapy. Every patient included will receive best medical treatment based on current CPG in AIS.[5] AT will be administered after groin-puncture and must be initiated before stenting. In the experimental group, 500 µg bolus of IV-tirofiban administered during 5 min, followed by a continuous infusion at a dose of 200 µg/h for 20 +/- 2 h will be used. In the control group, a single-dose 500 mg IV-aspirin (dose equivalent to 300 mg oral-aspirin) will be infused. Anticoagulants drugs, such as heparin, are not planned to be used during MT.

At day 1, a non-contrast computed tomography (CT) will be performed at 20 ± 2 h after MT. Once intracranial parenchymal hemorrhage (PH1 or PH2) is ruled out, patients enrolled in experimental group, as they did not receive the loading dose during MT procedure, will receive 300 mg oral aspirin (as loading dose) plus 300 mg clopidogrel and tirofiban infusion will be continued for four extra hours. They will receive both loading dose to assure the antiplatelet effect once the tirofiban infusion is stopped. Control group, as the 500 mg IV-aspirin is considered the loading dose, will receive 100 mg oral-aspirin plus 300 mg clopidogrel. The objective is to assure that patients are under dual antiplatelet therapy 20 ± 2 h after MT. From day 2, both groups will take 100 mg aspirin and 75 mg clopidogrel daily for 1 month and then clopidogrel will be discontinued (Figure 2).

Clinical and radiological assessments

Neurological severity will be measured using the National Institutes of Health Stroke Scale (NIHSS) at baseline and at 24 h after stroke onset. The mRS will be assessed at baseline, discharge hospitalization and at 3 months.

An initial CT and CT-angiography with intracranial occlusion involving the terminal internal carotid artery, the M1 or M2 segments of middle cerebral artery, A1 and the P1 (if the posterior cerebral artery has fetal origin) is required for study eligibility. In addition, a follow-up brain CT will be performed at 20 ± 2 h to identify intracranial hemorrhage (ICH). The primary outcome will be assessed by carotid ultrasound at 24–36 h after MT to identify in-stent thrombosis. In case of in-stent stenosis ⩾70% or occlusion identified on ultrasound doppler, it will be necessary to confirm with CT-angiography. In-stent stenosis ⩾70% by carotid ultrasound is defined as a peak systolic velocity (PSV) ⩾300 cm/s in the internal carotid artery. We defined in-stent occlusion as biphasic resistive and low velocity pattern in the site of occlusion and the absence of in-stent flow both in doppler and B-mode. ICH will be classified following the ECASS criteria. Symptomatic ICH (sICH) is defined as parenchymal hematoma type 2 within 36 h of treatment and neurological deterioration in the NIHSS scale ⩾4-points.[7]

Primary outcomes

The primary efficacy outcome is the proportion of patients with carotid in-stent thrombosis within the first 24 h after MT. We defined in-stent thrombosis as: (1) in-stent stenosis ⩾70% or (2) in-stent occlusion. The primary safety outcome is the rate of sICH.

Secondary outcomes

Secondary outcomes are: (1) proportion of patients undergoing rescue therapy due to in-stent platelet aggregation

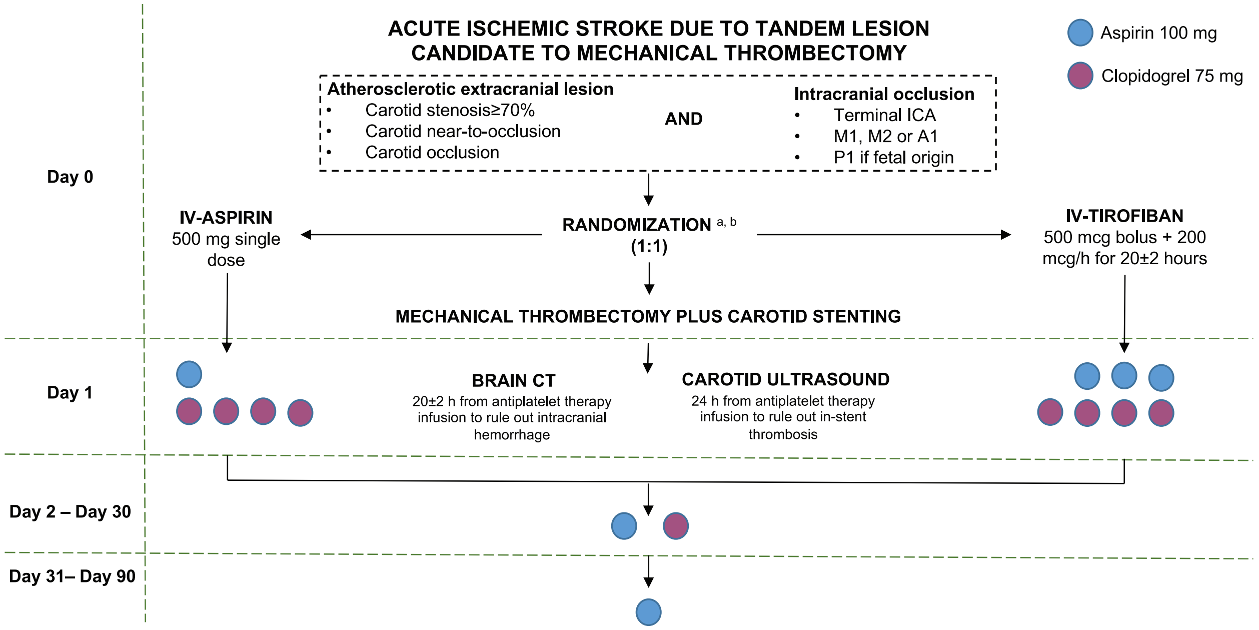

Figure 2. Timeline of ATILA-trial.
[a]Patients can be randomized at any time within baseline neuroimage and before groin-puncture.
[b]Antiplatelet therapy infusion must be initiated as soon as possible since groin-puncture and before carotid stent placement.

during MT; (2) rate of good functional outcomes at 90 days, defined as mRS ≤ 2; and (3) severe carotid reocclusion/restenosis at 30 days of randomization.

Data monitoring

Study monitoring to assure quality and conduct according to good clinical practice will be coordinated by the Clinical Investigation and Clinical Trials Unit from University Hospital Virgen del Rocio with a validated electronic data capture system. On-site or remote monitoring including source document verification will be conducted as necessary. Study personnel at each enrolling center are trained on the antiplatelet protocol, International Conference on Harmonisation of Technical Requirements for Registration of Pharmaceuticals for Human Use Good Clinical Practices, and investigator responsibilities.

Sample size calculation

In-stent thrombosis rates of 22% in the control arm and 10% in the experimental arm were estimated based on our pilot study.[4] An estimated total sample size of 240 patients (with 120 patients in each arm) should provide an 80% power to detect superiority in proportions of patients without in-stent thrombosis in the experimental group compared to the control group. Ten centers will participate, recruiting according to the volume of MT performed.

Statistical analyses

The analysis of the primary efficacy outcome includes both an intention-to-treat and per-protocol analysis. Superiority of tirofiban is established if the lower end of the above 95% confidence interval for the proportion of patients without in-stent thrombosis is greater than zero. The analysis of safety outcomes includes the proportion of patients with sICH and mortality and they will be compared between the tirofiban and the aspirin groups using binary logistic regression. Secondary outcomes will be carried out according to standard statistical principles for comparisons of parametric and non-parametric distribution, as appropriate. Subgroup analysis of patients will be performed based on age, sex, baseline ASPECTS, NIHSS score and IVT treatment. An interim analysis is not planned.

Ethical approval

Ethical approval for this study was obtained from University Hospital Virgen Macarena Ethical Committee (03/2022).

Study organization and funding

The sponsor of the trial is *Fundación Pública Andaluza para la Gestión en Investigación en Salud de Sevilla (FISEVI).* This project was funded by the Instituto de Salud Carlos III (ISCIII) through the project PI21/01322 and co-funded by the European Union. The Spanish Clinical Research Network (SCReN-Code:21.033) also contributed

to the study. The ITRIBiS project (Improving Translational Research Potential at the Institute of Biomedicine of Seville) has the registration number REGPOT-2013-1. M. Medina-Rodríguez was granted a Rio Hortega contract (CM21/00096). The project was included in the Cooperative Cerebrovascular Disease Research Network (INVICTUS) (RD16/0019/0015).

Discussion

Although stent placement is the best choice in terms of efficacy when performing a carotid angioplasty, there is not enough evidence to establish if carotid artery stenting is strictly recommended in the setting of an AIS due to TL. One of the main reasons for controversy is that the accurate AT strategy is yet to be elucidated. Carotid stenting in the setting of AIS has shown better recanalization rates[8–10] and patients had better functional outcomes when treated with IVT plus AT and stenting compared to those undergoing only carotid angioplasty. Improving cerebral blood flow and lower rates of carotid reocclusion are some of the benefits provided by stent placement.[11] However, the use of AT is needed to avoid early in-stent thrombosis, but it may increase the rate of sICH. The optimal balance within both events related to AT is the main concern to be solved in patients with TL.

Antithrombotic drugs during MT procedure, such as heparin alone, aspirin alone or their combination, have been previously associated with an increased risk of sICH without clinical benefits in a recent randomized clinical trial. Nevertheless, patients enrolled might not be comparable to our population study group because only 15.7% of patients had an extracranial internal carotid occlusion.[12]

On the other hand, without a suitable AT in-stent thrombosis may arise up to 22% of patients[11] and it is closely related to poor functional outcomes, higher rates of sICH and a poor neurological recovery.[11,13,14] The TITAN study demonstrated that the in-stent thrombosis is an independent predictor of sICH[13] and that both, single and dual AT, are associated with lower rates of intracranial hemorrhages. It may suggest that a good recanalization, including the maintenance of carotid artery patency, may prevent intracranial hemorrhages independently from IVT and AT. Although these data are based in retrospective studies, an optimal AT approach may allow stent placement, avoiding in-stent thrombosis and therefore, decreasing the risk of intracranial hemorrhages.

Options of AT may vary from single AT with aspirin, clopidogrel or intravenous glycoprotein IIb/IIIa inhibitor to a combination of them. Oral clopidogrel is difficult to administer during AIS. The most used AT is aspirin, with an intravenous administration available, although drug resistance rates may reach up to 30% of patients[15,16] and its administration is not recommended within first 90 min from IVT.[17] Tirofiban, an intravenous glycoprotein IIb/IIIa inhibitor, has an effect that do not depend on genetic polymorphisms and drug resistance is not described. Moreover, 90% platelet inhibition has been demonstrated ex-vivo in just 5 min[18] and has been used in some centers to prevent local platelet aggregation and early reocclusion.[19] Likewise, there is already preliminary evidence of the possible clinical benefit of the use of tirofiban, even when combined with rtPA, without an increase of intracranial hemorrhage or mortality.[20] In the view of these data, single AT with IV-tirofiban seems to be a reasonable option for patients with AIS due to TL.

Conclusions

This is the first trial that will explore the efficacy and safety of IV-tirofiban specifically in patients with AIS due to TL. As TL may represent up to 20% of AIS, an appropriate antiplatelet management is needed to balance the risk of intracranial hemorrhage and in-stent thrombosis. If positive, this therapeutic approach will significantly improve reperfusion rates in patients with tandem lesions and will confer a long-lasting carotid patency.

Acknowledgements

ATILA is an investigator-led clinical trial. The sponsor of the trial is the *Fundación Pública Andaluza para la Gestión en Investigación en Salud de Sevilla.* This study have been funded by the Instituto de Salud Carlos III (ISCIII) through the project PI21/01322 (Co-funded by the European Regional Development fund/European Social Fund “Away to make Europe”/Investing in your future”).

Declaration of conflicting interests

The author(s) declared no potential conflicts of interest with respect to the research, authorship, and/or publication of this article.

Funding

The author(s) disclosed receipt of the following financial support for the research, authorship, and/or publication of this article: This project was funded by the Instituto de Salud Carlos III (ISCIII) through the project PI21/01322 and co-funded by the European Union. The Spanish Clinical Research Network (SCReN-Code:21.033) also contributed to the study. The ITRIBiS project (Improving Translational Research Potential at the Institute of Biomedicine of Seville) has the registration number REGPOT-2013-1. M. Medina-Rodríguez was granted a Rio Hortega contract (CM21/00096). The project was included in the Cooperative Cerebrovascular Disease Research Network (INVICTUS) (RD16/0019/0015).

Ethical approval

Ethical approval for this study was obtained from University Hospital Virgen Macarena Ethical Committee (03/2022).

Informed consent

Written informed consent will be obtained from all subjects before randomization.

Guarantor

EZA

Contributorship

EZA, FM, AG and MMR researched literature, conceived the study and designed the trial. MMR wrote the manuscript. FM and EZA supervised and edited the manuscript. All authors reviewed and approved the final version of the manuscript.

Trial Registration

NCT0522596.
Eudra-CT: 2021-003874.30

ORCID iDs

Manuel Medina-Rodríguez https://orcid.org/0000-0002-2862-9586
Asier de Albóniga-Chindurza https://orcid.org/0000-0002-3770-8534
Juan Antonio Cabezas-Rodríguez https://orcid.org/0000-0001-9035-4643
Irene Escudero-Martínez https://orcid.org/0000-0003-1838-0235
Laura Amaya Pascasio https://orcid.org/0000-0003-1162-4209
Jose María Ramirez Moreno https://orcid.org/0000-0001-5061-1510
Joan Montaner https://orcid.org/0000-0003-4845-2279

References

1. Assis Z, Menon BK, Goyal M, et al. Acute ischemic stroke with tandem lesions: technical endovascular management and clinical outcomes from the ESCAPE trial. *J Neurointerv Surg* 2018; 10: 429–433.
2. Kim YS, Garami Z, Mikulik R, et al. Early recanalization rates and clinical outcomes in patients with tandem internal carotid artery/middle cerebral artery occlusion and isolated middle cerebral artery occlusion. *Stroke* 2005; 36: 869–871.
3. Anadani M, Spiotta AM, Alawieh A, et al. Emergent carotid stenting plus thrombectomy after thrombolysis in tandem strokes: analysis of the TITAN registry. *Stroke* 2019; 50: 2250–2252–52.
4. Zapata-Arriaza E, de Albóniga-Chindurza A, Ortega-Quintanilla J, et al. Clinical outcomes of mechanical thrombectomy in stroke tandem lesions according to intracranial occlusion location. *J Stroke* 2021; 23: 124–127.
5. Powers WJ, Rabinstein AA, Ackerson T, et al. 2018 guidelines for the early management of patients with acute ischemic stroke: a guideline for healthcare professionals from the American heart association/American stroke association. *Stroke* 2018; 67: 1934–e418.
6. Goyal M, Menon BK, van Zwam WH, et al. HERMES collaborators. endovascular thrombectomy after large- vessel ischaemic stroke: a meta-analysis of individual patient data from five randomised trials. *Lancet* 2016; 387: 1723–1731.
7. Hacke W, Kaste M, Fieschi C, et al. Intravenous thrombolysis with recombinant tissue plasminogen activator for acute hemispheric stroke. The European cooperative acute stroke study (ECASS). *JAMA* 1995; 274: 1017–1025.
8. Gory B, Haussen DC, Piotin M, et al. Impact of intravenous thrombolysis and emergent carotid stenting on reperfusion and clinical outcomes in patients with acute stroke with tandem lesion treated with thrombectomy: a collaborative pooled analysis. *Eur J Neurol* 2018; 25: 1115–1120.
9. Sadeh-Gonik U, Tau N, Friehmann T, et al. Thrombectomy outcomes for acute stroke patients with anterior circulation tandem lesions: a clinical registry and an update of a systematic review with meta-analysis. *Eur J Neurol* 2018; 25: 693–700.
10. Eker OF, Bühlmann M, Dargazanli C, et al. Endovascular treatment of atherosclerotic tandem occlusions in anterior circulation stroke: technical aspects and complications compared to isolated intracranial occlusions. *Front Neurol* 2018; 9: 1046.
11. Renú A, Blasco J, Laredo C, et al. Carotid stent occlusion after emergent stenting in acute ischemic stroke: incidence, predictors and clinical relevance. *Atherosclerosis* 2020; 313: 8–13.
12. van der Steen W, van de Graaf RA, Chalos V, et al. Safety and efficacy of aspirin, unfractionated heparin, both, or neither during endovascular stroke treatment (MR CLEAN-MED): an open-label, multicentre, randomised controlled trial. *Lancet* 2022; 399: 1059–1069.
13. Zhu F, Bracard S, Anxionnat R, et al. Impact of emergent cervical carotid stenting in tandem occlusion strokes treated by thrombectomy: a review of the TITAN collaboration. *Front Neurol* 2019; 10: 206.
14. Wallocha M, Chapot R, Nordmeyer H, et al. Treatment methods and early neurologic improvement after endovascular treatment of tandem occlusions in acute ischemic stroke. *Front Neurol* 2019; 10: 127.
15. Bennett D, Yan B, Macgregor L, et al. A pilot study of resistance to aspirin in stroke patients. *J Clin Neurosci* 2008; 15: 1204–1209.
16. Zheng AS, Churilov L, Colley RE, et al. Association of aspirin resistance with increased stroke severity and infarct size. *JAMA Neurol* 2013; 70: 208–213.
17. Zinkstok SM and Roos YB. Early administration of aspirin in patients treated with alteplase for acute ischaemic stroke: a randomised controlled trial. *Lancet* 2012; 380: 731–737.
18. Zhao W, Che R, Shang S, et al. Low-dose tirofiban improves functional outcome in acute ischemic stroke patients treated with endovascular thrombectomy. *Stroke* 2017; 48: 3289–3294.
19. Sun L, Zhang J, Song Y, et al. Safety and efficacy of prophylactic tirofiban infusion for acute intracranial intraprocedural stent thrombosis. *Sci Rep* 2021; 11: 21326.
20. Pan X, Zheng D, Zheng Y, et al. Safety and efficacy of tirofiban combined with endovascular treatment in acute ischaemic stroke. *Eur J Neurol* 2019; 26: 1105–1110.
